# New Improved cGMP Analogues to Target Rod Photoreceptor Degeneration

**DOI:** 10.1021/acs.jmedchem.4c00586

**Published:** 2024-04-30

**Authors:** Oswaldo Pérez, Agnese Stanzani, Li Huang, Nicolaas Schipper, Thorsteinn Loftsson, Martin Bollmark, Valeria Marigo

**Affiliations:** † Chemical Processes and Pharmaceutical Development Research Institutes of Sweden, Forskargatan 20 J, 15136 Södertälje, Sweden; ‡ Faculty of Pharmaceutical Sciences, University of Iceland, Hofsvallagata 53, 107 Reykjavik, Iceland; § Department of Life Sciences, University of Modena and Reggio Emilia, via Campi 287, 41125 Modena, Italy

## Abstract

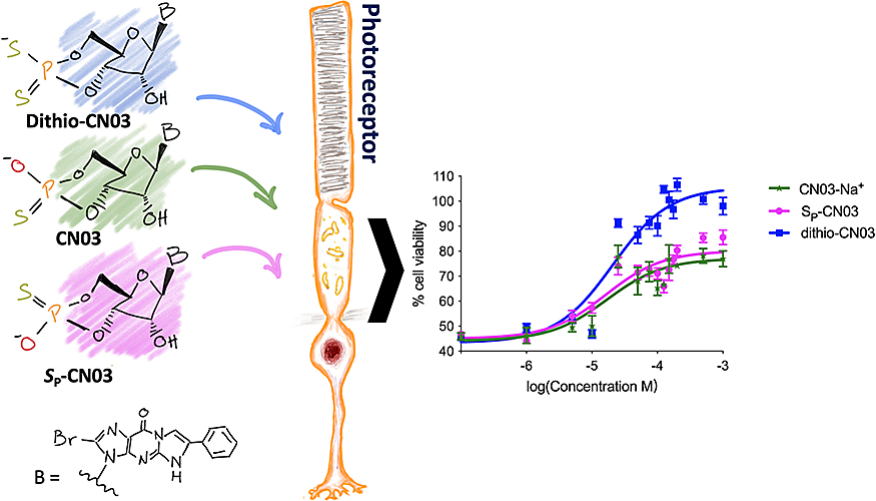

Retinitis pigmentosa (RP) is a form of retinal degeneration affecting a young population with an unmet medical need. Photoreceptor degeneration has been associated with increased guanosine 3′,5′-cyclic monophosphate (cGMP), which reaches toxic levels for photoreceptors. Therefore, inhibitory cGMP analogues attract interest for RP treatments. Here we present the synthesis of dithio-CN03, a phosphorodithioate analogue of cGMP, prepared using the H-phosphonothioate route. Two crystal modifications were identified as a trihydrate and a tetrahydrofuran monosolvates. Dithio-CN03 featured a lower aqueous solubility than its *R*_P_-phosphorothioate counterpart CN03, a drug candidate, and this characteristic might be favorable for sustained-release formulations aimed at retinal delivery. Dithio-CN03 was tested in vitro for its neuroprotective effects in photoreceptor models of RP. The comparison of dithio-CN03 to CN03 and its diastereomer *S*_P_-CN03, and to their phosphate derivative oxo-CN03 identifies dithio-CN03 as the compound with the highest efficacy in neuroprotection and thus as a promising new candidate for the treatment of RP.

## Introduction

Retinitis pigmentosa (RP) is an inherited disease characterized by the loss of retinal photoreceptor cells and is a form of retinal degeneration that can eventually lead to blindness.^1,2^ Some of the RP clinical signs are nyctalopia, peripheral visual field loss, and characteristic fundus changes (i.e., bone spicules and hypo- or hyperpigmentation, arteriolar narrowing, and waxy disc pallor) and vision loss. Nonsyndromic RP is one of the primary causes of visual disability and blindness in people under 60 years of age, with a prevalence of approximately 1:4000 people worldwide.^3^ RP can be inherited as autosomal dominant (30–40% cases), autosomal recessive (50–60% cases) or X-linked (5–15% cases) traits.^4^ One of the major challenges in the treatment of RP is the high genetic heterogeneity (disease causing mutations have been found in over 200 genes, https://sph.uth.edu/retnet), necessitating expensive personalized strategies to treat a small number of patients. However, studies in cellular and animal models of the disease identified increased levels of guanosine 3′,5′-cyclic monophosphate (cGMP) as a common event in photoreceptor cell death during RP progression.^5,6^

cGMP in photoreceptors has two main targets: on one hand, it acts as a second messenger in phototransduction by promoting the opening of the cyclic nucleotide-gated channels (CNGC), allowing Na^+^ and Ca^2+^ influx and thus changes in photoreceptor membrane potential; on the other hand, it activates the cGMP-dependent protein kinase G (PKG).^7^ The two targets and their contribution to photoreceptor degeneration were independently characterized. High cGMP maintains CNGC opening, causing high intracellular Ca^2+^ that is toxic for photoreceptors. Reduced functional CNGC could, in fact, preserve the retina from degeneration in conditions with high cGMP.^8^ Experimental PKG activation in wild type retina was demonstrated to promote photoreceptor degeneration.^9^ Based on these premises, in a previous study we have tested several cGMP analogues with inhibiting activity to CNGC and PKG. The analogues with the *R*_P_-configurated phosphorothioate substitution (*R*_P_-cGMPS) were identified as PKG inhibitors, and derivatives thereof containing the β-phenyl-1,N^2^-etheno-modification (PET) were found to also inhibit CNGC. We thus chose to focus on analogues carrying both these characteristics and that could act on PKG and CNGC. We identified *R*_P_-8-Br-PET-cGMPS (CN03) as a cGMP analogue able in vitro and in vivo to interfere with PKG activity and to block Ca^2+^ influx through the CNGC.^10^ Importantly, when in vivo delivered, CN03 could preserve photoreceptor cells of murine models of RP. Therefore, CN03 was selected as drug candidate for further pharmaceutical development for treatment of RP.^11^

A scalable synthetic process for CN03 manufacture was first developed using the H-phosphonate chemistry.^11^ The process achieved control of the primary potential side products, namely, the corresponding *S*_P_-phosphorothioate diastereomer (*S*_P_-CN03) as well as the corresponding phosphate derivative (oxo-CN03). It was also successful in replacing chromatographic purifications for crystallizations, allowing, for the first time, the output of a cGMP analogue on larger scales and facilitating the next steps of pharmaceutical development and translation to clinical trials.

This work was followed by an innovative investigation exploring new salts and crystal modifications of CN03, and their preparations.^12^ We searched for forms with low aqueous solubility, which may prolong the duration of action of the new drug and open the possibility of new formulations. This was important to moderate the frequency of drug application to patients, since the most common routes of administration for drugs targeted to retinal photoreceptors are repeated intravitreal or subretinal injections.^13^ We confirmed that the solubility of the drug compound could be significantly reduced by changing the counterions.^12^

In this paper, inspired by the success of *R*_P_-phosphorothioate CN03 as a drug candidate for RP treatment, we investigated the viability of a new CN03 analogue, namely, the corresponding phosphorodithioate, henceforth referred to as dithio-CN03. Phosphorodithioates are thought to resist cleavage by phosphodiesterases to a higher degree than their phosphoro(mono)thioate counterparts.^14−16^ This could translate to a longer half-life in photoreceptor cells and therefore a longer duration of action. However, nucleoside 3′,5′-cyclic phosphorodithioates, specifically, have seldom been explored in the literature. We also sought to prepare two potential key impurities: the corresponding *S*_P_-phosphorothioate (*S*_P_-CN03) and phosphate (oxo-CN03). These compound classes have been described as PKG agonists^17^ and, thus, possibly promote photoreceptor degeneration, but this had not been directly tested.

Here, we present the synthesis of dithio-CN03, *S*_P_-CN03, and oxo-CN03, using the chemistry developed for larger-scale CN03 manufacture. We then provide evidence that dithio-CN03 is not only more effective than CN03 in protecting photoreceptors from cell death caused by high cGMP levels but also features physicochemical properties favorable for the development of sustained-release formulations.

## Results and Discussion

### Preparation of Dithio-CN03 and Key Impurities

**Scheme 1:**
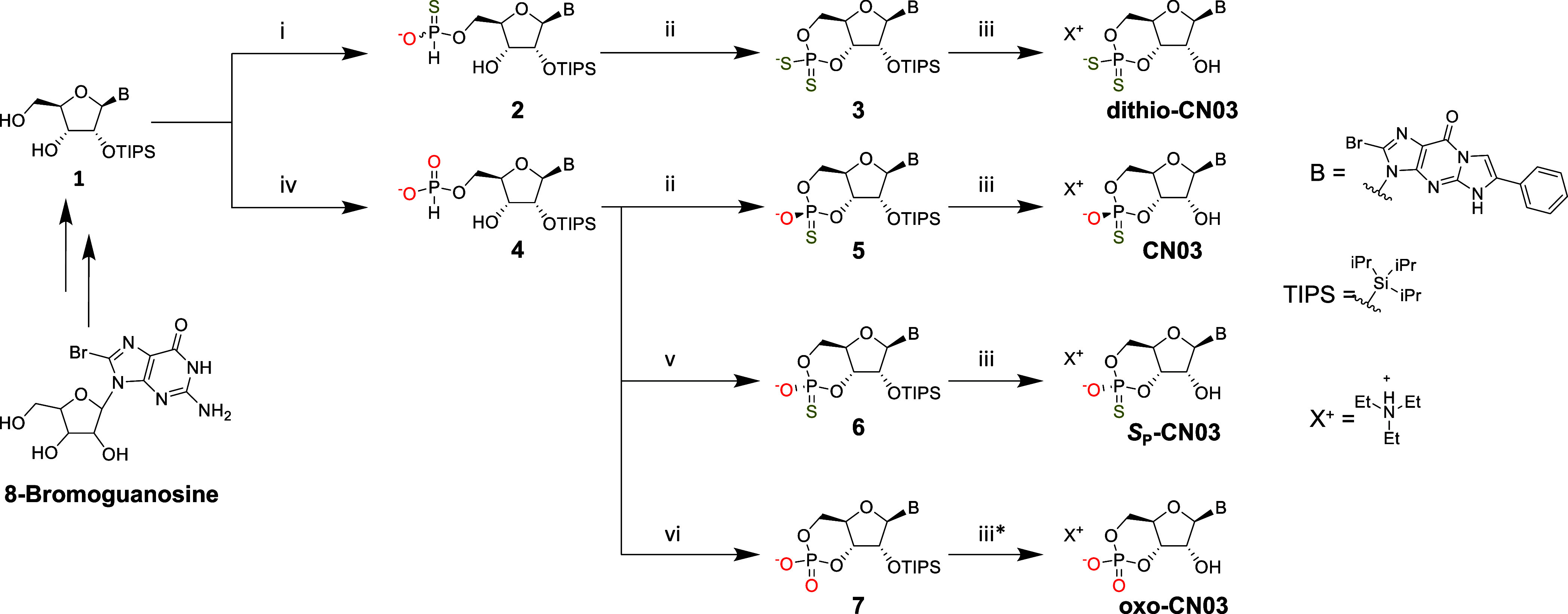
Synthetic Route to CN03 and Analogues (i) TEA-phosphinate, pyridine → Pv-Cl, S_(s)_, and TEAA; (ii) Pv-Cl, 2,6-lutidine, DCM → S_(s)_ and Et_3_N; (iii) Et_3_N·3HF, and THF; (iv) DPP, pyridine/DCM → H_2_O and Et_3_N; (v) Pv-Cl, pyridine → S_(s)_ and Et_3_N; and (vi) Pv-Cl, 2,6-lutidine, DCM → I_2(s)_ and H_2_O. *The solvent was MeOH instead of THF. The sequence from 8-bromoguanosine to CN03 has been performed in ∼700 g scale.

We elected to use the same strategy that we previously developed for CN03 for synthesis of dithio-CN03, *S*_P_-CN03, and oxo-CN03, namely coupling of a P(III) reagent at the 5′–OH followed by internal cyclization and oxidation to the 3′,5′-cyclic P(V) diester.^11^ By using this route, we could make use of the same starting materials and reagents that were already available in our laboratory from previous manufacture of CN03. Thus, the first two steps leading to 2′–OH-protected intermediate **1**, as well as the final step remained unchanged. Because the final step affords all of the products as salts of triethylammonium (TEA), from now on we define CN03 and its analogues as TEA salts, unless otherwise specified. Scheme 1 summarizes the synthetic route toward each product.

We previously described *S*_P_-CN03 as the main side-product during cyclization of the 5′-H-phosphonate in synthesis of CN03.^11^ As reported by Rozniewska and co-workers, generation of the *R*_P_-H-phosphonate intermediate (which oxidizes with sulfur to form the corresponding *S*_P_-phosphorothioate) was thermodynamically favored over the desired diastereomer.^18^ In that case, we preferentially obtained the kinetic product by slowing the epimerization with 2,6-lutidine and quenching the reaction as early as possible. Thus, we obtained the *S*_P_-phosphorothioate **6** by employing pyridine as the solvent and allowing the mixture to age toward equilibrium before quenching. Conversely, to obtain cyclic phosphate **7**, we employed the strategy for *O*-oxidation of cyclic H-phosphonates toward cyclic phosphates described by the same group.^18^ When we followed their procedure, quenching of the reaction mixture with I_2_ and H_2_O, instead of triethylamine and sulfur, the desired cyclic phosphate **7** was obtained. Unlike compounds **3**, **5**, and **6**, cyclic phosphate **7** displayed low solubilities in tetrahydrofuran (THF), methyl tetrahydrofuran (MeTHF), and acetonitrile (MeCN). We therefore performed the following 2′–OH deprotection step in methanol.

For synthesis of dithio-CN03, we started by generating the 5′-H-phosphonothioate **2** using a strategy described by Stawinski et al.^19^ and Liu et al.^20^ This strategy involves coupling TEA-phosphinate at the nucleoside 5′–OH using pivaloyl chloride (Pv-Cl) to afford a H-phosphinate monoester as an intermediate. This is followed by sulfurization in situ to the corresponding H-phosphonothioate. In the published studies, the starting guanosine derivative featured protecting groups at the 2′- and 5′-hydroxyls, and they obtained a 3′-H-phosphonothioate. Differently, our starting guanosine substrate **1** was unprotected at 3′ and 5′. Therefore, we instead obtained 5′-H-phosphonothioate **2** as the main product (as a diastereomer pair, Figures S6 and S7), and we observed no significant side reactions.

3′,5′-cyclic nucleoside phosphorodithioate preparations have been scarcely reported in the literature,^21,22^ but none of these studies used the H-phosphonothioate monoester approach. On the other hand, the condensation of a nucleoside H-phosphonothioate monoester with a second nucleoside using Pv-Cl was known to afford a dinucleoside H-phosphonothioate.^23^ Since the methodology was similar to the one for the internal 3′ → 5′ coupling of H-phosphonates to afford 3′,5′-cyclic H-phosphonate diesters, we performed the following cyclization of 5′-H-phosphonothioate **2** in the same manner as we previously had done for 5′-H-phosphonate **4** for preparation of CN03.

The starting material was consumed within the first analysis (<5 min), and the expected intermediate cyclic H-phosphonothioate diastereomers, with resonances in the 60–70 ppm range, were present as the major products. The reaction mixture remained stable until addition of TEA and sulfur after 60 min. Immediately, signals from H-phosphonothioate intermediates were replaced by resonances at 112 ppm from the desired phosphorodithioate. The product **3** could again be isolated in over 99% liquid chromatography-purity by single straight-phase column chromatography (Figure S8).

Finally, deprotection of the 2′-silyl group in a mixture of THF and TEA·3HF behaved similarly to the procedure described for the corresponding phosphorothioates, with no observable side reactions. The deprotected dithio-CN03 was also found to precipitate from the reaction mixture over the course of 3 days. The product was washed with THF and dried under a vacuum at 50 °C. Nuclear magnetic resonance (NMR, Figure S23) analyses confirmed the product as the TEA salt of dithio-CN03 and revealed some THF present at near-stoichiometric amounts (8% w/w) despite the drying procedure. A broad water peak that was not possible to quantify was also present. Finally, the product was found to be crystalline by X-ray powder diffraction (XRPD) and labeled as crystal modification I (Figure S22).

This represented a first proof of concept for the H- phosphonothioate route as a procedure to facile preparation of 3′,5′-cyclic phosphorodithioates such as dithio-CN03, which could be further improved in terms of scale and of isolation of intermediates by chromatography.

### Solid-State Characterization of Dithio-CN03

Physicochemical properties of dithio-CN03 were characterized and compared to CN03. First, its solubility was measured in deionized H_2_O at room temperature, giving an aqueous solubility of 1.17 (±0.02) mg/mL, or 1.8 mM (Table S1). For sustained release of CN03 in the vitreous, it may be beneficial to study salt forms with a lower solubility. Salt forms of CN03, for example, were prepared varying in aqueous solubility as low as 0.01 mg/mL.^12^ To facilitate future development of reactive crystallizations toward other salt forms, a temperature-dependent solubility was also measured between 20 and 75 °C. The resulting solubility curve can be found in the Supporting Information (Figure S38).

**Figure 1:**
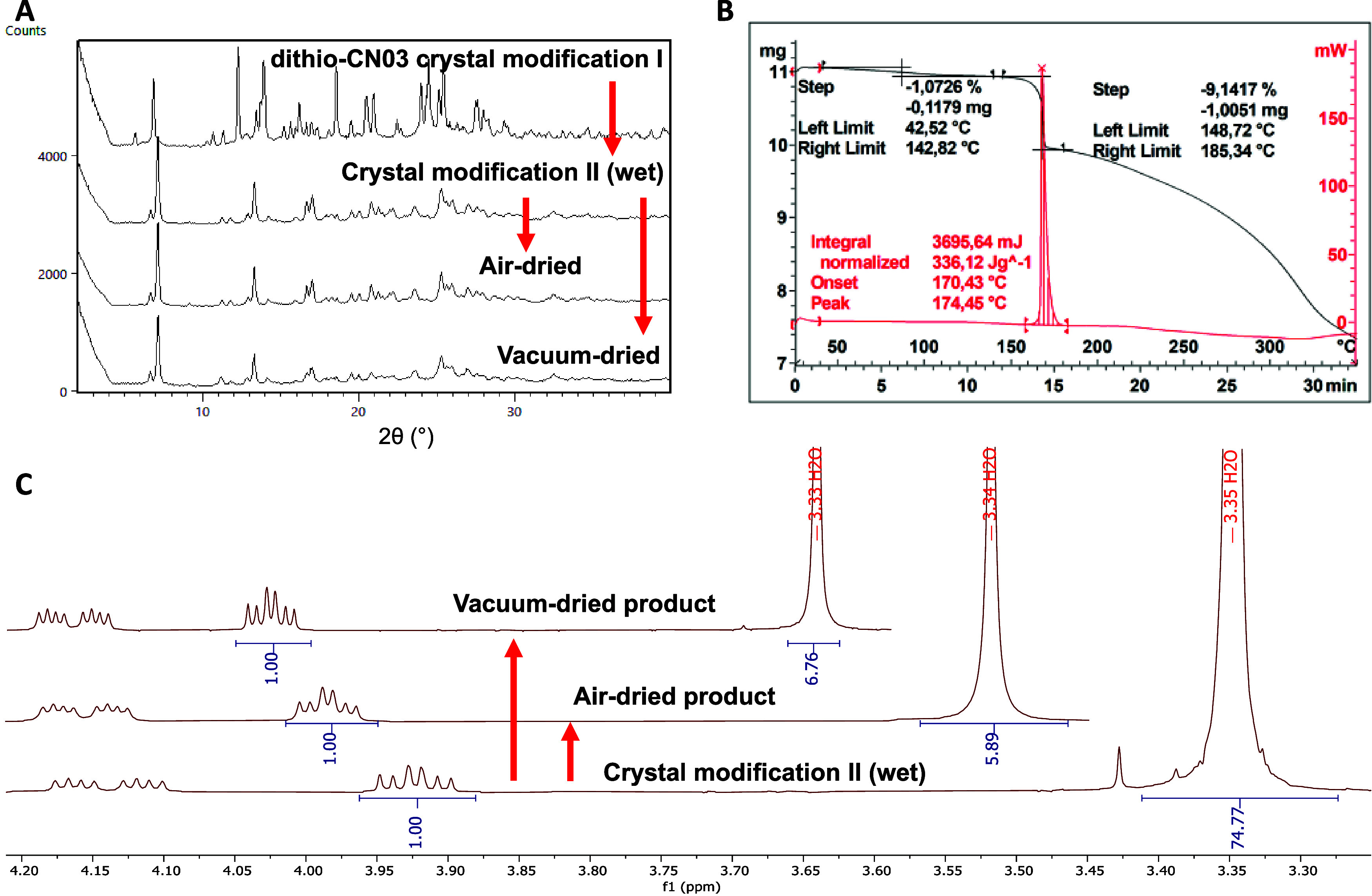
Solid-state characterization of dithio-CN03. (A) Diffractograms of dithio-CN03 before aqueous slurry (first), the wet material after aqueous slurry (second), and the final material after drying with different methods (third and fourth). (B) TG and DSC curves of dithio-CN03. TG is the topmost curve shown in black. DSC is the bottom curve shown in red. (C) Expanded ^1^H NMR spectra of dithio-CN03 after the aqueous slurry. Insets show the level of H_2_O after drying with different methods.

The XRPD pattern of the wet material was also recorded during the solubility studies. The analysis returned a new unique diffraction pattern after slurring in water, identified as crystal modification II. Thus, the crystal modification I of dithio-CN03 underwent a recrystallization in water to crystal modification II, and the aqueous solubility measured for dithio-CN03 specifically corresponded to the latter (Figure 1A). The solids were then monitored with XRPD as they dried to record any further phase transitions due to loss of crystal water. Separate samples were dried in open air for 3 days, as well as at 55 °C in a vacuum oven over one night, but the diffraction pattern remained unchanged in both cases. Subsequent ^1^H NMR analyses of the air-dried and vacuum-dried samples found close to 3 equiv of H_2_O on both (Figure 1C). We therefore concluded that crystal modification II is a stable trihydrate.

Finally, we performed thermal analyses on crystal modification I by using differential scanning calorimetry (DSC) and thermogravimetry (TG), both of which confirmed a behavior analogous to that of CN03. There were no observable melting points. Instead, there was only a sharp exothermic event on DSC at 170 °C associated with significant mass loss on TG, which we interpreted as a thermal decomposition of the compound (Figure 1B). It should be noted that decomposition peaks on DSC and TG are not indicative of physical stability (e.g., melting point, and solubility). Since the material was crystalline, it was considered to own a melting point at a higher temperature than this decomposition.

The mass loss observed on TG from low-boiling solvents was only 1% w/w of the sample (observed between 25 and 150 °C, Figure 1B). This did not account for all of the THF measured by NMR assay, which amounted to 8% w/w of the material. The fact that this THF was not lost to evaporation was indicative of it being a solvent of crystallization, and we therefore concluded that this crystal modification I was most likely a stable THF monosolvate. On the other hand, the water observed on NMR might be attributed to the hygroscopicity of the solvent used for the analysis (DMSO-d_6_). Since Karl Fischer titration is unable to determine water content for phosphorothioates due to side reactions between sulfurs and the reagent, we currently cannot make conclusions on whether crystal modification I is also hydrated.

Encouraged by the promisingly lower solubility of dithio-CN03 compared to CN03, we proceeded with further analyses on functionality in protecting photoreceptor cells from cell death.

### Assessment of Possible Toxicity of CN03 Analogues to Photoreceptor Cells

**Figure 2:**
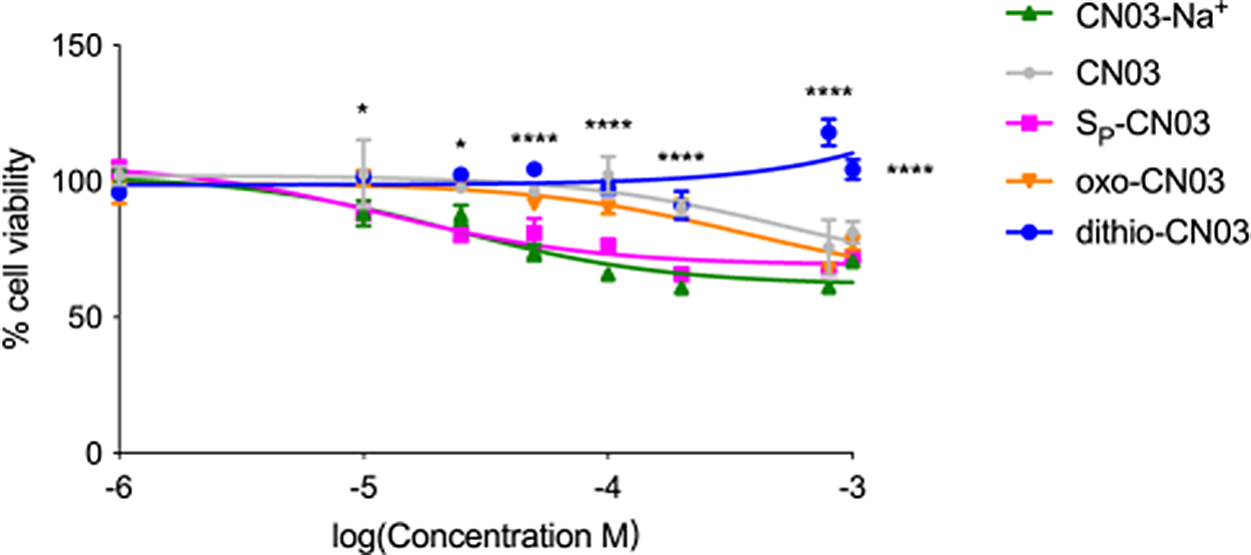
Toxicity assay of CN03 analogues. 661W-A11 cells were exposed to increasing concentrations of CN03 analogues (from 1 μM to 1 mM) and viability was assessed. Untreated cells were set at 100% viability. CN03-Na^+^ (green line), CN03 (gray line), *S*_P_-CN03 (pink line), oxo-CN03 (orange line), and dithio-CN03 (blue line). Statistical comparison of dithio-CN03 to CN03-Na^+^: Dunnett’s two-way ANOVA * *P* < 0.05, **** *P* < 0.0001.

With the aim of developing a new treatment for RP, we evaluated possible toxic effects of the cGMP analogues on a retinal cell line. The target of the treatment for RP is rod photoreceptors, the cells that die due to increased intracellular levels of cGMP during the progression of retinal degeneration. We chose a genetically modified cell line, called 661W-A11, that we demonstrated to display several features of rod photoreceptors.^24^ Furthermore, this cell line can be stressed with zaprinast, a phosphodiesterase 6 (PDE6) inhibitor, to increase intracellular cGMP and activate cell death pathways, as found in the RP mutant retina. This procedure was demonstrated to mimic the retinal degeneration process.^24^ A toxicity test was set up by exposing 661W-A11 cells to increasing doses of the four new TEA salts of CN03: CN03, *S*_P_-CN03, oxo-CN03, and dithio-CN03 (Figure 2). Viability of the cells was assessed by an MTT assay that measures cell metabolic activity. Furthermore, because previous studies have primarily investigated CN03 as a sodium salt (CN03-Na^+^), we accounted for any potential effect imparted by the counterion itself by including CN03-Na^+^ in our tests.

Due to the low aqueous solubility of dithio-CNO3, we were unable to achieve sufficient concentrations for our study when diluted in the aqueous cell culture medium. Thus, we instead used dithio-CN03 dissolved in DMSO to be diluted in the cell culture medium. We thus compared the viability of cells treated with dithio-CN03 to cells treated with equal amounts of DMSO. We observed that dithio-CN03 was the compound that showed the lowest toxicity.

### Effects of CN03 Analogues in In Vitro Cellular Models of Photoreceptor Degeneration

**Figure 3:**
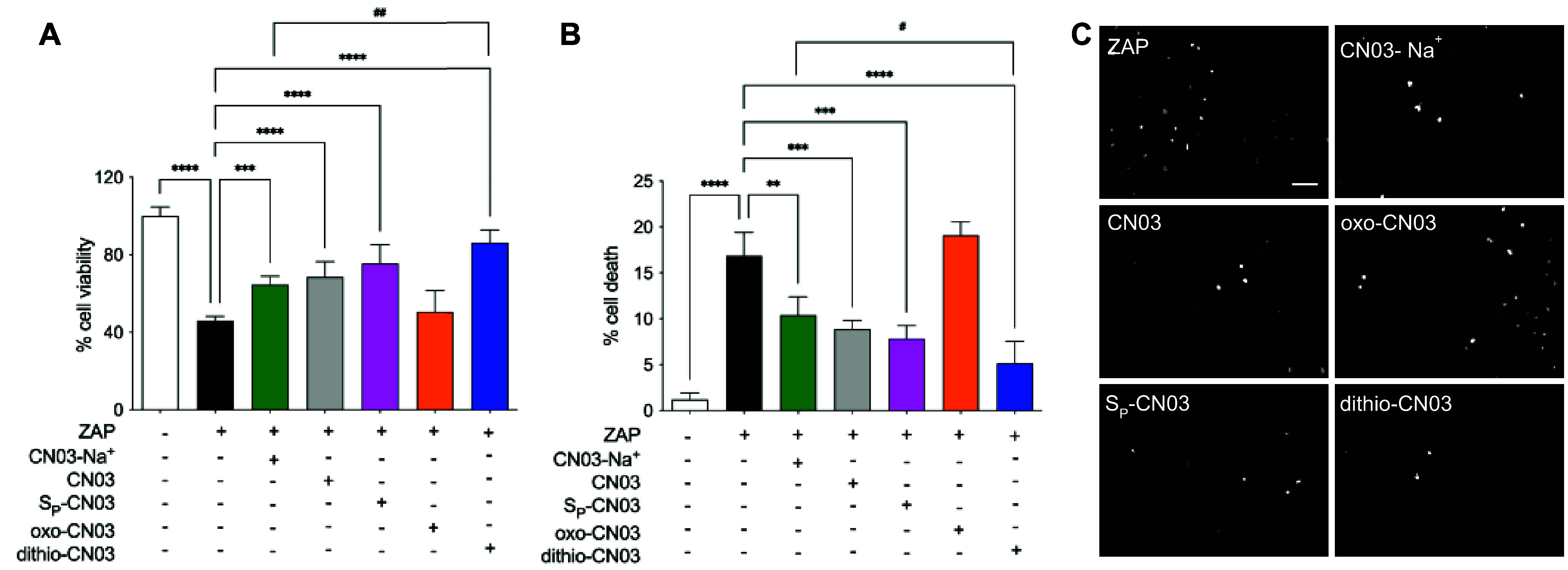
(A) Cell viability was assessed by MTT assay in 661W-A11 cells stressed with 200 μM zaprinast for 48 h (black bar) and treated with 50 μM CN03-Na^+^ (green bar), CN03 (gray bar), *S*_P_-CN03 (pink bar), oxo-CN03 (orange bar), and dithio-CN03 (blue bar). Percentage of cell viability was calculated by normalizing viability of cells treated with CN03 compounds to cells treated with the corresponding vehicle, which was set as 100% viability (white bar). (B) Cell death was assessed by TUNEL assay in 661W-A11 cells stressed with 200 μM zaprinast for 48 h (black bar) and treated with 50 μM CN03-Na^+^ (green bar), CN03 (gray bar), *S*_P_-CN03 (pink bar), oxo-CN03 (orange bar), dithio-CN03 (blue bar), and untreated (white bar). Percentage of cell death is presented as ratio of TUNEL^+^ cells over total number of cells. (C) Micrographs as examples of TUNEL staining for data shown in panel B. White dots are cells labeled with TUNEL, thus undergoing cell death. Scale bar 50 μm. Statistical comparison: one way ANOVA ** *P* < 0.01, *** *P* < 0.001, and **** *P* < 0.0001 and Student’s *t* test unpaired two-tailed to compare dithio-CN03 to CN03-Na^+#^*P* < 0.05, ^##^*P* < 0.01.

The neuroprotective activity of the newly developed compounds was then evaluated in 661W-A11 cells stressed with zaprinast. Viability was assessed by an MTT assay. All CN03 compounds, except oxo-CN03, were able to improve cell viability. Remarkably, dithio-CN03 exhibited significantly higher protective effects compared to CN03-Na^+^. Meanwhile, the two salts of CN03 and *S*_P_-CN03, while increasing cell viability, did not significantly differ from each other in their protective effects (Figure 3A).

Viability assay cannot discriminate between protection from cell death and possible increased proliferation induced by the treatment. To discriminate between these two responses, we analyzed cell death by the TUNEL assay that detects dying cells based on fragmented chromatin. The TUNEL assay confirmed data obtained with the MTT assay (Figure 3B,C). The failure of oxo-CN03 to protect 661W-A11 cells from stress with zaprinast was expected because the oxo-form of a cGMP analogue does not act as an inhibitor of the cGMP targets. Based on viability and cell death assays, we defined that dithio-CN03 owned the highest neuroprotective activity.

**Figure 4:**
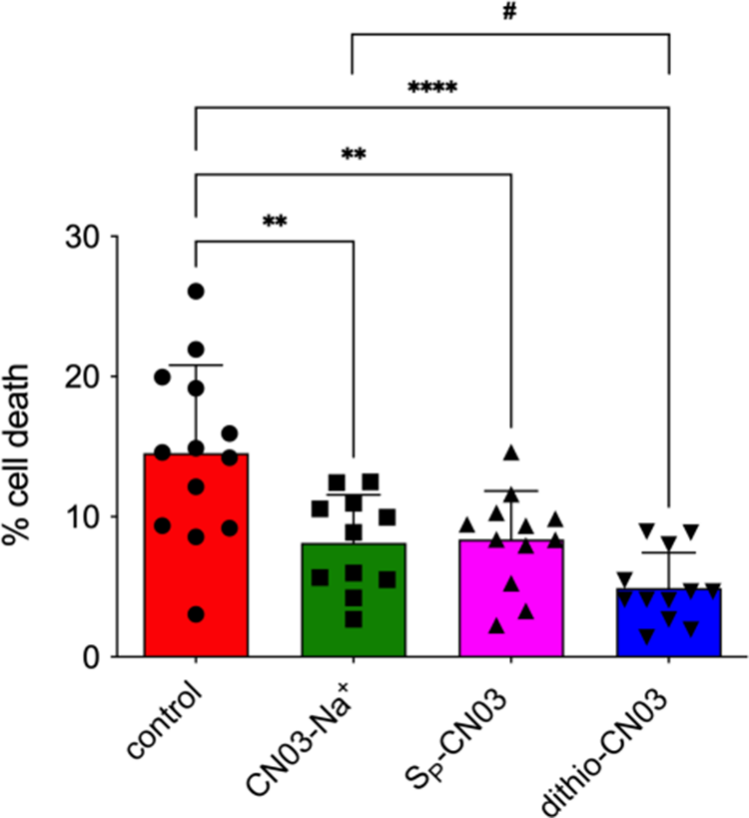
Cell death was assessed by the TUNEL assay in primary photoreceptor cells derived from *rd1* mutant mice. Cells were differentiated in vitro and underwent spontaneous cell death due to the loss of function mutation in the *Pde6b* gene. Cell death was assessed after 24 h exposure to CN03-Na^+^ (green bar), *S*_P_-CN03 (pink bar), dithio-CN03 (blue bar), and vehicle (control, red bar). Statistical comparison: one-way ANOVA ** *P* < 0.01, *** *P* < 0.001, and **** *P* < 0.0001 and Student’s *t* test unpaired two-tailed to compare dithio-CN03 to CN03-Na^+#^*P* < 0.05.

Although results that were acquired with the new in vitro RP model yielded interesting perspectives for dithio-CN03, we recognized that the stress with zaprinast on the 661W-A11 cells could also inhibit mitochondrial pyruvate transport^25^ or activate other yet uncharacterized events. To address this possible limitation of the RP model, we tested the CN03 analogues in another RP in vitro cellular model, previously validated for the analysis of cGMP analogues.^10^ This model is based on the primary cell culture and differentiation of cells derived from *rd1* mutant eyes. The *rd1* mouse is one of the best characterized murine models of RP. It carries a mutation in the *Phosphodiesterase 6b* (*Pde6b*) gene. Lack of PDE6 function causes high intracellular levels of cGMP in rod photoreceptor cells.^26,9^ We previously demonstrated that in vitro differentiation into rod photoreceptors of retinal stem cells derived from *rd1* eyes could induce spontaneous cell death at the 11th day of differentiation. Furthermore, we demonstrated that *rd1* mutant photoreceptors in vitro activated cell death pathways as found in the degenerating retina in vivo.^27^ CN03-Na^+^, *S*_P_-CN03, and dithio-CN03 were administered to the culture of *rd1* mutant cells at a concentration of 50 μM at day 10 of differentiation. The concentration of the compounds and the time of treatment were chosen based on the previously published study in which cGMP inhibitory analogues were tested in this type of primary photoreceptors.^10^ Cell death was then analyzed by the TUNEL assay at day 11 of in vitro differentiation. CN03-Na^+^ decreased the percentage of cell death, as expected and as previously reported.^10^ Interestingly, dithio-CN03 showed significantly better neuroprotective activity also in this RP in vitro model when compared to CN03-Na^+^. Finally, it was intriguing to note that *S*_P_-CN03 was not toxic to photoreceptors as previously assumed,^17^ but in fact had a neuroprotective effect similar to CN03-Na^+^ (Figure 4). These data identify dithio-CN03 as a new and more effective compound compared with the previously published CN03- Na^+^.

The doses used in this experiment were in the μM range and may appear to be quite high. It should be kept in mind that cyclic nucleotides are hydrophilic due to a negative charge of their cyclic phosphate unit, thus cell membrane permeability is expected to be in the range of 10–30% of the extracellular applied concentration.^28^ However, dithio-CN03 may have enhanced permeability to the cells since the sulfur substitution at the phosphate moiety is expected to increase lipophilicity. This is reflected in the calculated LogD_7.4_ values (MarvinSketch Software) for the oxo-CN03, *R*_P_-/*S*_P_-CN03, and dithio-CN03 (respectively −1.26, −0.37, and 1.21), with lower solubility found for the latter. With the perspective of translating the CN03 analogues to the clinic for the treatment of RP patients, these chemicophysical characteristics may impact the delivery of the drug to the retina. In fact, the half-life of a dissolved low-molecular weight drug in the vitreous humor is less than 10 h.^29^ Thus, dithio-CN03 would be excreted rapidly after intravitreal injection. Alternatively, dithio-CN03 might be more suitable for topical administration, since higher lipophilicity and lower aqueous solubility might enhance its topical availability. Future drug development will need to evaluate appropriate formulations for a more efficient in vivo delivery of dithio-CN03 to photoreceptor cells in the human eye.

**Figure 5:**
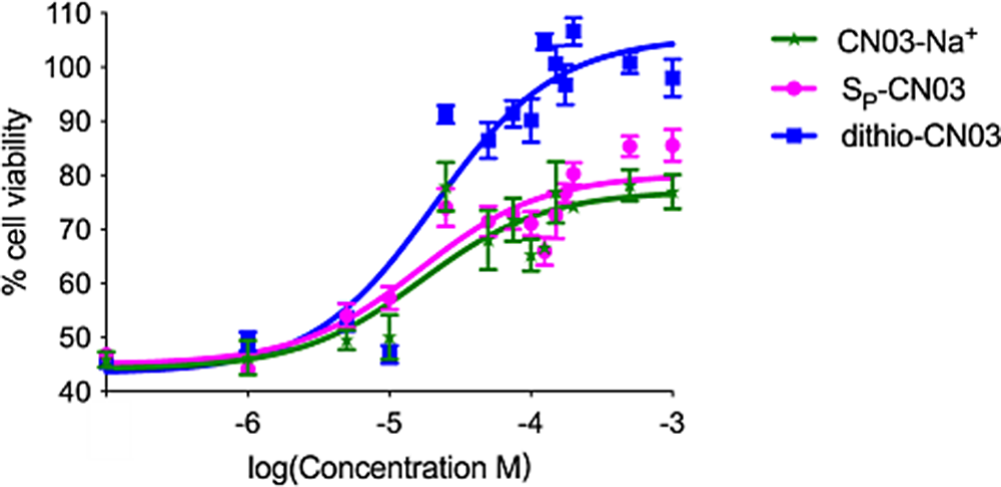
Calculation of the concentration effective in producing 50% of the maximal response of CN03 compounds. Dose–response curves of 661W-A11 cell viability upon stress with 200 μM zaprinast for 48 h and scaling doses (1 μM to 1 mM) of CN03 compounds. EC_50_ was calculated for each compound: 16.9 μM for CN03-Na^+^ (green curve), 16 μM for *S*_P_-CN03 (pink curve), and 20 μM for dithio-CN03 (blue curve).

To quantify the effects of the new CN03 analogues, we calculated the EC_50_ values of the three compounds that showed improved neuroprotective effects. For this analysis, we used the 661W-A11 cells stressed with zaprinast and exposed them to scaling concentrations of either dithio-CN03 or *S*_P_-CN03 or CN03-Na^+^. The effects were evaluated by the cell viability MTT assay (Figure 5). The dose–response curves showed that all three compounds were able to exert a protective effect and exhibited efficacy starting at the concentration of 10 μM. The EC_50_ were very similar for the three compounds: 16.9 μΜ for CN03-Na^+^, 16 μM for *S*_P_-CN03, and 20 μM for dithio-CN03. Nevertheless, CN03-Na^+^ and *S*_P_-CN03 reached up to 70–80% cell viability, while dithio-CN03 could protect rod photoreceptors to almost 100% cell viability at the highest doses (Figure 5). Although dithio-CN03 was considered more hydrophobic, and its entrance in the cells could have been facilitated by this feature, the calculated EC_50_ was nonetheless similar to more hydrophilic analogues. These data suggest that the improved protection of rod photoreceptors by dithio-CN03 can more likely be ascribed to its potentially higher resistance to cleavage by phosphodiesterases^14−16^ or to its ability to attack more and yet unidentified targets in the degenerating photoreceptors.

## Conclusions

In summary, in this study, we presented synthesis, chemicophysical characterization, and effects on photoreceptor cells of a new cGMP analogue, dithio-CN03. Dithio-CN03 owns several improved features such as (i) a reduced hydrosolubility, when compared to the TEA salt of CN03, that can be further lowered to support formulations aimed at sustained and slow release upon administration to the human degenerating retina; (ii) significantly enhanced neuroprotective activity on photoreceptor cells stressed by increased intracellular cGMP. Based on these features we were expecting an improved EC_50_, compared to CN03-Na^+^, that was not observed. These data thus suggest that the modification with two sulfur atoms may have generated a molecule more resistant to phosphodiesterases.^14−16^ Second, we cannot exclude the possibility that dithio-CN03 might attack still uncharacterized targets that contribute to photoreceptor degeneration. While dithio-CN03 showed very promising protective effects on primary cells from *rd1* mutant retinas, in the future it will be interesting to test delivery of dithio-CN03 in vivo in animal models of RP.

## Experimental Section

CN03, CN03-Na^+^, and their precursors were prepared according to the protocols reported in the literature.^11,12^ All compounds tested in vitro (CN03, CN03-Na^+^, *S*_P_-CN03, oxo-CN03, and dithio-CN03) were >95% pure by HPLC analysis.

### Triethylammonium 8-Bromo-β-phenyl-1,N^2^-etheno-2′-triisopropylsilyloxyguanosine-5′-H-phosphonothioate (**2**)

Starting nucleoside **1** (5.00 g, 8.08 mmol) and triethylammonium phosphinate (1.32 g, 0.98 equiv, 7.92 mmol) were dissolved in excess pyridine. The mixture was concentrated under reduced pressure to a volume of 50 mL (0.16 M) and subsequently cooled to 0 °C in an ice bath. Pivaloyl chloride (0.97 g, 1 equiv, 8.08 mmol) and then sulfur (0.52 g, 2 equiv, 16.16 mmol) were added and the ice bath was removed. After stirring for 1 h, the mixture was quenched by addition of 1 M triethylammonium acetate (TEAA) (8.08 mL, 1 equiv, 8.08 mmol) and stirred for 1 h. The mixture was then concentrated under reduced pressure, and the resulting crude oil was partitioned between DCM (500 mL) and 1 M TEAA (100 mL × 2). The organic phase was washed with water (50 mL × 2), dried over Na_2_SO_4_, and evaporated. Residual sulfur in the crude product was precipitated by the addition of MeCN (500 mL) and filtered off. The solvent was swapped with a minimum volume of DCM and applied to a silica gel column. The product eluted from the gel with a mixture of 4:6 DCM:MeCN (with 0.5% v/v triethylamine) and was evaporated to dryness to yield a solid yellow foam. Yield: 26.4% (1.87 g, 93.5% HPLC purity). ^1^H NMR (500 MHz, CDCl_3_): δ 12.82 (br s, 0.5H), 12.5 (br s, 0.5H), 11.19 (br s, 1H), 8.08 (d, *J*_PH_ = 590.1 Hz, 0.5H), 8.04 (d, *J*_PH_ = 585.9 Hz, 0.5H), 7.83 (br s, 1H), 7.78–7.65 (m, 2H), 7.48–7.31 (m, 3H), 5.94 (br s, 1H), 5.79–5.63 (m, 1H), 4.58–4.16 (m, 5H), 3.09 (dq, *J* = 7.1 Hz, *J* = 3.0 Hz, 6H), 1.07 (br t, *J* = 7.2 Hz, 9H), 1.00–0.84 (m, 21H). ^13^C NMR (126 MHz, CDCl_3_): δ 151.2, 150.6, 146.1, 134.7, 129.9, 129.2, 128.0, 125.3, 115.3, 102.7, 89.8, 83.8 (d, *J*_PC_ = 16.0 Hz, 1C), 72.7, 72.5, 71.9, 71.5, 64.8 (d, *J*_PC_ = 4.1 Hz, 1C), 45.8, 17.7, 17.5, 12.0, 8.6. ^31^P NMR (203 MHz, CDCl_3_): δ 56.73 (d, *J*_HP_ = 586.6 Hz, 0.5P), 55.80 (d,^1^*J*_HP_ = 590.1 Hz, 0.5P). MS (M – Et_3_NH^+^) *m*/*z*: 696.1077 calcd for C_27_H_36_BrN_5_O_6_PSSi; found, 696.1133 (ES^–^).

### Triethylammonium 8-Bromo-β-phenyl-1,N^2^-etheno-2′-triisopropylsilyloxyguanosine-3′,5′-cyclicphosphorodithioate (**3**)

Starting H-phosphonothioate **2** (2.98 g, 3.50 mmol) was dissolved in DCM (60 mL, 20 vol). 2,6-Lutidine (1.88 g, 5 equiv, 17.51 mmol) was added to the mixture, followed by pivaloyl chloride (0.55 g, 1.3 equiv, 4.55 mmol). After stirring for 1 h, sulfur (0.17 g, 1.5 equiv, 5.25 mmol) and triethylamine (0.53 g, 1.5 equiv, 5.25 mmol) were added to the mixture. The mixture was stirred for another 1 h and subsequently washed with water (12 mL × 2), and the organic phase was evaporated. The residues were stirred in MeCN, which precipitated sulfur as yellow crystals that were filtered off. The product was chromatographed from a silica gel column with an 8:2 DCM:MeCN mixture (with 0.5% v/v triethylamine) and was evaporated to dryness to yield a yellow oil. Yield: 48% (1.36 g, 99.2% HPLC purity). ^1^H NMR (500 MHz, DMSO-*d*_6_): δ 9.48 (br s, 1H), 7.84 (s, 1H), 7.61–7.55 (m, 2H), 7.46–7.39 (m, 2H), 7.38–7.31 (m, 1H), 6.07–5.99 (m, 1H), 5.93 (s, 1H), 5.13 (d, *J* = 5.0 Hz, 1H), 4.72–4.64 (m, 1H), 4.42–4.26 (m, 2H), 3.34 (q, *J* = 7.3 Hz, 6H), 1.43 (t, *J* = 7.3 Hz, 9H), 1.18–1.02 (m, 21H). ^13^C NMR (126 MHz, DMSO-*d*_6_): δ 151.0, 150.0, 149.4, 145.6, 136.5, 134.4, 129.6, 129.3, 127.4, 125.1, 124.1, 115.6, 103.2, 93.7, 75.8 (d, *J*_PC_ = 6.7 Hz, 1C), 73.7 (d, *J*_PC_ = 9.0 Hz, 1C), 72.0 (d, *J*_PC_ = 5.4 Hz, 1C), 46.5, 18.0, 12.1, 8.8. ^31^P NMR (203 MHz, DMSO-*d*_6_): δ 112.15 (d,^3^*J*_HP_ = 24.7 Hz) (phosphorus splitting observed as a doublet instead of a quartet despite three adjacent ribose protons). MS (M – Et_3_NH^+^) *m*/*z*: 710.0692 calcd for C_27_H_3_4BrN_5_O_5_PS_2_Si; found, 710.0754 (ES^–^).

### Triethylammonium *S*_P_-8-Bromo-β-phenyl-1,N^2^-etheno-2′-triisopropylsilyloxyguanosine-3′,5′-cyclicmonophosphorothioate (**6**)

Starting H-phosphonate **4** (1.03 g, 1.31 mmol) was suspended in excess pyridine. The mixture was concentrated under reduced pressure to a volume of 20 mL (65 mM). Pivaloyl chloride (0.21 g, 1.3 equiv, 1.71 mmol) was added to the mixture, which became a clear solution. The reaction was allowed to stir over two nights before sulfur (63.22 mg, 1.5 equiv, 1.97 mmol) and triethylamine (0.20 g, 1.5 equiv, 1.97 mmol) were added to the mixture. ^31^P NMR of the reaction mixture showed a 2:3 ratio of *R*_P_- and *S*_P_-cyclic phosphorothioate diastereomers. The mixture was stirred for 3 h and subsequently washed with water (10 mL × 2), and the organic phase was evaporated. The residues were stirred in MeCN, which precipitated sulfur as yellow crystals that were filtered off. The product was eluted from a reverse-phase C18 silica gel column with a mixture of 15:85 MeCN:TEAA(aq) 0.5 mM mixture and was evaporated to dryness to yield a yellow oil. Yield: 23% (0.24 g, 85.4% HPLC purity). ^1^H NMR (500 MHz, DMSO-*d*_6_): δ 11.77 (br s, 1H), 7.86 (s, 1H), 7.64–7.57 (m, 2H), 7.48–7.41 (m, 2H), 7.40–7.34 (m, 1H), 6.05–5.97 (m, 1H), 5.94 (s, 1H), 5.20 (d, *J* = 5.0 Hz, 1H), 4.56–4.50 (m, 1H), 4.41–4.31 (m, 1H), 4.29–4.22 (m, 1H), 3.16 (q, *J* = 7.3 Hz, 6H), 1.39 (t, *J* = 7.3 Hz, 9H), 1.17–1.01 (m, 21H). ^13^C NMR (126 MHz, DMSO-*d*_6_): δ 149.01, 148.3, 148.0, 143.8, 132.3, 127.7, 127.4, 125.6, 123.1, 121.1, 115.4, 101.1, 93.3, 92.2, 71.4 (d, *J*_PC_ = 9.8 Hz, 1C), 70.3 (d,^3^*J*_PC_ = 4.8 Hz, 1C), 65.5 (d, *J*_PC_ = 7.2 Hz, 1C), 44.1, 16.0, 10.1, 6.8. ^31^P NMR (203 MHz, DMSO-*d*_6_): δ 54.0 (d, *J*_HP_ = 24.3 Hz) (phosphorus splitting observed as a doublet instead of a quartet, despite three adjacent ribose protons). MS (M – Et_3_NH^+^) *m*/*z*: 694.0920 calcd for C_27_H_34_BrN_5_O_6_PSi; found, 694.0991 (ES^–^).

### Triethylammonium 8-Bromo-β-phenyl-1,N^2^-etheno-2′-triisopropylsilyloxyguanosine-3′,5′-cyclicmonophosphate (**7**)

Starting H-phosphonate **4** (1.03 g, 1.31 mmol) was dissolved in a 1:19 pyridine:DCM mixture (20 mL), followed by addition of pivaloyl chloride (0.21 g, 1.3 equiv, 1.71 mmol). After stirring for 5 min, iodine (0.25 g, 1.5 equiv, 1.97 mmol) was added to the solution, shortly followed by water (0.24 g, 10 equiv, 13.14 mmol). After additional 10 min of stirring, ethanethiol (37 mg, 0.45 equiv, 0.59 mmol) was added, and the mixture was finally concentrated under reduced pressure. The residue was diluted with DCM (20 mL) and washed with water (10 × 3 mL). After a solvent swap to MeCN (20 mL), a white precipitate formed, which was filtered and washed with MeCN. The crude solid was identified as the desired product by diagnostic peaks on ^31^P NMR and HPLC-MS and was telescoped to the following step. ^31^P NMR (203 MHz, DMSO-d6): δ −3.02 (d,^3^*J*_HP_ = 15.9 Hz) (phosphorus splitting observed as a doublet instead of a quartet despite three adjacent ribose protons). MS (M – Et_3_NH^+^) *m*/*z*: 678.1148 calcd for C_27_H_34_BrN_5_O_7_PSi; found, 678.1194 (ES^–^).

### Triethylammonium 8-Bromo-β-phenyl-1,N^2^-ethenoguanosine-3′,5′-cyclicmonophosphorodithioate (dithio-CN03)

Triethylamine trishydrofluoride (1.7 mL, 2 vol) was charged to a solution of crude phosphorodithioate **3** (850 mg, 1.04 mmol) in THF (3.4 mL, 4 vol). A precipitate formed over three nights, which was filtered out and washed with one cake volume of THF. The solid was dried under a vacuum at 40 °C, affording the target dithio-CN03 as a white powder. Yield: 69% (470 mg, 99.6% HPLC purity). ^1^H NMR (500 MHz, DMSO-*d*_6_): δ 8.20 (s, 1H), 7.93–7.87 (m, 2H), 7.54–7.46 (m, 2H), 7.45–7.39 (m, 1H), 5.76 (d, *J* = 1.7 Hz, 1H), 5.18–5.11 (m, 1H), 5.11–5.05 (m, 1H), 4.40–4.30 (m, 1H), 4.20–4.10 (m, 1H), 3.95–3.89 (m, 1H), 3.09 (q, *J* = 7.3 Hz, 6H), 1.17 (t, *J* = 7.3 Hz, 9H). ^13^C NMR (126 MHz, DMSO-*d*_6_): δ 150.4, 150.1, 145.7, 129.7, 129.1, 129.0, 127.5, 125.1, 115.9, 103.3, 92.9, 75.2 (d, *J*_PC_ = 6.4 Hz, 1C), 71.5 (d, *J*_PC_ = 5.5 Hz, 1C), 69.5 (d, *J*_PC_ = 8.1 Hz, 1C), 65.6 (d, *J*_PC_ = 9.2 Hz, 1C), 45.8, 8.5. ^31^P NMR (203 MHz, DMSO-*d*_6_): δ 112.6 (d,^3^*J*_HP_ = 23.5 Hz) (phosphorus splitting observed as a doublet instead of a quartet despite three adjacent ribose protons). MS (M – Et_3_NH^+^) *m*/*z*: 553.9357 calcd for C_18_H_14_BrN_5_O_5_PS_2_; found, 553.9364 (ES^–^).

### Triethylammonium *S*_P_-8-Bromo-β-phenyl-1,N^2^-ethenoguanosine-3′,5′-cyclicmonophosphorothioate (*S*_P_-CN03)

Triethylamine trishydrofluoride (1.26 mL, 2 vol) was charged to a solution of crude phosphorothioate **6** (630 mg, 0.79 mmol) in THF (2.52 mL, 4 vol). A precipitate formed over four nights which was too fine to filter out, and the mixture was concentrated under reduced pressure. Cooling recrystallization of the residue from EtOH (12.6 mL, 20 vol), followed by filtration and washing with one cake volume of the same, afforded the target *S*_P_-CN03 as a white powder. Yield: 99% (500 mg, >99.9% HPLC purity). ^1^H NMR (500 MHz, DMSO-*d*_6_): δ 8.20 (s, 1H), 7.95–7.87 (m, 2H), 7.53–7.46 (m, 2H), 7.45–7.38 (m, 1H), 5.77 (d, *J* = 1.7 Hz, 1H), 5.09–5.05 (m, 1H), 5.04–4.95 (m, 1H), 4.23–4.11 (m, 2H), 3.95–3.87 (m, 2H), 3.09 (q, *J* = 7.3 Hz, 6H), 1.18 (t, *J* = 7.3 Hz, 9H). ^13^C NMR (126 MHz, DMSO-*d*_6_): δ 150.5, 150.0, 145.7, 129.5, 129.1, 127.5, 125.2, 122.5, 115.9, 103.5, 93.4, 76.5 (d, *J*_PC_ = 5.5 Hz, 1C), 71.7 (d, *J*_PC_ = 5.2 Hz, 1C), 69.3 (d, *J*_PC_ = 9.1 Hz, 1C), 65.6 (d, *J*_PC_ = 6.7 Hz, 1C), 45.7, 8.5. ^31^P NMR (203 MHz, DMSO-*d*_6_): δ 50.7 (d,^3^*J*_HP_ = 25.7 Hz) (phosphorus splitting observed as a doublet instead of a quartet despite three adjacent ribose protons). MS (M – Et_3_NH^+^) *m*/*z*: 537.9586 calcd for C_18_H_14_BrN_5_O_6_PS; found, 537.9584 (ES^–^).

### Triethylammonium 8-Bromo-β-phenyl-1,N^2^-ethenoguanosine-3′,5′-cyclicmonophosphate (oxo-CN03)

Triethylamine trishydrofluoride (3.2 mL, 2 vol) was charged to a solution of crude phosphate **7** (1.6 g, 2.05 mmol) in MeOH (6.4 mL, 4 vol). A precipitate formed over four nights which was filtered and washed with MeOH. The solids were resuspended in EtOH (32.0 mL, 20 vol), followed by filtration and washing with one cake volume of the same, affording the target oxo-CN03 as a white powder. Yield: 56% (710 mg, 99.0% HPLC purity). ^1^H NMR (500 MHz, DMSO-*d*_6_): δ 8.11 (s, 1H), 7.86–7.80 (m, 2H), 7.53–7.46 (m, 2H), 7.44–7.39 (m, 1H), 5.77 (s, 1H), 5.62–5.55 (m, 1H), 4.68 (d, *J* = 5.4 Hz, 1H), 4.20–4.11 (m, 1H), 4.10–4.04 (m, 1H), 3.99–3.92 (m, 1H), 3.06 (q, *J* = 7.3 Hz, 6H), 1.16 (t, *J* = 7.3 Hz, 9H). ^13^C NMR (126 MHz, DMSO-*d*_6_): δ 150.3, 150.1, 145.6, 129.8, 129.3, 129.1, 127.5, 125.0, 122.8, 115.9, 103.1, 93.7, 76.3 (d, *J*_PC_ = 4.3 Hz, 1C), 72.5, 71.3 (d, *J*_PC_ = 8.3 Hz, 1C), 65.9 (d, *J*_PC_ = 4.1 Hz, 1C), 45.8, 8.4. ^31^P NMR (203 MHz, DMSO-*d*_*6*_): δ −4.16 (d,^3^*J*_HP_ = 20.9 Hz) (phosphorus splitting observed as a doublet instead of a quartet despite three adjacent ribose protons). MS (M – Et_3_NH^+^) *m*/*z*: 521.9814 calcd for C_18_H_14_BrN_5_O_7_P; found, 521.9802 (ES^–^).

### X-ray Powder Diffraction (XRPD)

XRPD analyses were performed at 20 °C on a PANalytical X′Pert PRO instrument, equipped with a Cu X-ray tube and a PIXcel detector. Automatic divergence and antiscatter slits were used, together with 0.02 rad Soller slits and a Ni filter. Solid samples were analyzed on cut silicon zero background holders (ZBH). Slurry samples were dripped on porous alumina substrates, which produce peaks at 25.6, 35.0, and 37.7° 2θ. The randomness of the analysis was increased by spinning them during the analysis. Samples were analyzed between 2 and 40° in 2θ over 17 min.

### Thermal Analysis

DSC analyses were carried out on a Mettler DSC822e, equipped with a Thermo Haake EK45/MT cooler. The samples were weighed into a 40 μL Al-cup, which was then closed with a pierced lid. The sample was scanned from 25 to 300 °C, with a scan rate of 10 °C/min.

TG was performed on a Mettler TGA/SDTA 851e, equipped with a Haake C50P cooler. Alternatively, thermogravimetric and DSC analyses were carried out simultaneously in a Mettler Toledo TGA/DSC 3+. The samples were weighed into a 100 μL Al-cup, and this was flushed with dry nitrogen gas during the analysis. The sample was scanned from 25 to 300 °C, with a scan rate of 10 °C/min.

Hot-stage optical microscopy was performed on a Mettler Toledo HS82 hot-stage system with an internal furnace and an HS1 Hot Stage control unit. The sample was heated from 25 to 300 °C at a rate of 10 K/min and monitored under an Olympus BX51 light-polarizing microscope coupled with a PixeLINK PL-A662 digital camera.

### NMR

All NMR spectra were recorded on a Bruker AV 500 MHz (500.13 MHz in ^1^H, 125.76 MHz in ^13^C, and 202.47 MHz in ^31^P) spectrometer, using the given deuterated solvent as an internal standard. In some cases, maleic acid was used as an internal standard for assay determination. Chemical shifts (δ scale) are reported in parts per million (ppm), and coupling constants (*J* values) are reported in Hertz (Hz).

### Solubility Studies

The solubility of dithio-CN03 was measured at room temperature and in quadruplicate by phase solubility techniques. Saturated mixtures of the compound were prepared by adding excess starting material to 4 mL vials containing 2 mL of deionized water. They were allowed to equilibrate for 24 h using mild agitation before recording the pH. The clear solution was separated from the solids by filtration with a Cytiva Whatman Mini-Uniprep polypropylene 0.45 μm filter device and diluted to an appropriate analytical concentration where needed. The sample was then analyzed by HPLC, and the solubility was determined by integrating the area of the peak of interest against a standard curve. The solids were also analyzed by XRPD to record the crystal modification obtained.

A temperature-dependent solubility curve was performed using a TTP LabTech SolMate Solubility Block coupled with a Haake C25 temperature-control system. Ten vials were prepared with increasing amounts of dithio-CN03 (0.5–8 mg) in 1.2 mL of deionized water. The vials were placed in the instrument with magnetic stirring and equilibrated at 20 °C for 24 h. The temperature was then increased by steps of 45 min of 2 °C, up to 72 °C, followed by a final step at 75 °C. The temperature was then lowered to 20 °C, and the cycle was repeated twice. The turbidity of clear solutions was calibrated at 1000 counts, while suspensions were calibrated at 4000 counts. Samples were considered dissolved when the average turbidity dropped below 1100 and remained constant.

### High-Performance Liquid Chromatography (HPLC)-Mass Spectrometry

HPLC analysis was carried out on a Thermo Fisher Scientific Vanquish VH-10-A UHPLC equipped with a VH-D10-A photo diode array detector and an ISQ EM Single Quadrupole Mass Spectrometer. UV detection was taken at 254 or 210 nm, and mass detection was between 100–1000 in both negative and positive mode. An Acquity Premiere BEH C18 column (50 × 2 mm, 1.7 μm particle size) was used with a 0.4 mL/min flow rate at 40 °C and a linear gradient of 2–100% of buffer B in buffer A at 40 °C. The buffers were: 5 mM ammonium acetate (A); and MeCN (B).

For high-resolution mass spectra (HRMS) an Acquity UPLC coupled with a Xevo G2-XS QT Mass Spectrometer was used instead (in ES^–^ ionization mode). The column was an ACQUITY Premier Oligonucleotide C18 Column (130 Å, 2.1 × 50 mm, 1.7 μm particle size), the gradient was 5–100% of buffer B in buffer A over 5 min at 40 °C. The buffers were: 5 mM ammonium acetate (A); and 80% MeCN in A (B).

### Cell Culture

661W-A11, a cell line derived from 661W cells^30^ with rod photoreceptor characteristics,^24^ was cultured in 1 mg/mL Glucose DMEM (Dulbecco’s Modified Eagle Medium, Gibco) supplemented with 10% FBS (Fetal Bovine Serum, Gibco), 2 mM glutamine, 100 U/mL penicillin, and 100 μg/mL streptomycin (Thermo Fisher Scientific) in an incubator at 5% CO_2_ and 37 °C. Every 3 days cells were split and subcultured.

### Toxicity Assay

661W-A11 cells were seeded in 96-well plates at a density of 6000 cells/well, and the following day they were treated with scaling concentrations of either CN03-Na^+^, CN03, *S*_P_-CN03, oxo-CN03, or dithio-CN03 for 48 h. Control groups were treated with an equal volume of vehicle H_2_O or DMSO in the culture medium. Cell viability was assessed with MTT (3-(4,5-dimethylthiazol-2-yl)-2,5-diphenyl tetrazolium bromide, Sigma). For the MTT assay, the supernatant was removed, and the purple formazan crystals were dissolved in 100 μL of isopropanol. The crystals were shaken for 10 min to dissolve and the optical density (OD) was measured at a wavelength of 570 nm.

### Cell Treatment with Compounds

661W-A11 cells were seeded in 96-well plates at a density of 6000 cells/well or in 24-well plates at a density of 20,000 cells/well. Twenty-four h after seeding, cells were pretreated with 50 μM of either CN03-Na^+^ or CN03 or *S*_P_-CN03 or oxo-CN03 or dithio-CN03 for 2 h, followed by 200 μM zaprinast treatment for 48 h in the presence of the compounds. Control groups were treated with an equal volume of vehicle, H_2_O or DMSO. While the zaprinast *K*_i_ value on PDE6 purified enzyme is 30 nM,^31^ we chose the concentration of 200 μM zaprinast for 48 h based on our previous study, showing that this concentration was adequate to increase intracellular cGMP in 661W-A11 cells.^24^

### Cell Viability Assay

661W-A11 cells were cultured on 96-well plates at a density of 6000 cells/well. After compound treatments, the culture medium was replaced with medium containing 1 mg/mL of MTT (Sigma) and cells were cultured for 90 min at 37 °C. Viability was assessed by MTT assay (see above for detailed procedure). OD was measured at 570 nm. Effects of different amounts of vehicles on cell viability are presented in Figure S39.

### Cell Death Assay

661W-A11 cells were seeded in 24-well plates at a density of 20,000 cells/well. After treatments, cells were fixed with 2% paraformaldehyde for 10 min and permeabilized with 1 M Na^+^ citrate and 0.1% Triton X-100 on ice for 2 min. Cells were incubated with TUNEL solution “In situ Cell Death Detection Kit” TMR Red (Roche) for 30 min at 37 °C, and cell nuclei were stained with 0.1 μg/mL 4′,6-diamidin-2-fenilindolo (DAPI). Samples were mounted with Mowiol 4–88 (Sigma), and cells were observed with a Zeiss Axio Imager A2 fluorescence microscope. The quantification of percentage of dead cells was based on the ratio of TUNEL-labeled cells on the total number of cells (DAPI-labeled).

### Primary Retinal Cell Culture

*rd1* mutant mice were purchased from Charles River Italy. The protocol was approved by the Ethical Committee of the University of Modena and Reggio Emilia and by the Italian Ministero della Salute (150/2021-PR). Primary neurospheres were derived from the eyes of *rd1* mutant mice, as previously reported.^32^ Retinal stem cells in neurospheres were differentiated into photoreceptors, as previously reported.^10^*rd1* mutant photoreceptor cells underwent spontaneous cell death after differentiation for 11 days. At day 10, 50 μM of either CN03-Na^+^ or *S*_P_-CN03 or dithio-CN03 were added to the cells in culture and cell death was assessed 1 day later (day 11 of differentiation) by TUNEL assay (see above for detailed procedure).

### Dose–Response Curves

661W-A11 cells were seeded in 96-well plates at a density of 6000 cells/well, and the following day they were pretreated with scaling concentrations of either CN03-Na^+^ or *S*_P_-CN03 or dithio-CN03 for 2 h and then stressed with zaprinast 200 μM in the presence of the compounds for 48 h. Cell viability was assessed by MTT assay (see above for detailed procedure). OD was measured at 570 nm. Data were plotted and EC_50_ calculated using GraphPad Prism version 7.

### Statistical Analysis

Data from each experiment, obtained from at least three biological replicates, are presented as means ± SD and means ± SEM for the dose–response curve. Statistical analysis was performed with GraphPad Prism version 7 (GraphPad software, San Diego, CA, USA), each data set was analyzed by one-way and two-way analysis of variance (ANOVA) and Student’s *t* test.

## Supporting Information Available

NMR spectra, HPLC-UV/-MS traces of reported compounds, X-ray powder diffractograms and aqueous solubility measurements for dithio-CN03, and viability assay of vehicles used to dissolve compounds (PDF)
SMILES strings of the reported compounds (CSV)

## Abbreviations

cGMP: cyclic guanosine monophosphate
CNGC: cyclic nucleotide-gated channels
DAPI: 4′,6-diamidin-2-fenilindolo
DMEM: Dulbecco’s Modified Eagle Medium
DMSO: dimethyl sulfoxide
DSC: differential scanning calorimetry
FBS: fetal bovine serum
HPLC: high-performance liquid chromatography
HRMS: high-resolution mass spectra
MeCN: acetonitrile
MeTHF: methyl tetrahydrofuran
MTT: 3-(4,5-dimethylthiazol-2-yl)-2,5-diphenyl tetrazolium bromide
NMR: nuclear magnetic resonance
PKG: cGMP-dependent protein kinase G
Pv-Cl: pivaloyl chloride
RP: retinitis pigmentosa
TEA: triethylammonium
TEAH: H-phosphonothioates
TG: thermogravimetry
THF: tetrahydrofuran
TUNEL: terminal deoxynucleotidyl transferase dUTP nick-end labeling
XRPD: X-ray powder diffraction
