# Supplementary material for: New Improved cGMP Analogues to Target Rod Photoreceptor Degeneration
Source: J Med Chem. 2024 Apr 30;67(10):8396–405. doi: 10.1021/acs.jmedchem.4c00586 (PMC11129186; doi:10.1021/acs.jmedchem.4c00586)
Supplement: Supplementary file 1 — jm4c00586_si_001.pdf [file jm4c00586_si_001.pdf]

**Supporting Information**

**New improved cGMP analogues to target rod photoreceptor degeneration.**

Oswaldo Pérez†‡*, Agnese Stanzani§, Li Huang§, Nicolaas Schipper†, Thorsteinn Loftsson‡, Martin Bollmark†, Valeria Marigo§*

†Chemical Processes and Pharmaceutical Development Research Institutes of Sweden, Forskargatan 20 J, 15136 Södertälje (Sweden).

‡Faculty of Pharmaceutical Sciences, University of Iceland, Hofsvallagata 53, 107 Reykjavik, (Iceland).

§Department of Life Sciences, University of Modena and Reggio Emilia, via Campi 287, 41125 Modena (Italy)

* Coprresponding authors: Oswaldo Pérez: oswaldo.perez@ri.se; Valeria Marigo: valeria.marigo@unimore.it

## Table of Contents

Triethylammonium $R_P$-8-Bromo-β-phenyl-1,$N^2$-ethenoguanosine-3′,5′-cyclicmonophosphorothioate (CN03)....4

Figure S1: HPLC-UV-MS....4

Sodium $R_P$-8-Bromo-β-phenyl-1,$N^2$-ethenoguanosine-3′,5′-cyclicmonophosphorothioate (CN03-$Na^+$)....5

Figure S2: HPLC-UV-MS....5

Triethylammonium 8-Bromo-β-phenyl-1,$N^2$-etheno-2′-triisopropylsilyloxyguanosine-5′-*H*-phosphonothioate (2)....6

Figure S3: HPLC-UV-MS....6

Figure S4: $^1$H-NMR ($CDCl_3$, 500 MHz)....7

Figure S5: $^{13}$C-NMR ($CD_3Cl$, 126 MHz)....8

Figure S6: $^{31}$P-NMR ($CDCl_3$, 203 MHz)....9

Figure S7: $^{31}$P{$^1$H}-NMR ($CDCl_3$, 203 MHz)....10

Triethylammonium 8-Bromo-β-phenyl-1,$N^2$-etheno-2′-triisopropylsilyloxyguanosine-3′,5′-cyclicmonophosphorodithioate (3)....11

Figure S8: HPLC-UV....11

Figure S9: $^1$H-NMR ($CDCl_3$, 500 MHz)....12

Figure S10: $^{13}$C-NMR ($CD_3Cl$, 126 MHz)....13

Figure S11: $^{31}$P-NMR ($CDCl_3$, 203 MHz)....14

Figure S12: $^{31}$P{$^1$H}-NMR ($CDCl_3$, 203 MHz)....15

Triethylammonium $S_P$-8-Bromo-β-phenyl-1,$N^2$-etheno-2′-triisopropylsilyloxyguanosine-3′,5′-cyclicmonophosphorothioate (6)....16

Figure S13: HPLC-UV....16

Figure S14: $^1$H-NMR ($CDCl_3$, 500 MHz)....17

Figure S15: $^{13}$C-NMR ($CD_3Cl$, 126 MHz)....18

Figure S16: $^{31}$P-NMR ($CDCl_3$, 203 MHz)....19

Figure S17: $^{31}$P{$^1$H}-NMR ($CDCl_3$, 203 MHz)....20

Triethylammonium 8-Bromo-β-phenyl-1,$N^2$-etheno-2′-triisopropylsilyloxyguanosine-3′,5′-cyclicmonophosphate (7)....21

Figure S18: HPLC-UV-MS....21

Figure S19: $^{31}$P-NMR ($CDCl_3$, 203 MHz)....22

Figure S20: $^{31}$P{$^1$H}-NMR ($CDCl_3$, 203 MHz)....23

Triethylammonium 8-Bromo-β-phenyl-1,$N^2$-ethenoguanosine-3′,5′-cyclicmonophosphorodithioate (Dithio-CN03)....24

Figure S21: HPLC-UV....24

Figure S22: XRPD ........ 25
Figure S23: $^{1}$H-NMR ($(CD_3)_2SO$, 500 MHz) ........ 26
Figure S24: $^{1}$H-NMR ($(CD_3)_2SO$, 500 MHz) ........ 27
Figure S25: $^{13}$C-NMR ($(CD_3)_2SO$, 126 MHz) ........ 28
Figure S26: $^{31}$P-NMR ($(CD_3)_2SO$, 203 MHz) ........ 29
Figure S27: $^{31}$P{$^{1}$H}-NMR ($(CD_3)_2SO$, 203 MHz) ........ 30
Triethylammonium $S_P$-8-Bromo-β-phenyl-1,$N^2$-ethenoguanosine-3′,5′-cyclicmonophosphorothioate ($S_P$-CN03) ........ 31
Figure S28: HPLC-UV ........ 31
Figure S29: $^{1}$H-NMR ($(CD_3)_2SO$, 500 MHz) ........ 32
Figure S30: $^{13}$C-NMR ($(CD_3)_2SO$, 126 MHz) ........ 33
Figure S31: $^{31}$P-NMR ($(CD_3)_2SO$, 203 MHz) ........ 34
Figure S32: $^{31}$P{$^{1}$H}-NMR ($(CD_3)_2SO$, 203 MHz) ........ 35
Triethylammonium 8-Bromo-β-phenyl-1,$N^2$-ethenoguanosine-3′,5′-cyclicmonophosphate (Oxo-CN03) ........ 36
Figure S33: HPLC-UV ........ 36
Figure S34: $^{1}$H-NMR ($(CD_3)_2SO$, 500 MHz) ........ 37
Figure S35: $^{13}$C-NMR ($(CD_3)_2SO$, 126 MHz) ........ 38
Figure S36: $^{31}$P-NMR ($(CD_3)_2SO$, 203 MHz) ........ 39
Figure S37: $^{31}$P{$^{1}$H}-NMR ($(CD_3)_2SO$, 203 MHz) ........ 40
Solubility data for dithio-CN03 ........ 41
Table S1: Aqueous solubility at room temperature ........ 41
Figure S38: Temperature vs solubility curve. ........ 41
Effects of vehicles on cell viability ........ 42
Figure S39: Dose response on 661W-A11 of $H_2O$ and DMSO. ........ 42
References ........ 43

## Triethylammonium $R_P$-8-Bromo-β-phenyl-1,$N^2$-ethenoguanosine-3′,5′-cyclicmonophosphorothioate (CN03)

Preparation and characterization of this compound and its precursors is found in work previously published by some of the authors [1].

**Figure S1: HPLC-UV-MS**

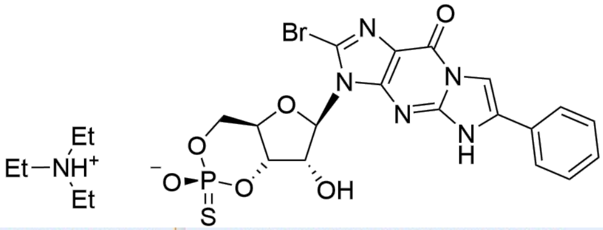

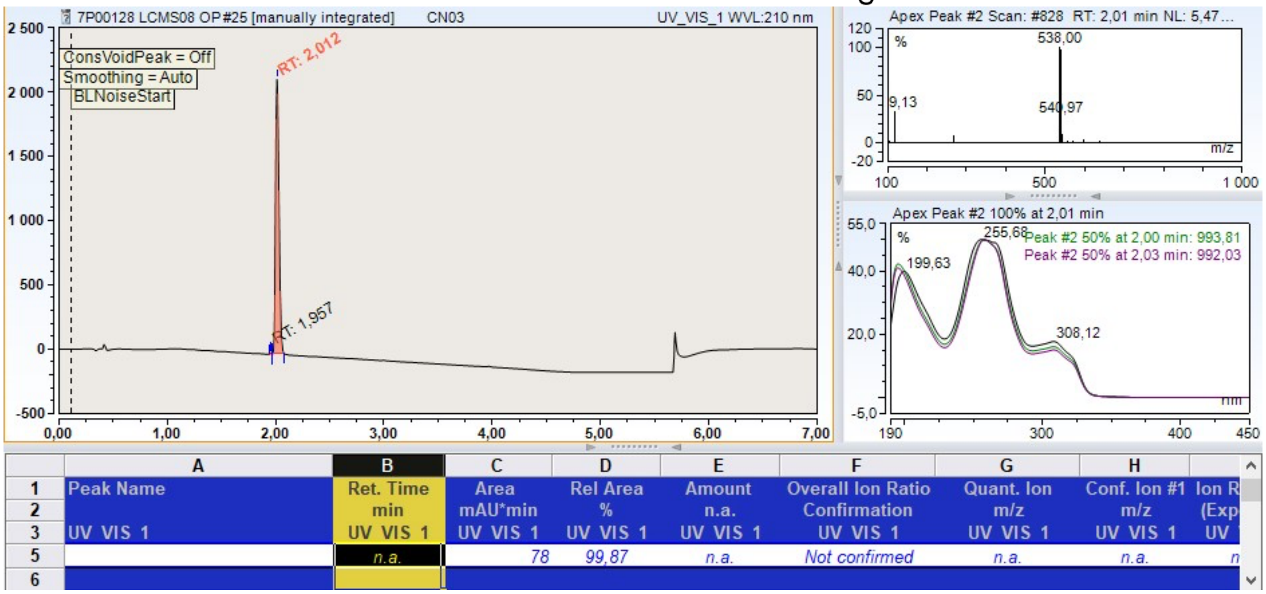

## Sodium $R_{\mathrm{P}}$-8-Bromo-β-phenyl-1,$N^2$-ethenoguanosine-3′,5′-cyclicmonophosphorothioate (CN03-$Na^+$)

Preparation and characterization of this compound is found in work previously published by some of the authors [2].

**Figure S2: HPLC-UV-MS**

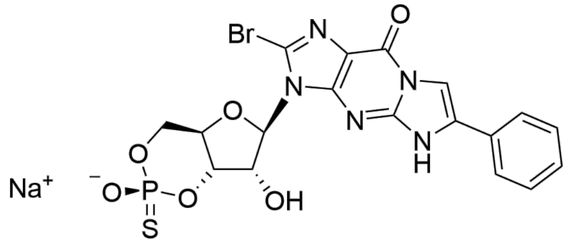


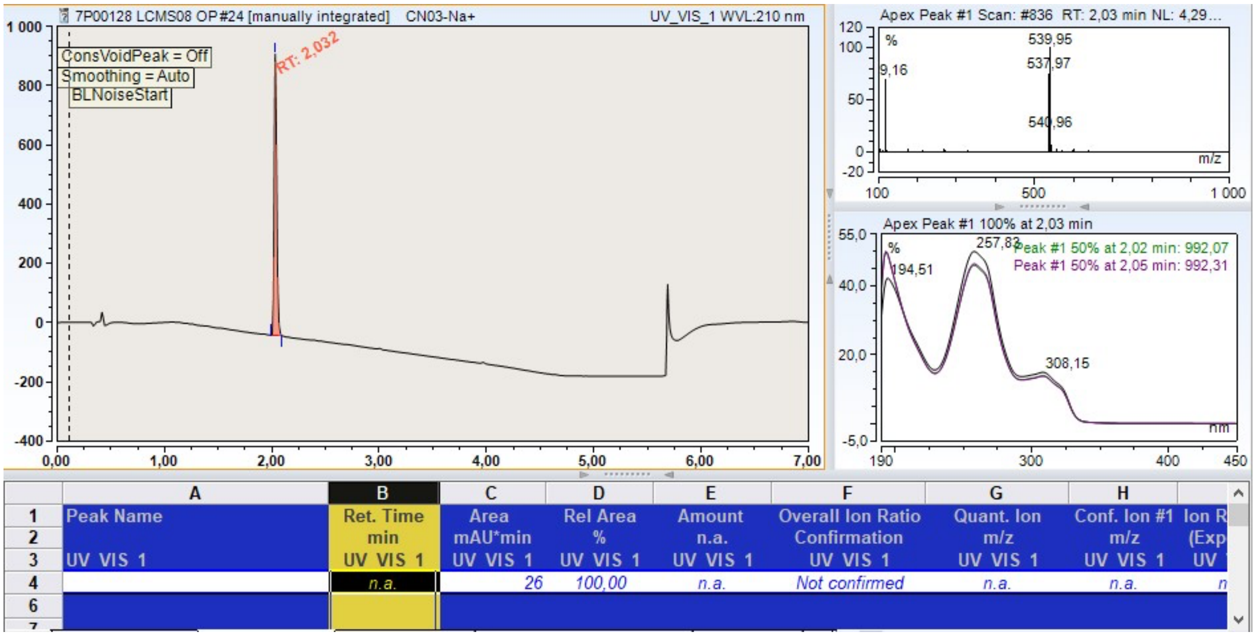

## Triethylammonium 8-Bromo-β-phenyl-1,$N^2$-etheno-2′-triisopropylsilyloxyguanosine-5′-*H*-phosphonothioate (2)

**Figure S3: HPLC-UV-MS**

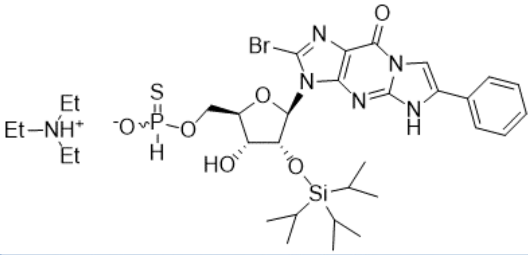

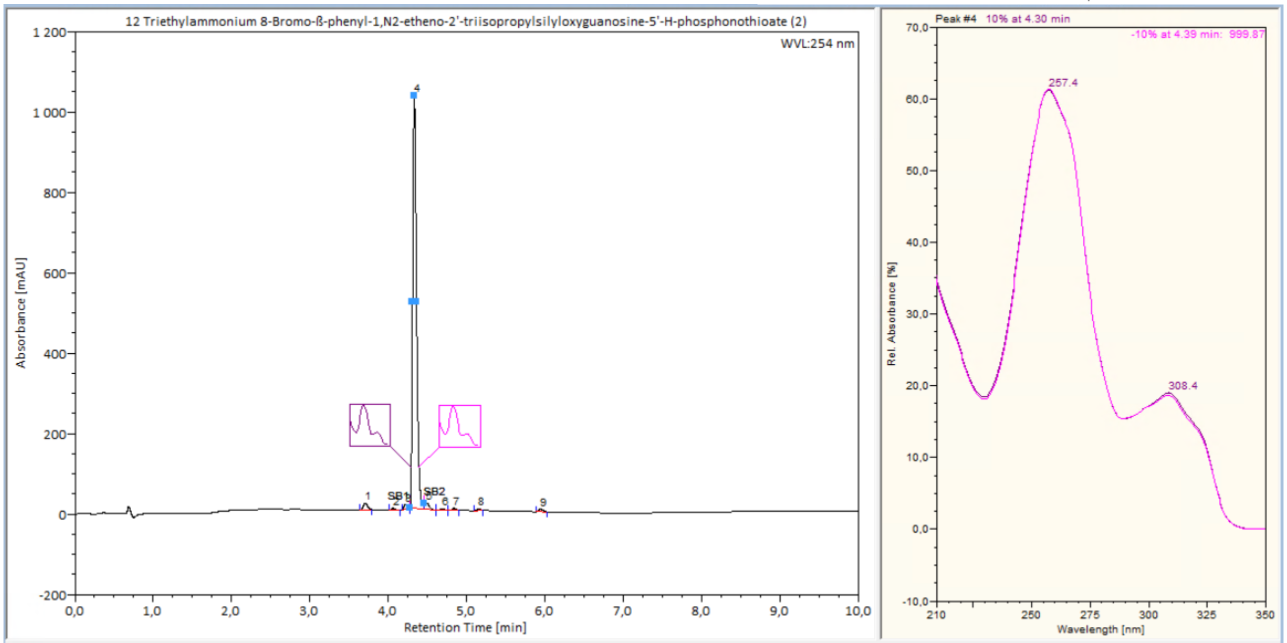


| A | B | C | D | E | F | G | H | I | J | K |
|---|---|---|---|---|---|---|---|---|---|---|
| No. | Peakname | Ret.Time min | RRT % | Area mAU*min | Rel.Area(ISTD) % | Type | Height mAU | Rel.Area % | Resolution | Calc Rel Amount unit |
| 1 | n.a. | 3,708 | n.a. | 1,0016 | n.a. | BMB* | 16,689 | 1,61 | 3,90 | n.a. |
| 2 | n.a. | 4,066 | n.a. | 0,2261 | n.a. | BMB* | 4,227 | 0,36 | 1,86 | n.a. |
| 3 | n.a. | 4,220 | n.a. | 0,5185 | n.a. | BMB* | 10,839 | 0,83 | 1,37 | n.a. |
| 4 | n.a. | 4,334 | n.a. | 58,0374 | n.a. | BM * | 1027,339 | 93,47 | n.a. | n.a. |
| 5 | n.a. | 4,491 | n.a. | 1,1630 | n.a. | Mb* | 15,891 | 1,87 | n.a. | n.a. |
| 6 | n.a. | 4,701 | n.a. | 0,2089 | n.a. | bMb* | 3,085 | 0,34 | 1,34 | n.a. |
| 7 | n.a. | 4,839 | n.a. | 0,2932 | n.a. | bMB* | 5,449 | 0,47 | 3,50 | n.a. |
| 8 | n.a. | 5,155 | n.a. | 0,2179 | n.a. | BMB* | 4,082 | 0,35 | 7,78 | n.a. |
| 9 | n.a. | 5,945 | n.a. | 0,4265 | n.a. | BMB* | 6,085 | 0,69 | n.a. | n.a. |
| Total: | | | | 62,0932 | 0,0000 | | 1093,687 | 100,00 | | |

**Figure S4: $^1$H-NMR ($CDCl_3$, 500 MHz)**

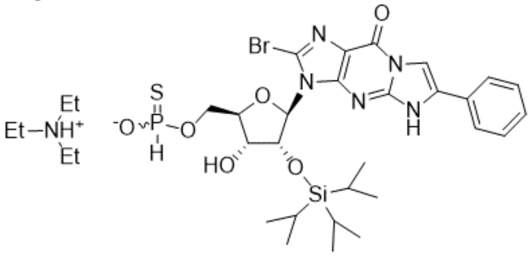

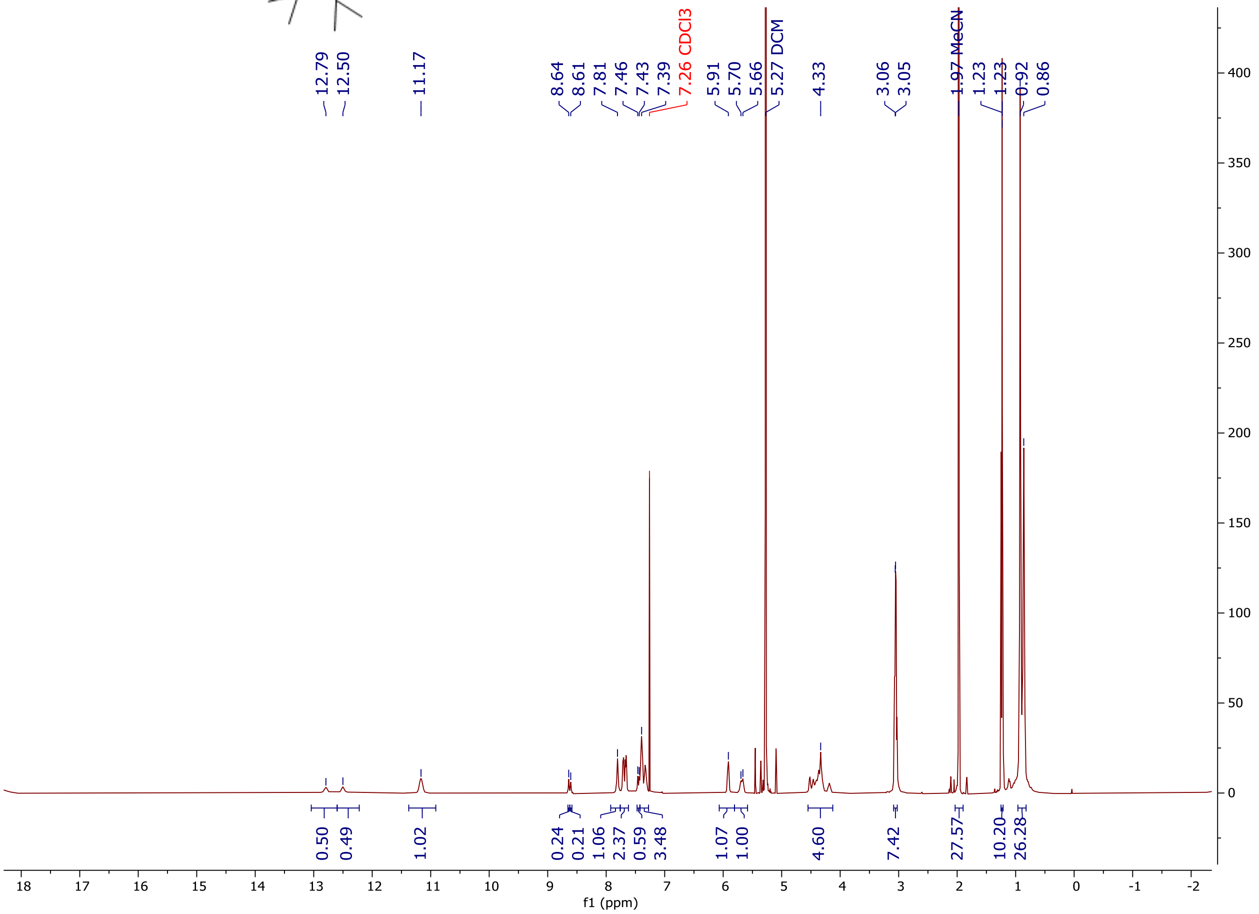

**Figure S5: $^{13}$C-NMR ($CD_3Cl$, 126 MHz)**

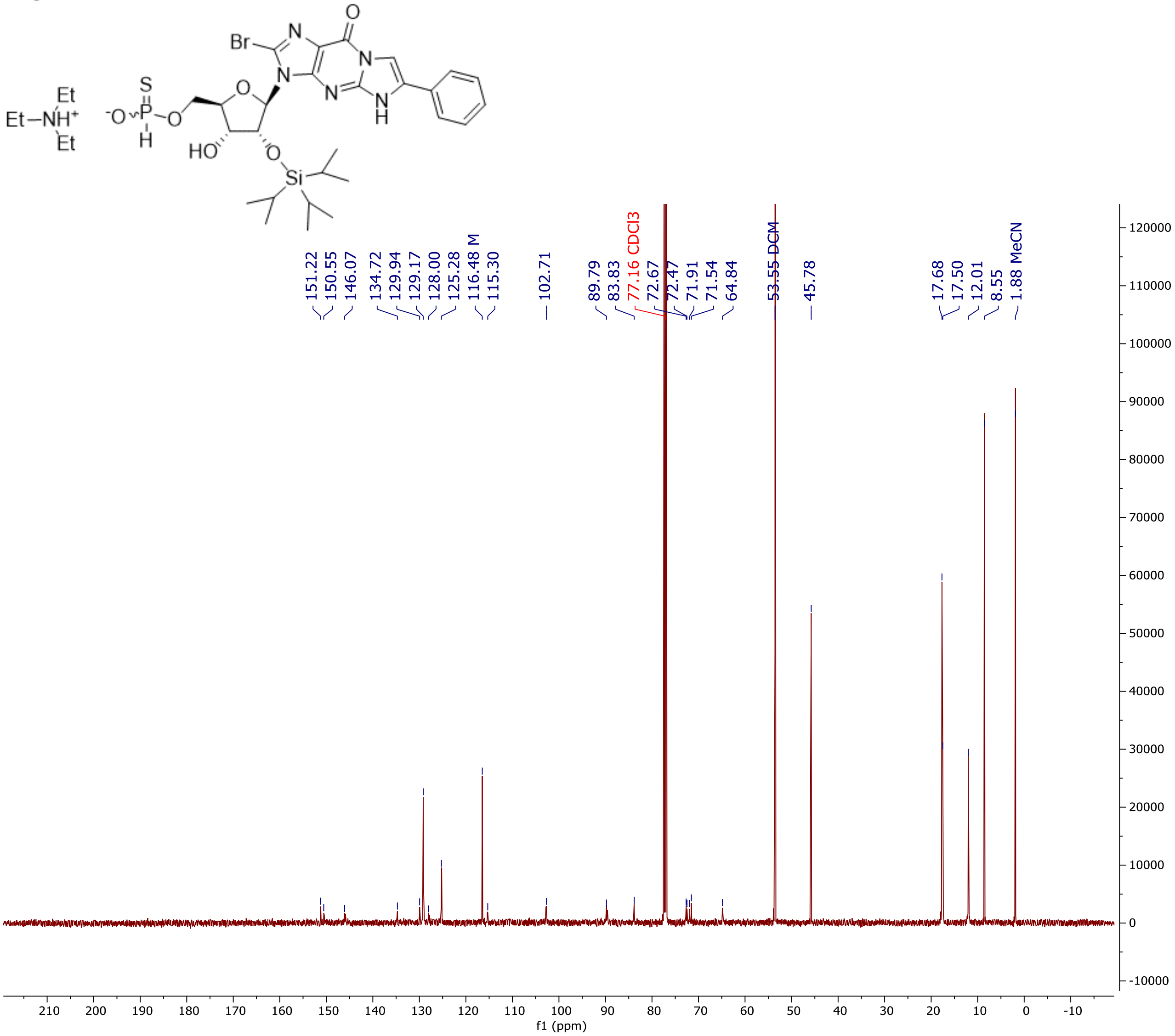

**Figure S6: $^{31}$P-NMR ($CDCl_3$, 203 MHz)**

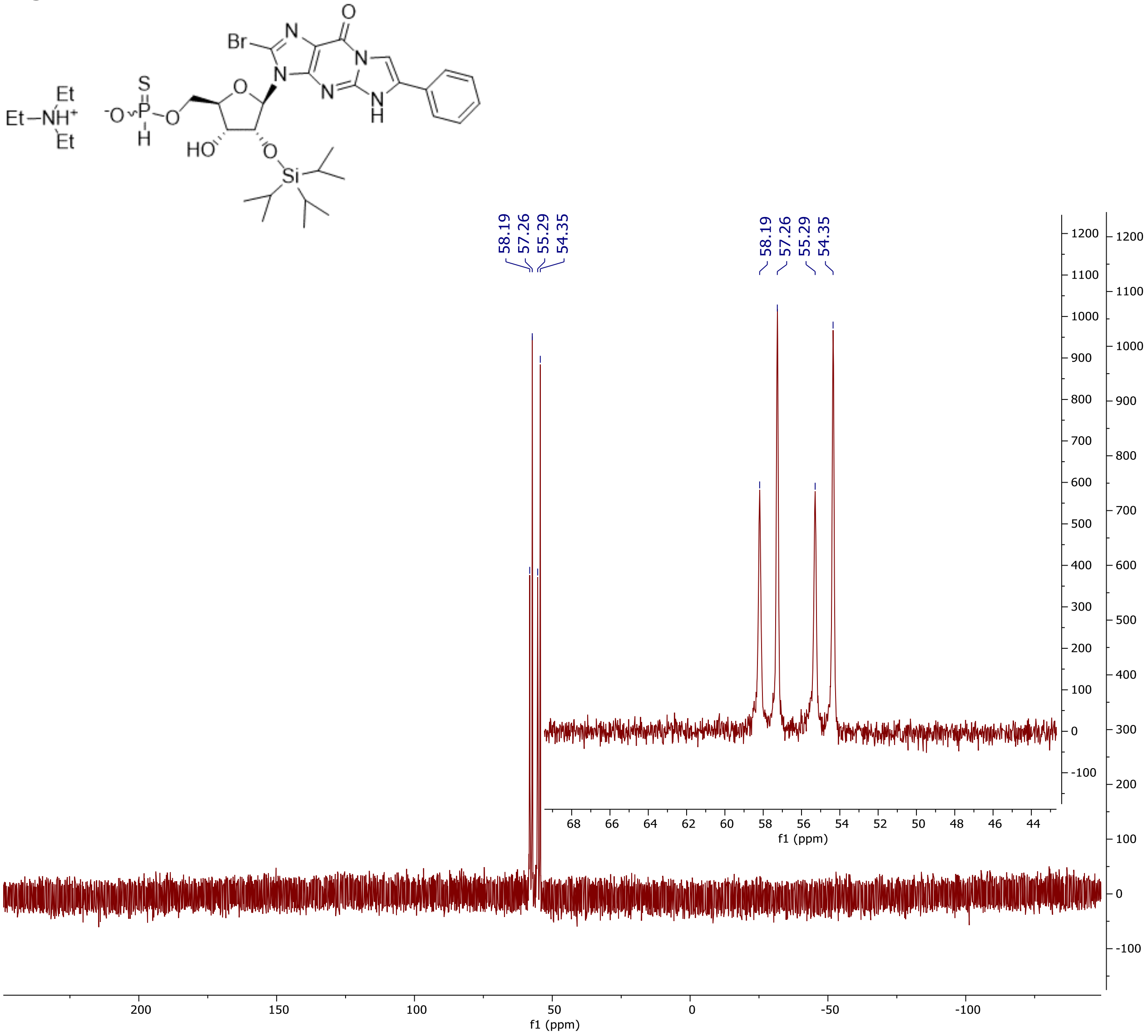

**Figure S7: $^{31}P\{^{1}H\}$-NMR ($CDCl_3$, 203 MHz)**

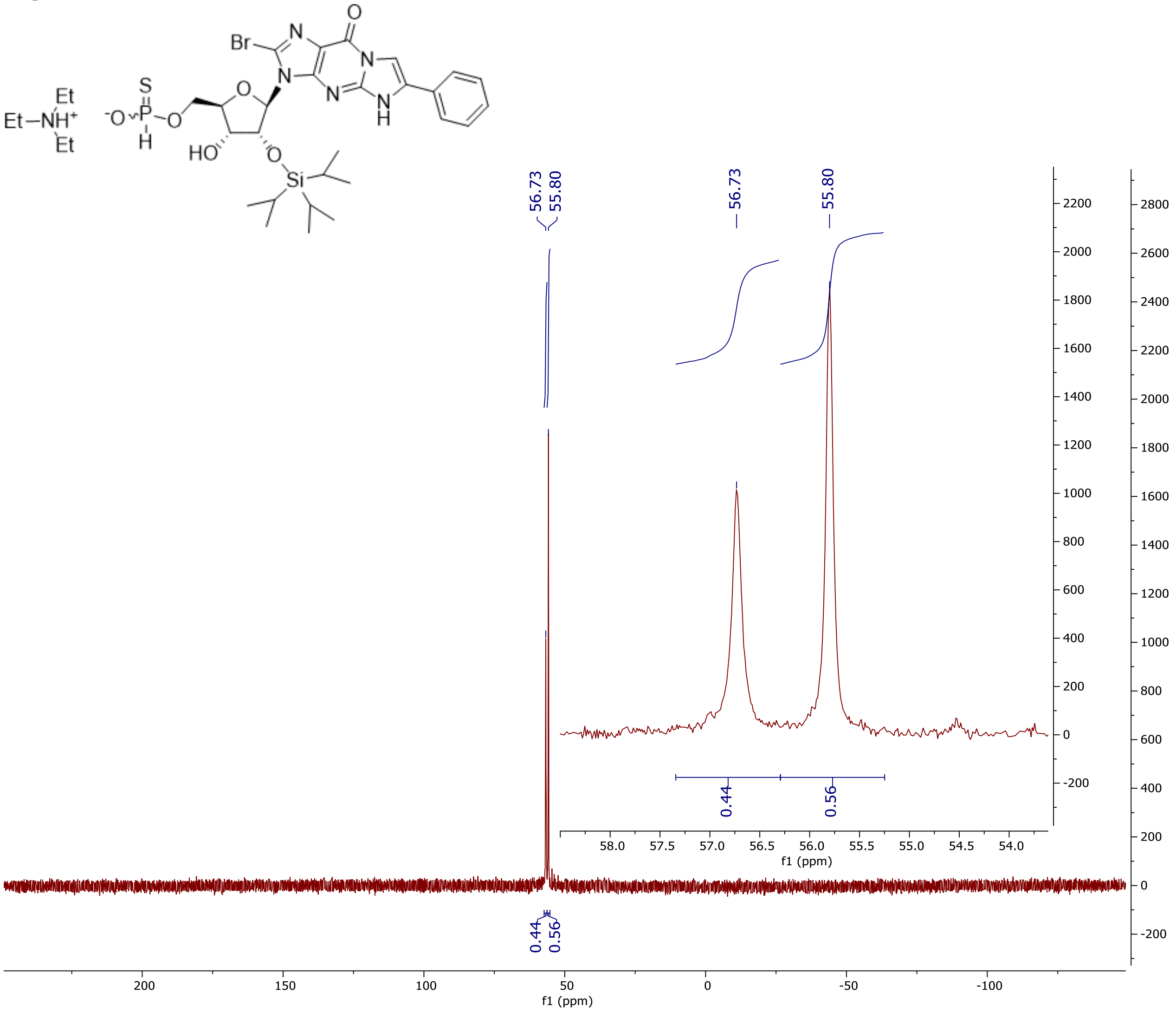

**Triethylammonium 8-Bromo-β-phenyl-1,$N^2$-etheno-2′-triisopropylsilyloxyguanosine-3′,5′-cyclicmonophosphorodithioate (3)**

**Figure S8: HPLC-UV**

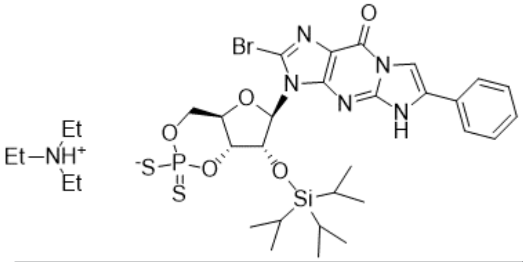

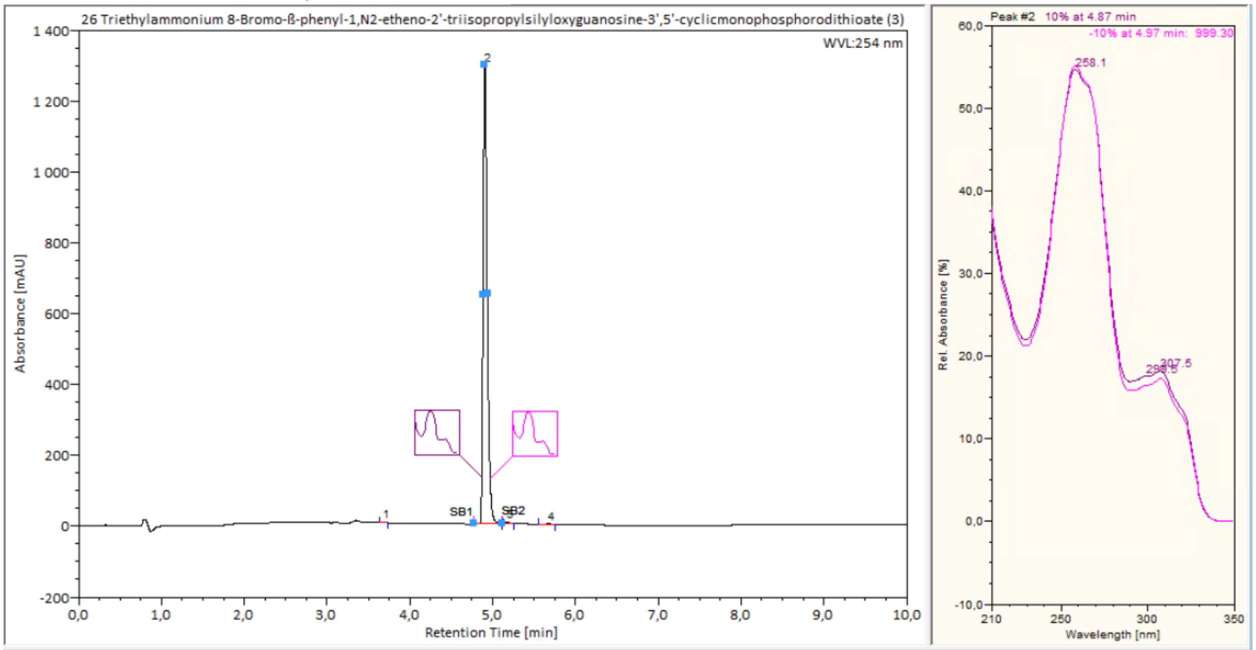


| A | B | C | D | E | F | G | H | I | J | K | L | M | N |
|---|---|---|---|---|---|---|---|---|---|---|---|---|---|
| No. | Peakname | Ret.Time min | RRT % | Area mAU*min | Rel.Area(ISTD) % | Type | Height mAU | Rel.Area % | Resolution | Calc Rel Amount unit | | | |
| 1 | n.a. | 3,675 | n.a. | 0,1230 | n.a. | BMB* | 2,385 | 0,16 | 14,07 | n.a. | | | |
| 2 | n.a. | 4,906 | n.a. | 74,9881 | n.a. | BMB | 1294,422 | 99,22 | 3,10 | n.a. | | | |
| 3 | n.a. | 5,174 | n.a. | 0,3054 | n.a. | BMB* | 5,898 | 0,40 | 5,65 | n.a. | | | |
| 4 | n.a. | 5,669 | n.a. | 0,1648 | n.a. | BMB* | 2,644 | 0,22 | n.a. | n.a. | | | |
| Total: | | | | 75,5814 | 0,0000 | | 1305,348 | 100,00 | | | | | |

**Figure S9: $^1$H-NMR ($CDCl_3$, 500 MHz)**

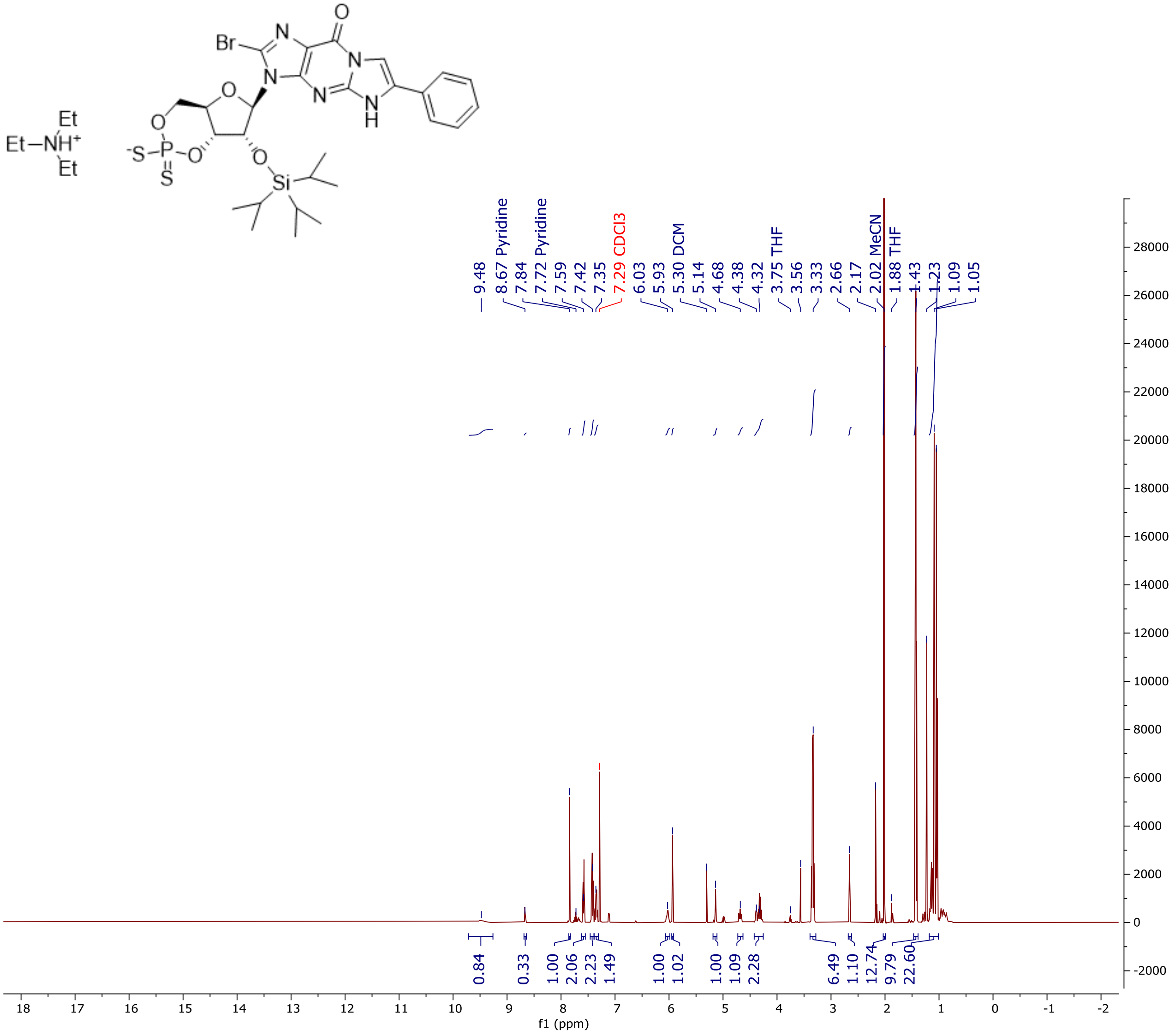

**Figure S10: $^{13}$C-NMR ($CD_3Cl$, 126 MHz)**

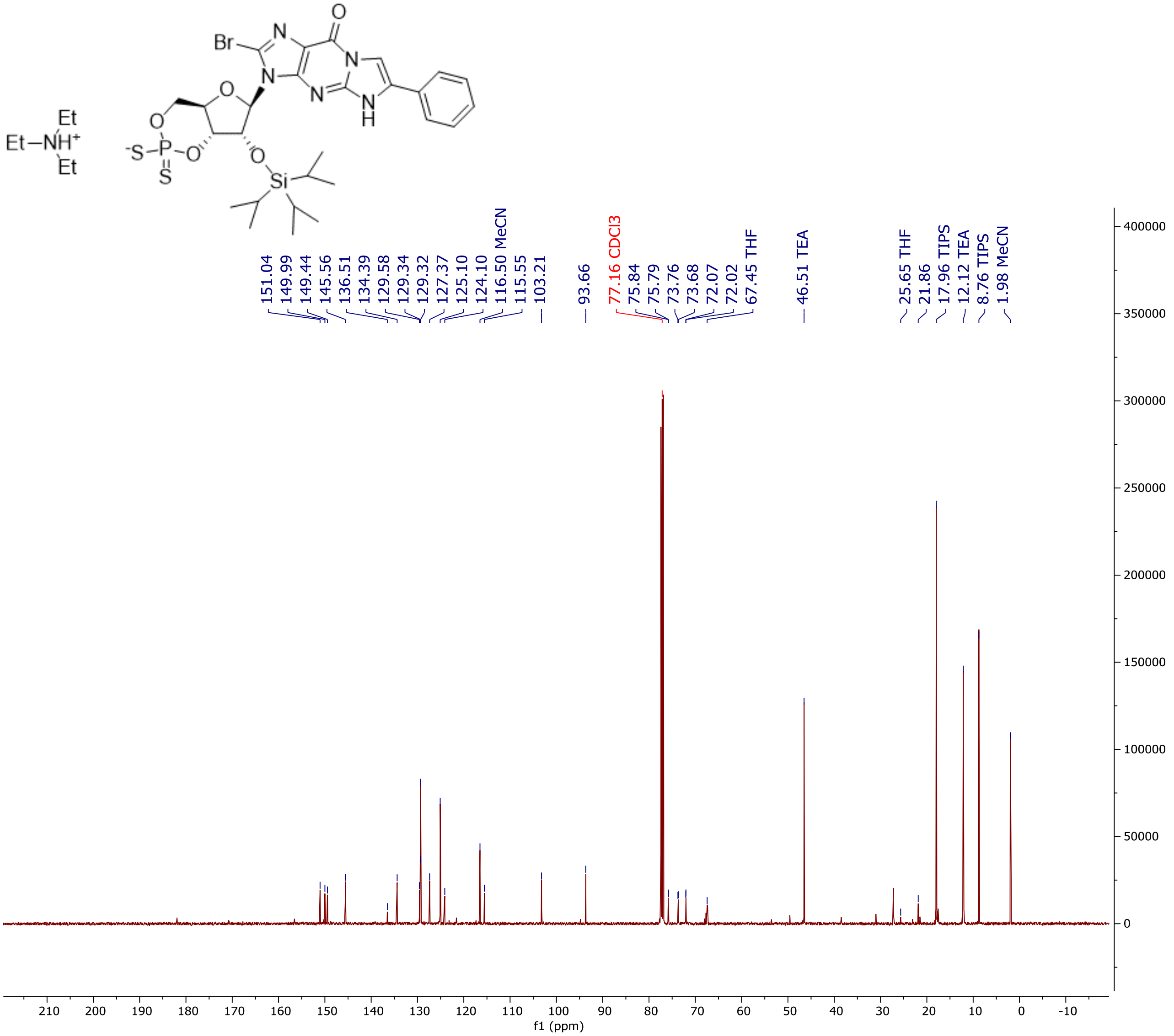

**Figure S11: $^{31}$P-NMR ($CDCl_3$, 203 MHz)**

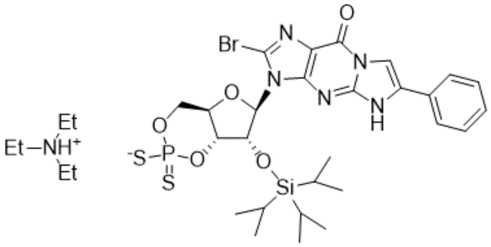

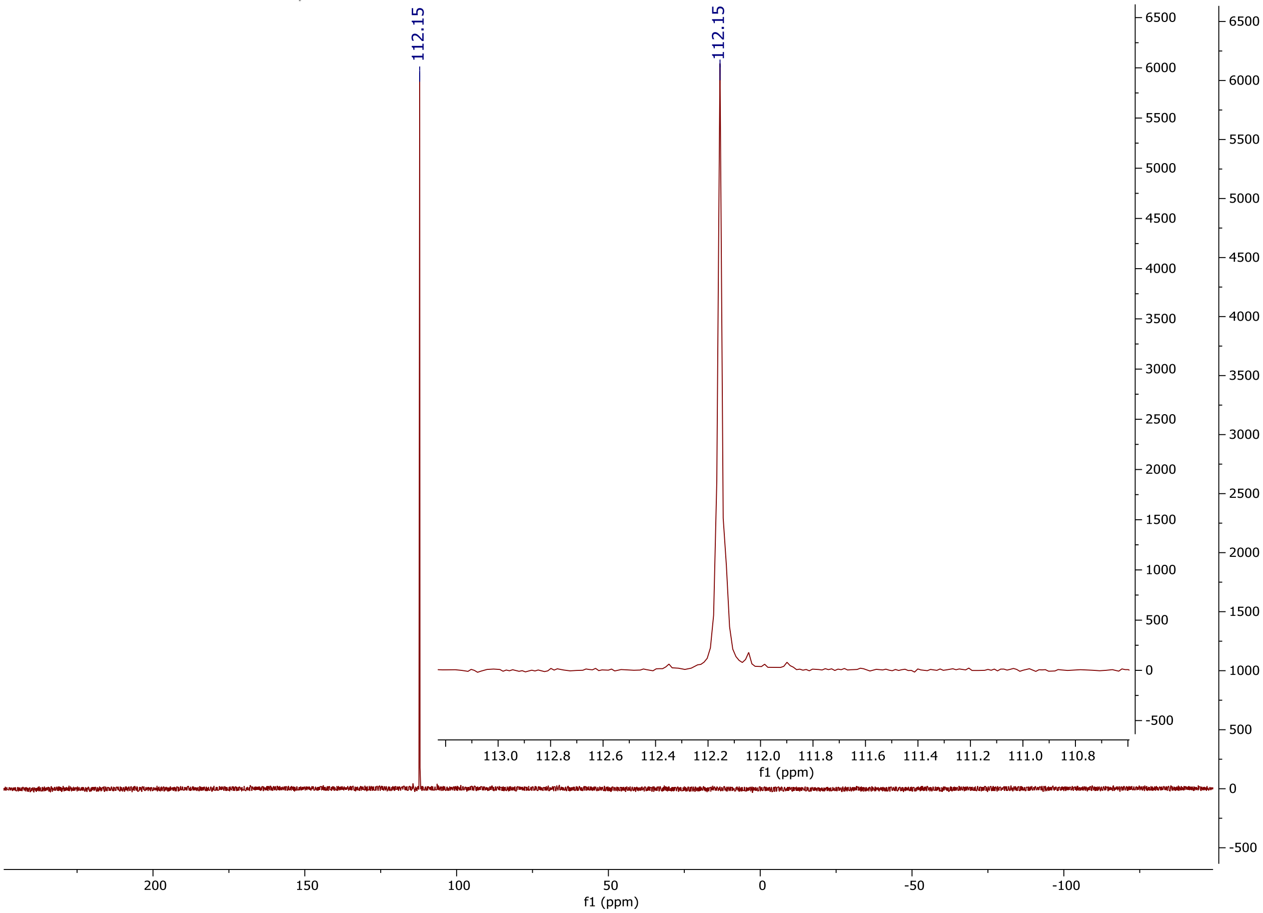

**Figure S12: $^{31}P\{^{1}H\}$-NMR ($CDCl_3$, 203 MHz)**

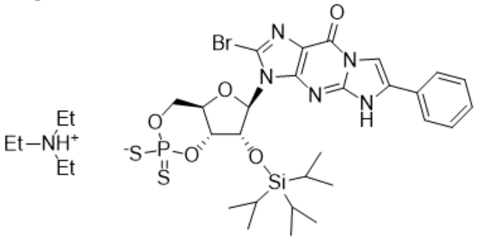

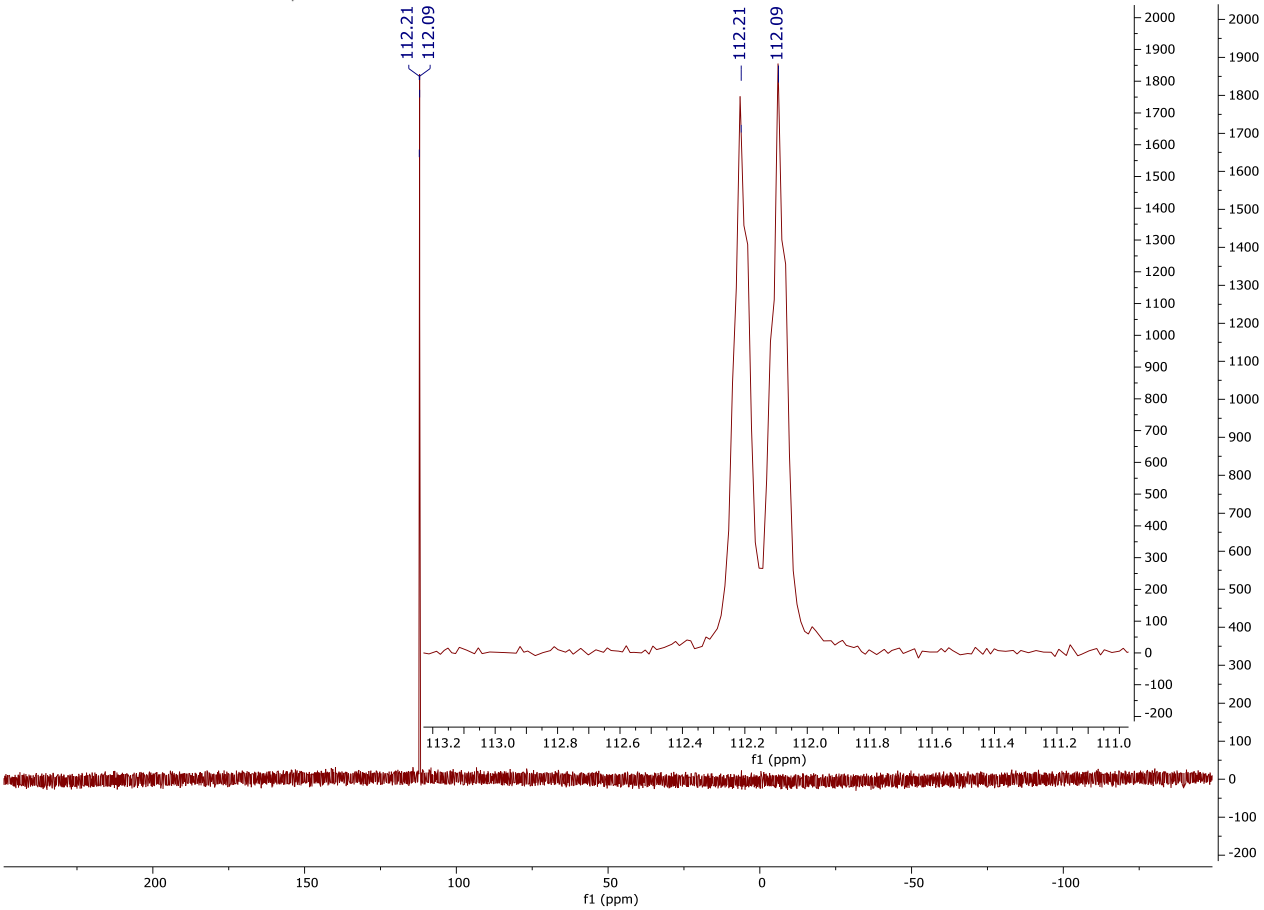

## Triethylammonium $S_P$-8-Bromo-β-phenyl-1,$N^2$-etheno-2′-triisopropylsilyloxyguanosine-3′,5′-cyclicmonophosphorothioate (6)

**Figure S13: HPLC-UV**

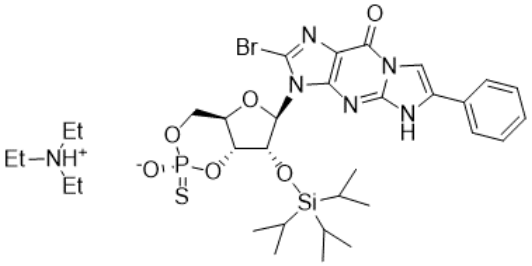

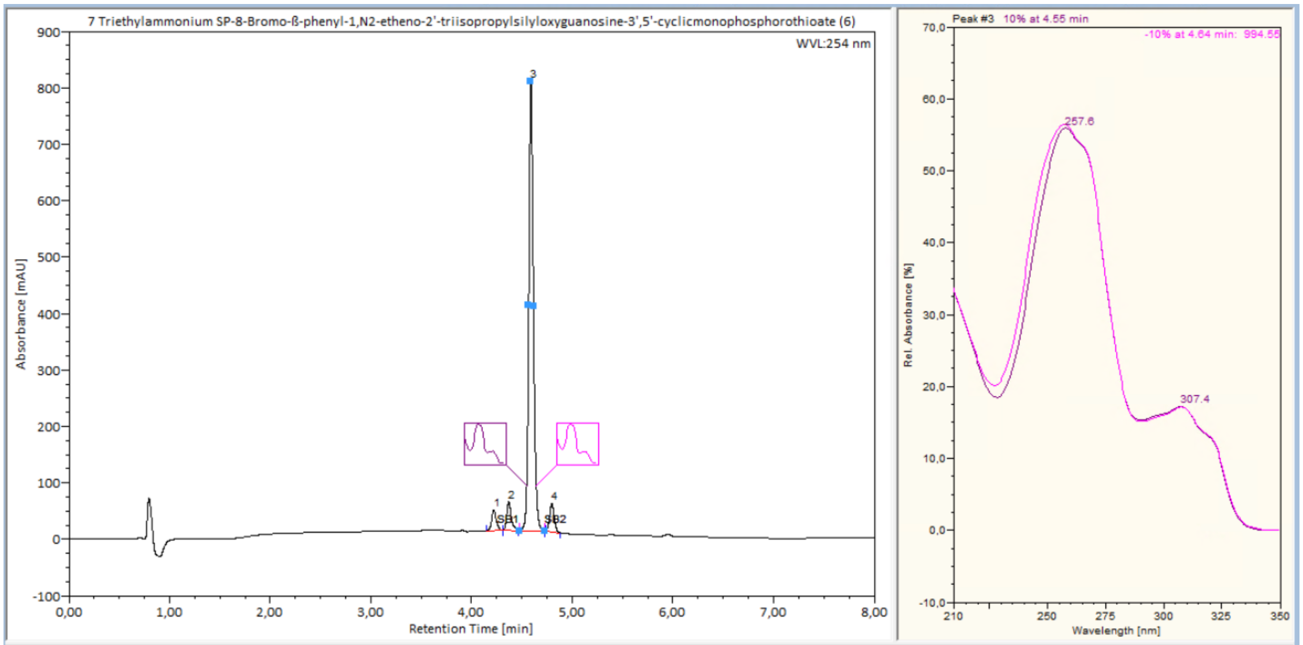


| No. | Peakname | Ret.Time min | RRT % | Area mAU*min | Rel.Area(ISTD) % | Type | Height mAU | Rel.Area % | Resolution | Calc Rel Amount unit |
|---|---|---|---|---|---|---|---|---|---|---|
| 1 | n.a. | 4,220 | n.a. | 1,9683 | n.a. | BMb* | 36,876 | 3,94 | 1,88 | n.a. |
| 2 | n.a. | 4,369 | n.a. | 2,6997 | n.a. | bMB* | 50,876 | 5,41 | 2,81 | n.a. |
| 3 | n.a. | 4,590 | n.a. | 42,6174 | n.a. | BMB | 797,716 | 85,40 | 2,58 | n.a. |
| 4 | n.a. | 4,797 | n.a. | 2,6171 | n.a. | BMB* | 50,534 | 5,24 | n.a. | n.a. |
| Total: | | | | 49,9024 | 0,0000 | | 936,003 | 100,00 | | |

**Figure S14: $^{1}$H-NMR ($CDCl_3$, 500 MHz)**

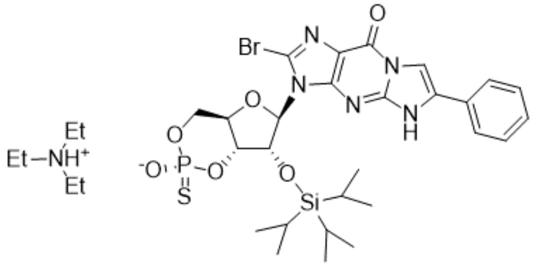

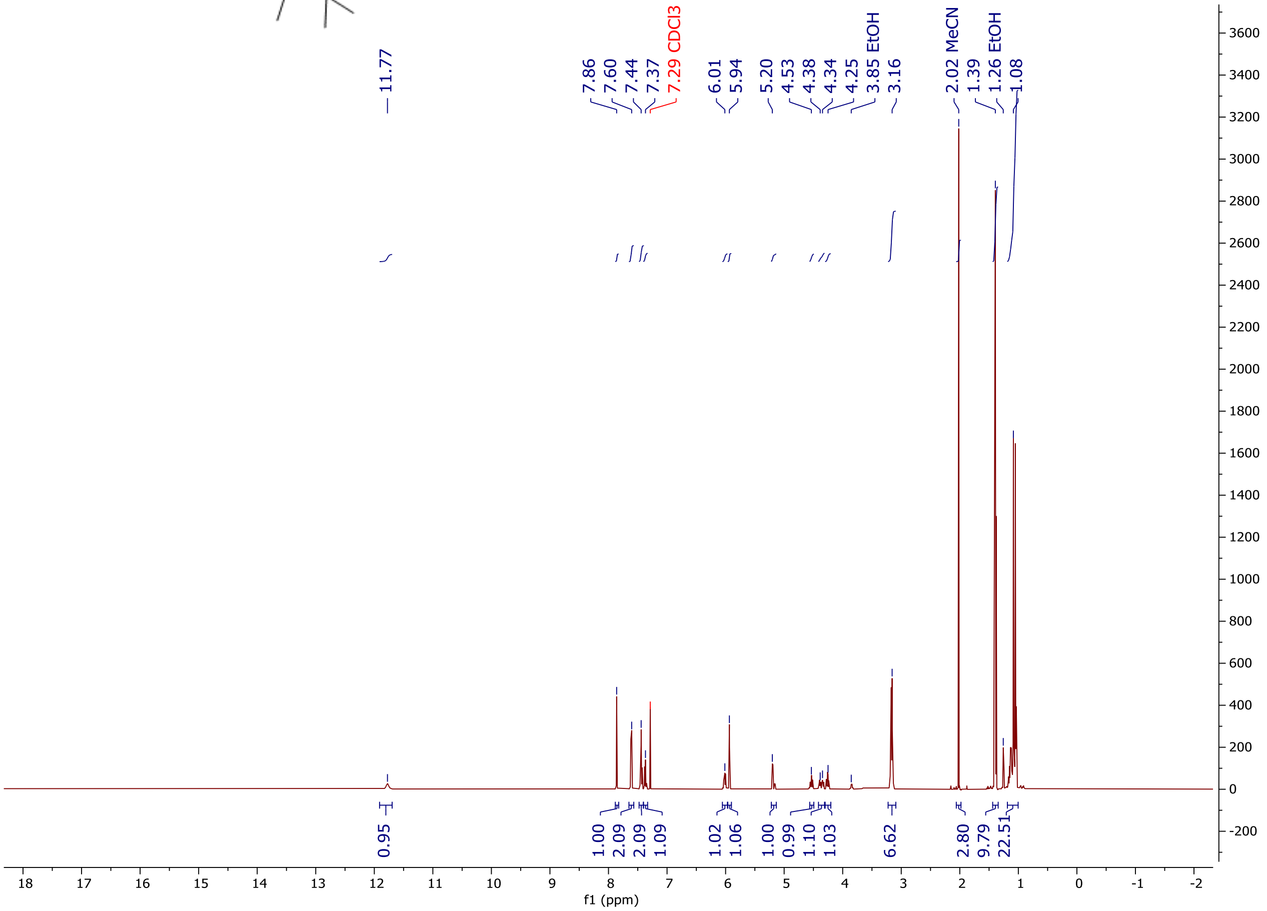

**Figure S15: $^{13}$C-NMR ($CD_3Cl$, 126 MHz)**

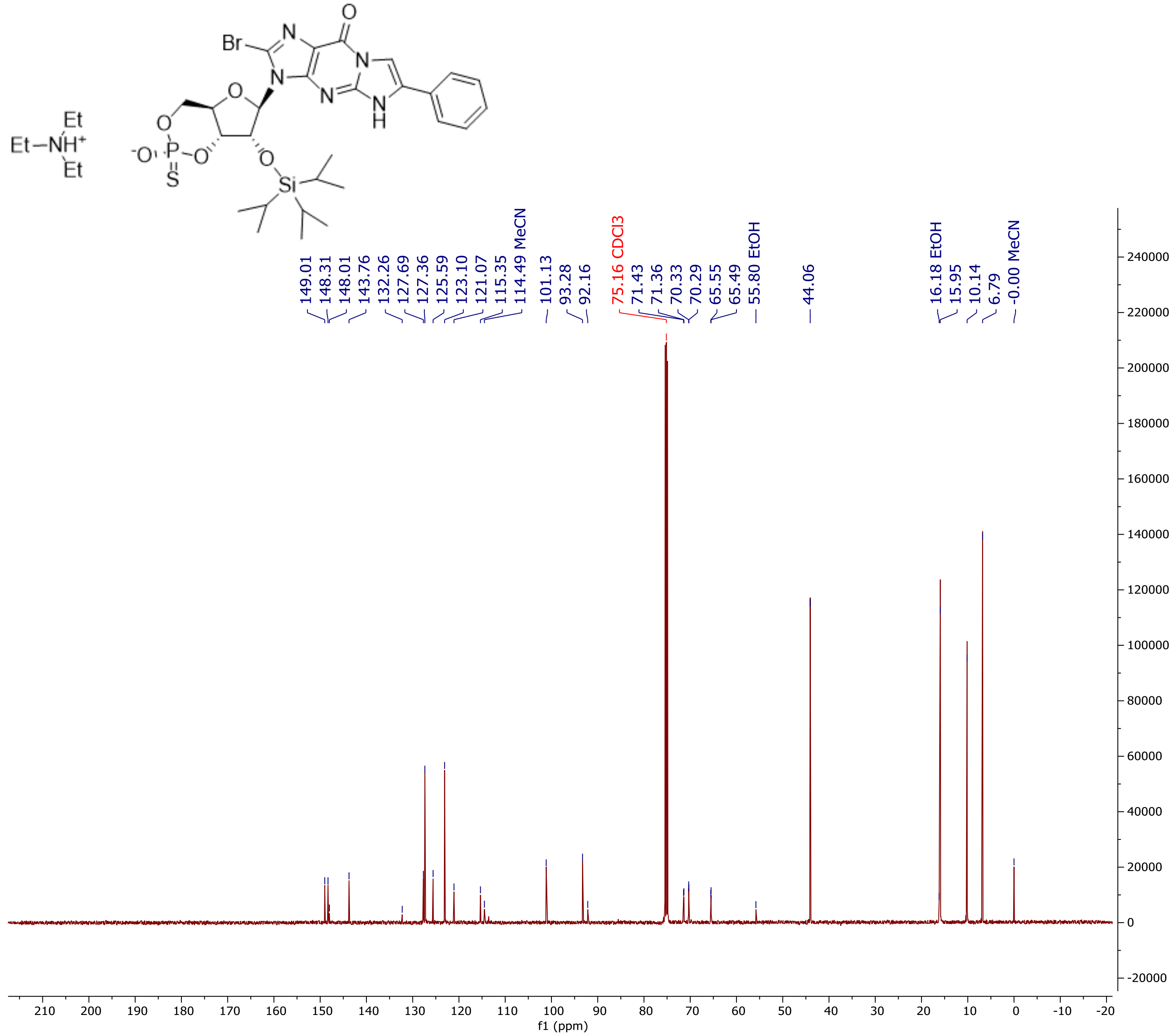

**Figure S16: $^{31}$P-NMR (CDCl$_3$, 203 MHz)**

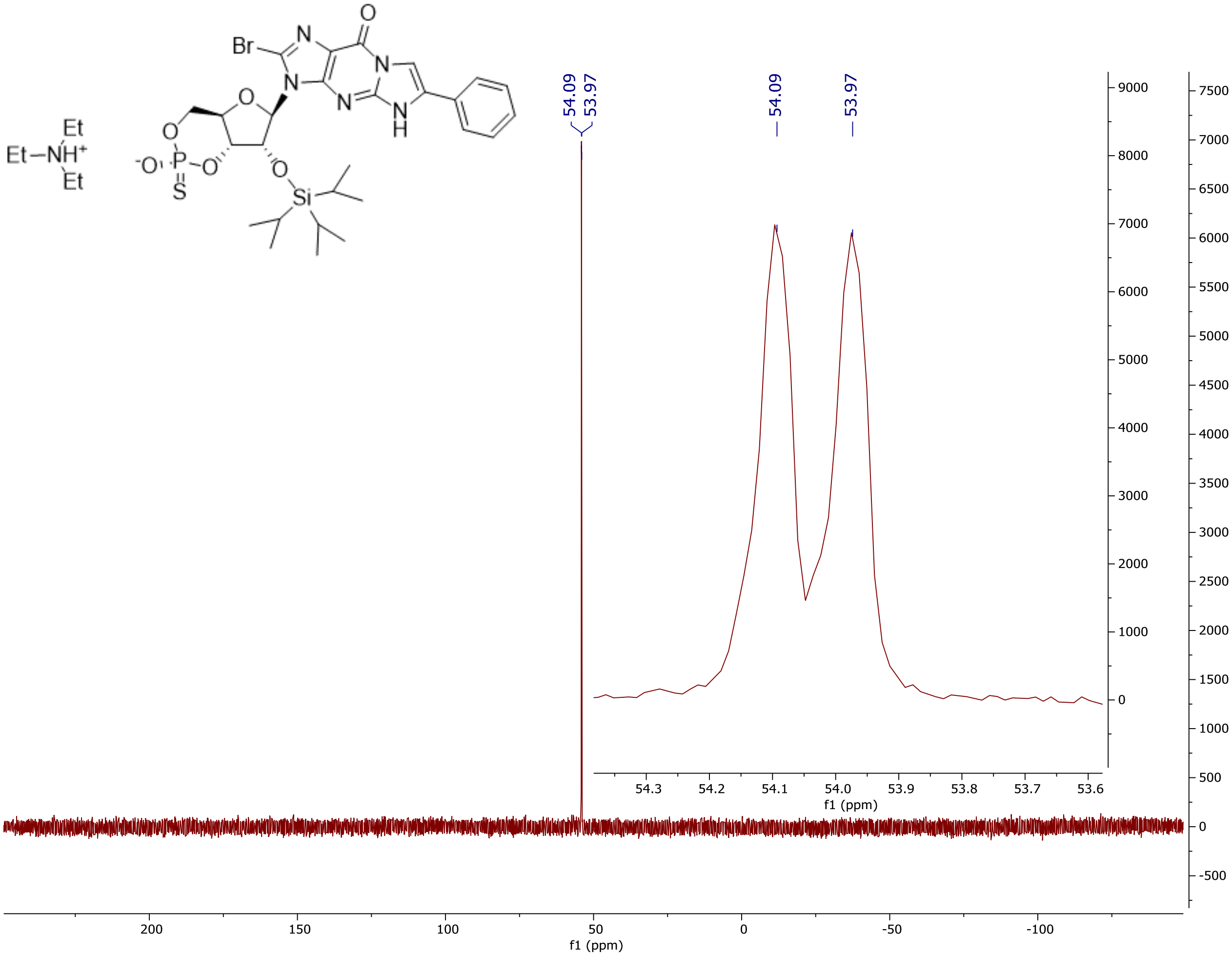

## Figure S17: $^{31}P\{^{1}H\}$-NMR ($CDCl_3$, 203 MHz)

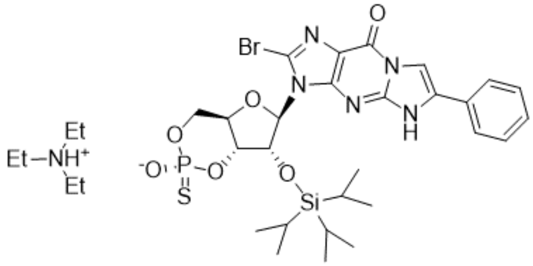


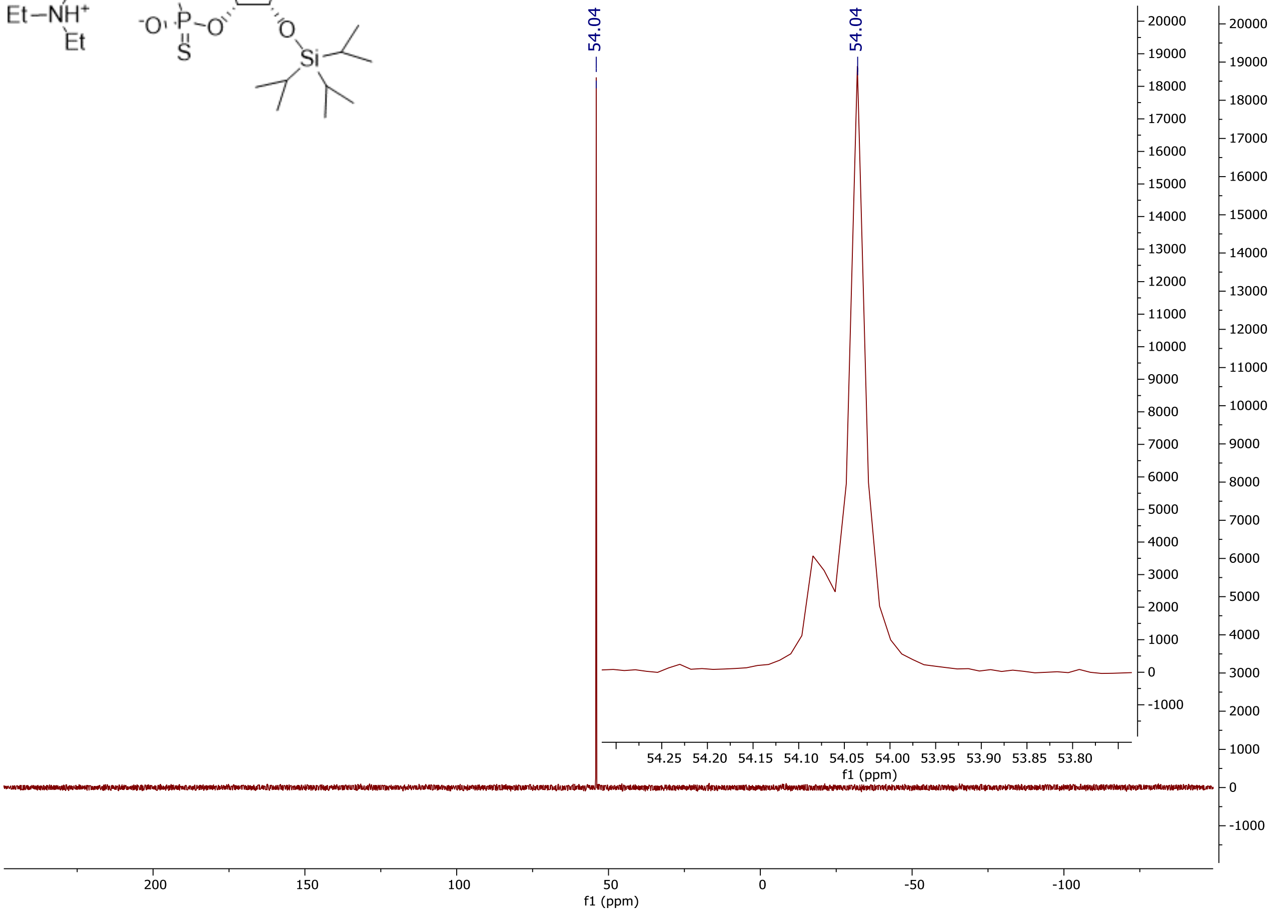

## Triethylammonium 8-Bromo-β-phenyl-1,$N^2$-etheno-2′-triisopropylsilyloxyguanosine-3′,5′-cyclicmonophosphate (7)

### Figure S18: HPLC-UV-MS

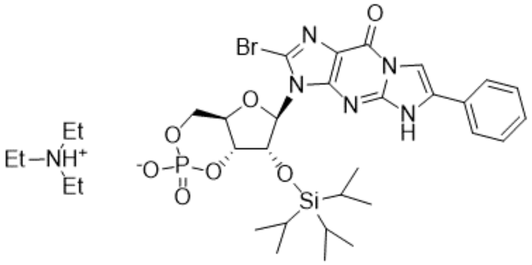


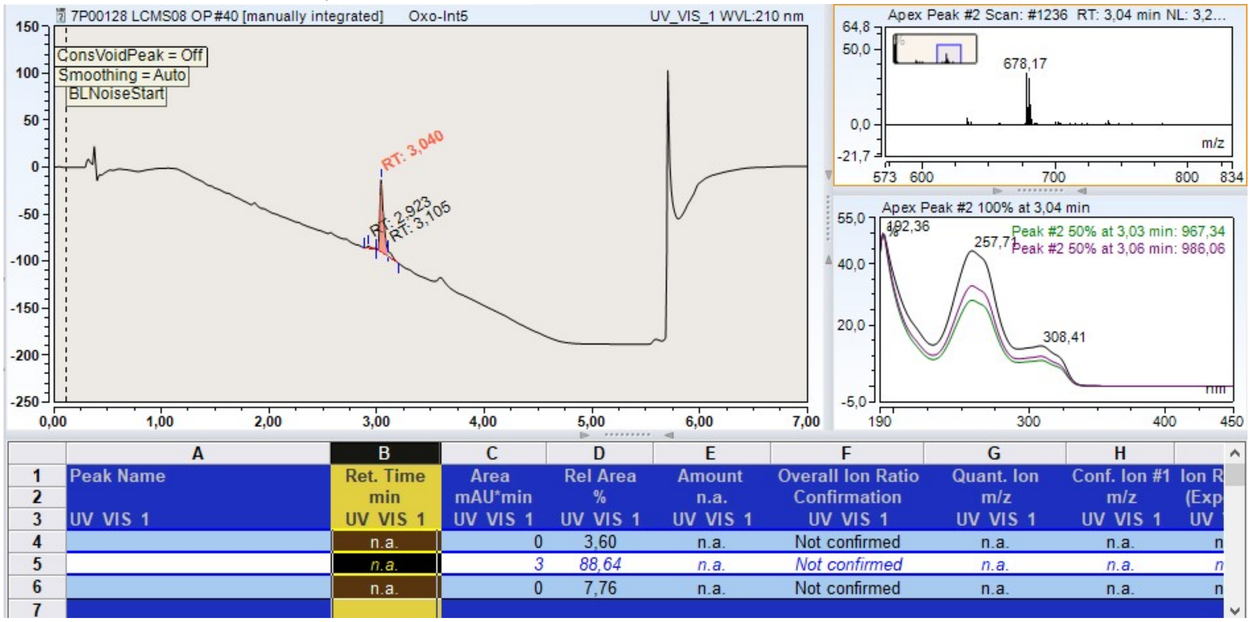


| | A | B | C | D | E | F | G | H | |
|---|---|---|---|---|---|---|---|---|---|
| 1 | Peak Name | Ret. Time | Area | Rel Area | Amount | Overall Ion Ratio | Quant. Ion | Conf. Ion #1 | Ion R |
| 2 | | min | mAU*min | % | n.a. | Confirmation | m/z | m/z | (Exp |
| 3 | UV VIS 1 | UV VIS 1 | UV VIS 1 | UV VIS 1 | UV VIS 1 | UV VIS 1 | UV VIS 1 | UV VIS 1 | UV |
| 4 | | n.a. | 0 | 3,60 | n.a. | Not confirmed | n.a. | n.a. | n |
| 5 | | n.a. | 3 | 88,64 | n.a. | Not confirmed | n.a. | n.a. | n |
| 6 | | n.a. | 0 | 7,76 | n.a. | Not confirmed | n.a. | n.a. | n |
| 7 | | | | | | | | | |

**Figure S19: $^{31}$P-NMR ($CDCl_3$, 203 MHz)**

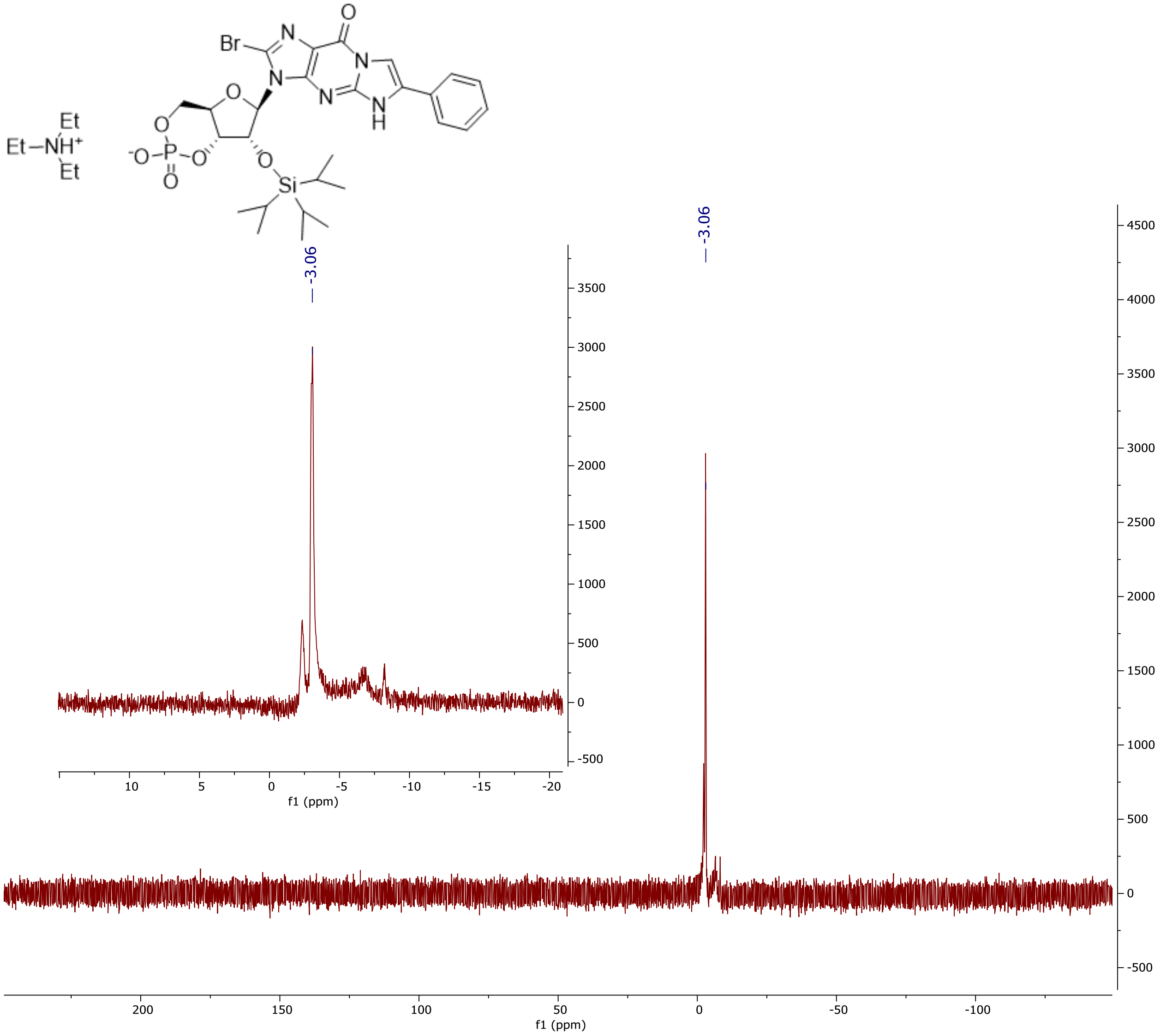

**Figure S20: $^{31}P\{^{1}H\}$-NMR ($CDCl_3$, 203 MHz)**

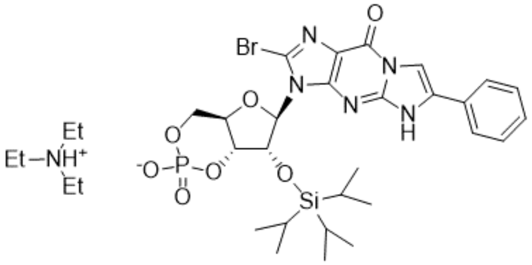

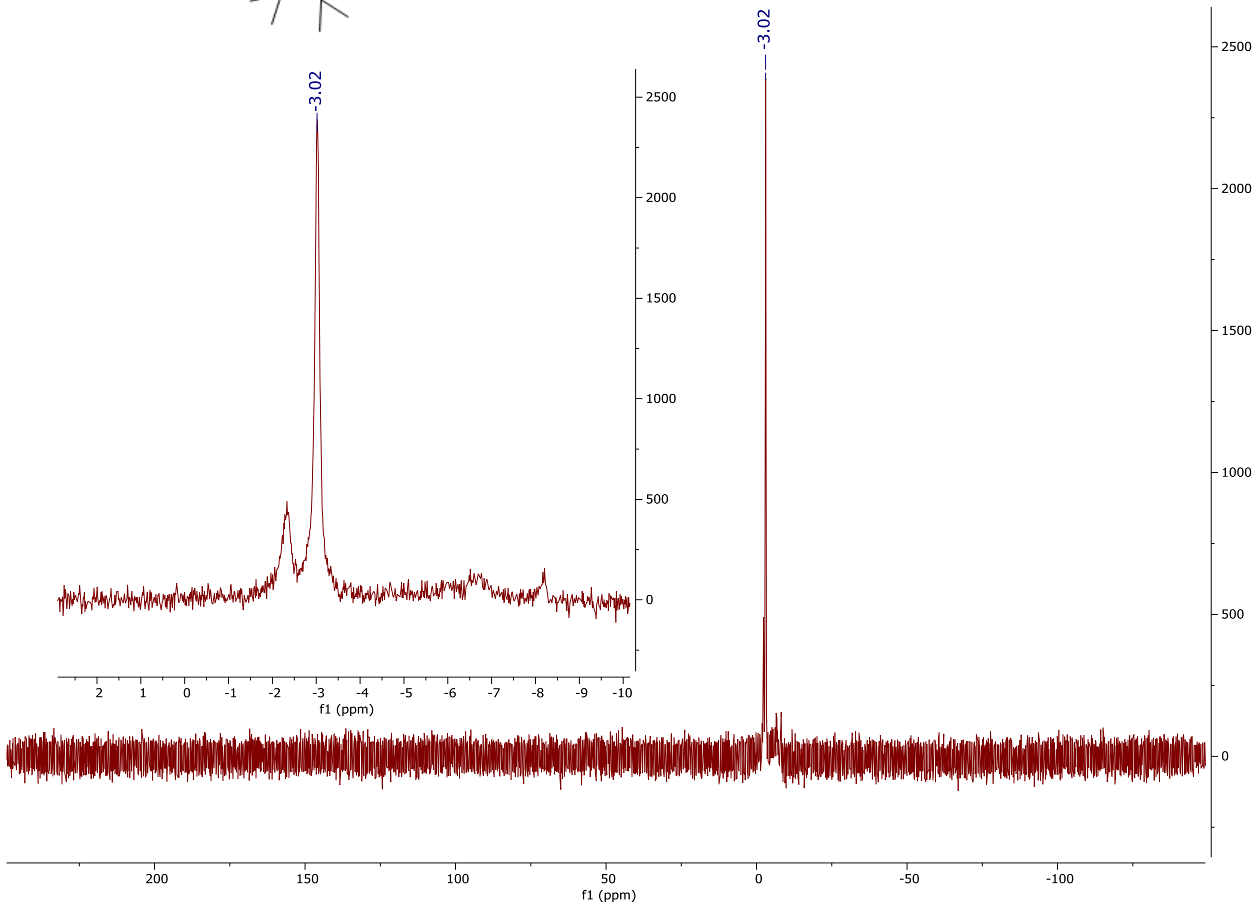

## Triethylammonium 8-Bromo-β-phenyl-1,$N^2$-ethenoguanosine-3′,5′-cyclicmonophosphorodithioate (Dithio-CN03)

**Figure S21: HPLC-UV**

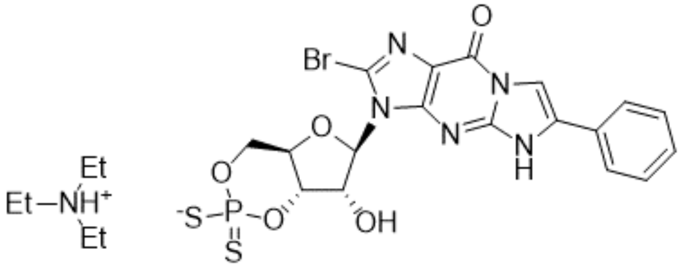


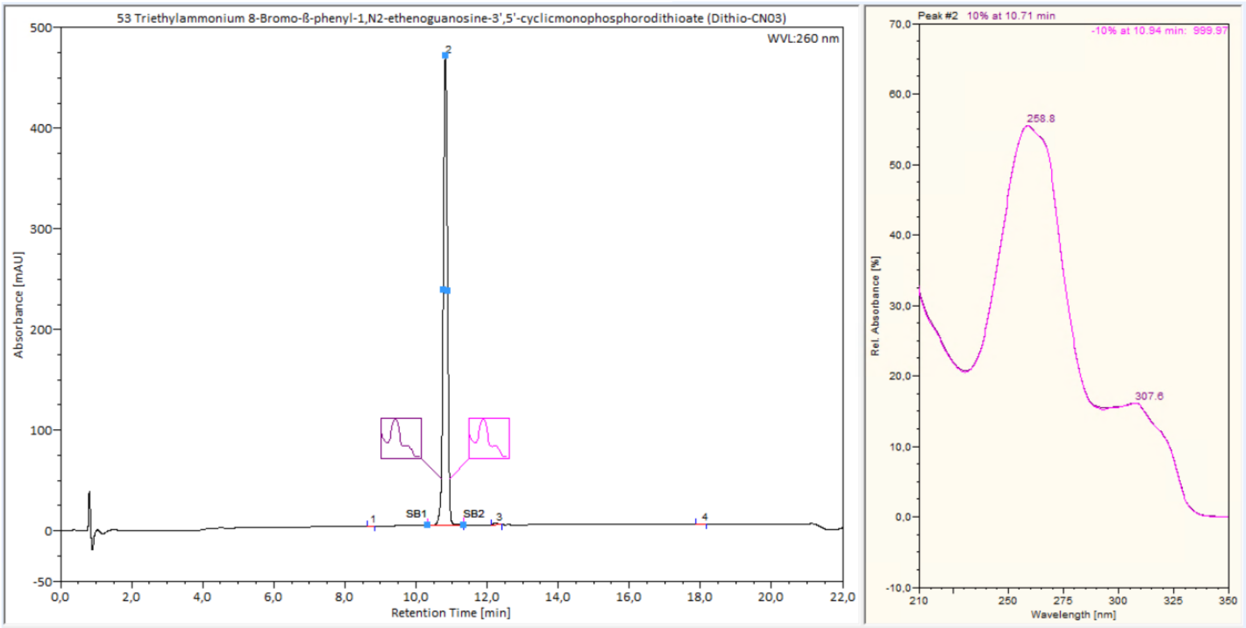


| A | B | C | D | E | F | G | H | I | J | K |
|---|---|---|---|---|---|---|---|---|---|---|
| No. | Peakname | Ret.Time min | RRT % | Area mAU*min | Rel.Area(ISTD) % | Type | Height mAU | Rel.Area % | Resolution | Calc Rel Amount unit |
| 1 | n.a. | 8,733 | n.a. | 0,0250 | n.a. | BMB* | 0,216 | 0,04 | 10,02 | n.a. |
| 2 | n.a. | 10,827 | n.a. | 64,2108 | n.a. | BMB | 466,257 | 99,55 | 6,18 | n.a. |
| 3 | n.a. | 12,256 | n.a. | 0,2245 | n.a. | BMB* | 1,534 | 0,35 | 22,93 | n.a. |
| 4 | n.a. | 18,043 | n.a. | 0,0423 | n.a. | BMB* | 0,275 | 0,07 | n.a. | n.a. |
| Total: | | | | 64,5026 | 0,0000 | | 468,282 | 100,00 | | |

**Figure S22: XRPD**

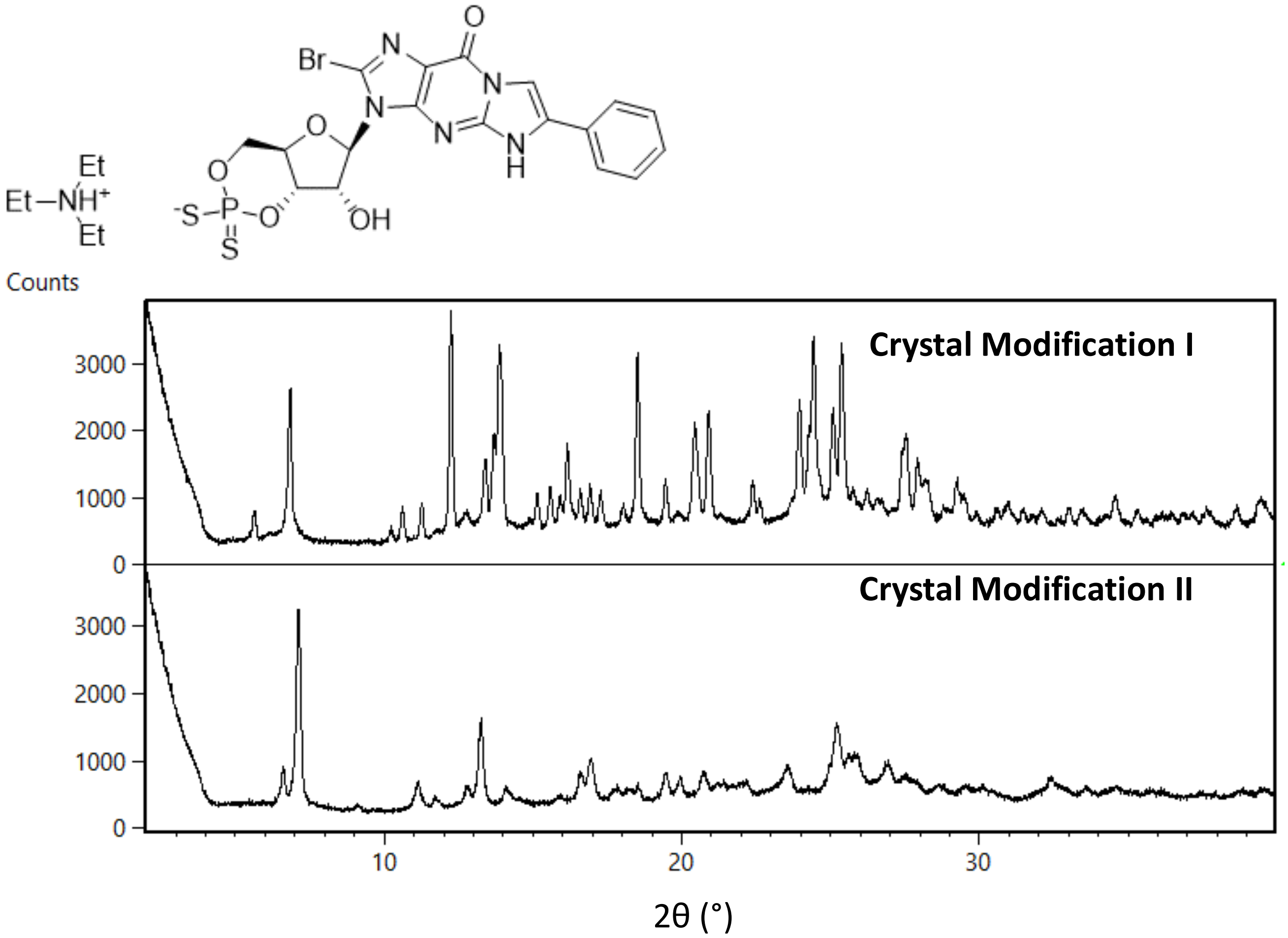

**Figure S23: $^1$H-NMR ($(CD_3)_2SO$, 500 MHz)**

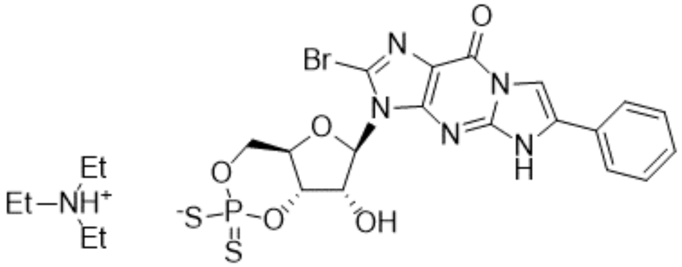

**Crystal Modification I**

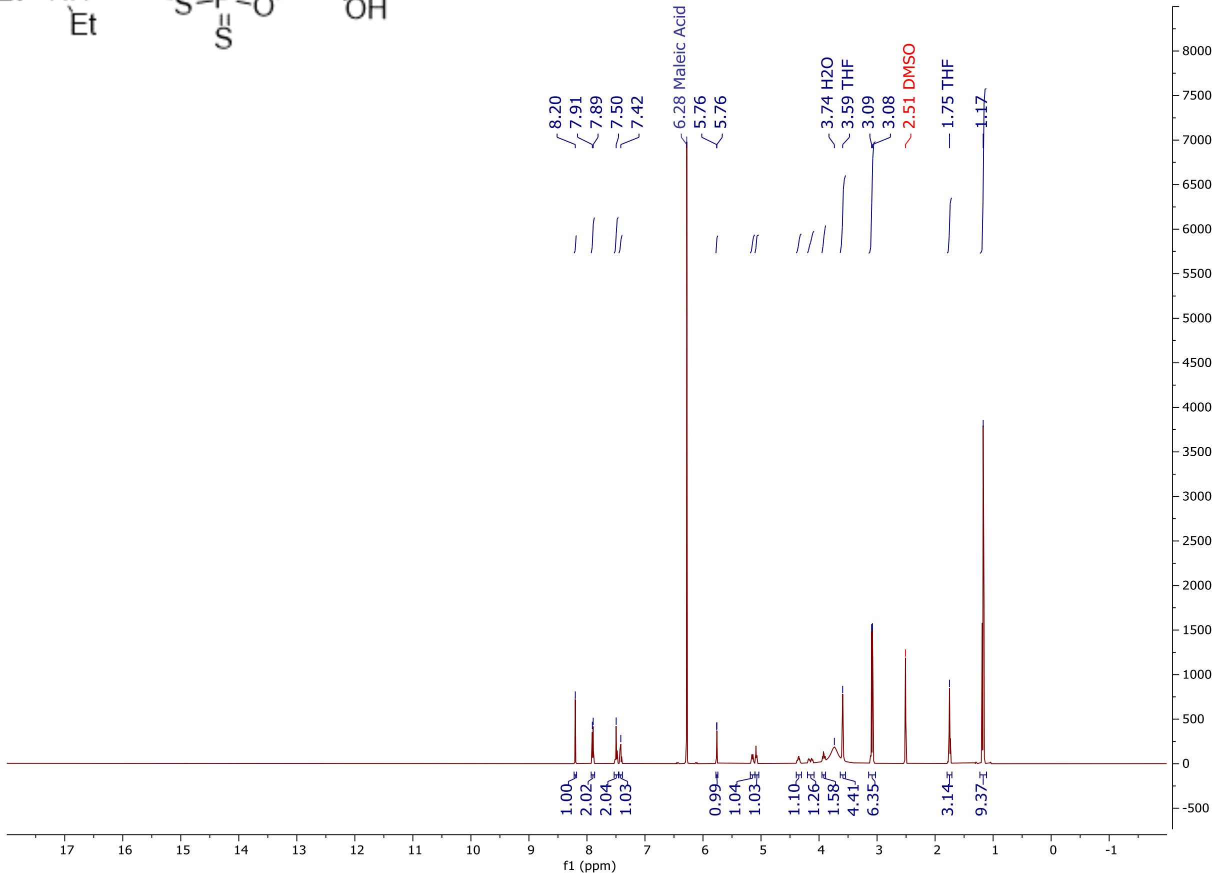

**Figure S24: $^{1}$H-NMR ($(CD_3)_2SO$, 500 MHz)**

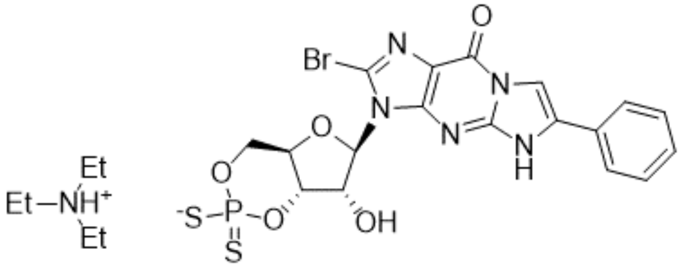


**Crystal Modification II**

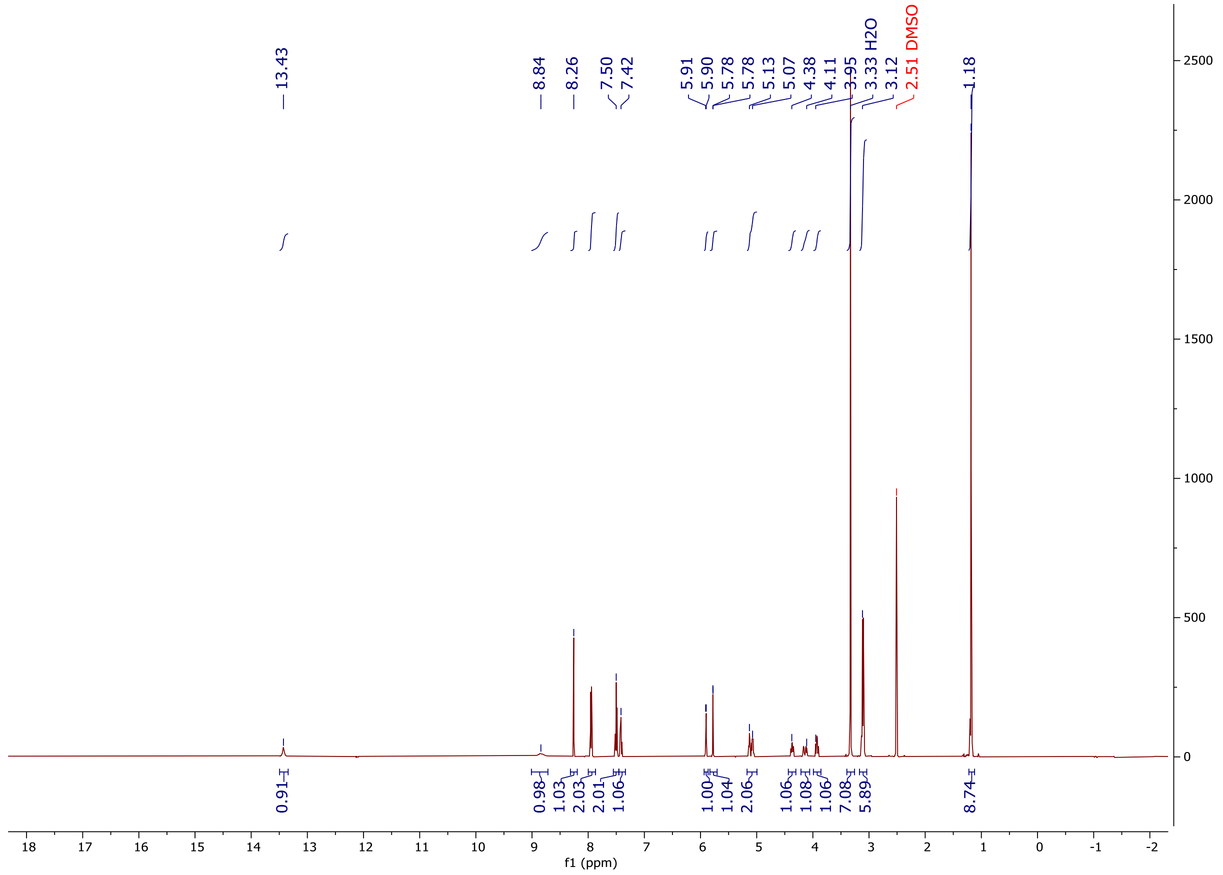

**Figure S25: $^{13}$C-NMR ($(CD_3)_2SO$, 126 MHz)**

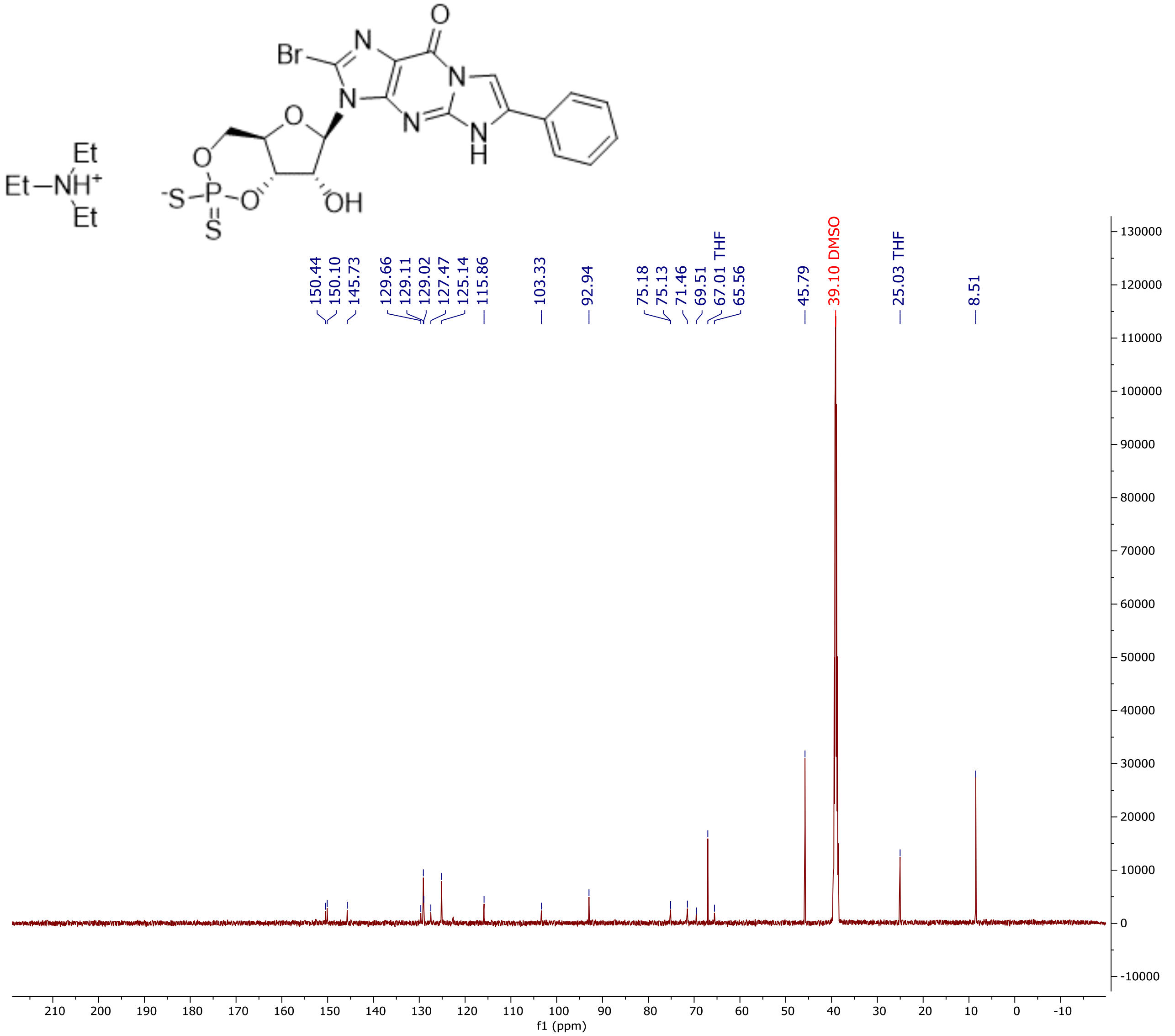

**Figure S26: $^{31}$P-NMR ($(CD_3)_2SO$, 203 MHz)**

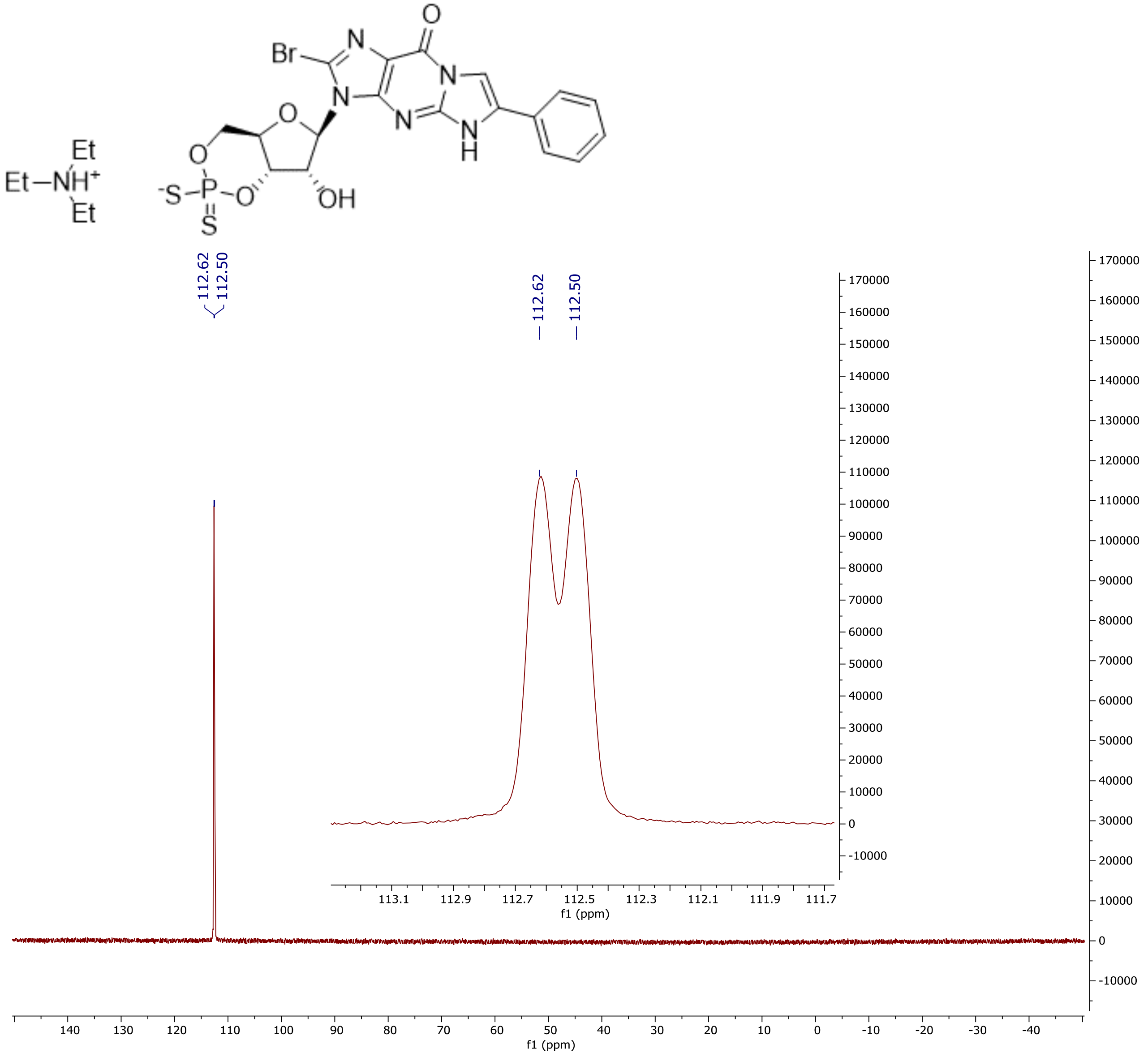

**Figure S27: $^{31}P\{^{1}H\}$-NMR ($(CD_3)_2SO$, 203 MHz)**

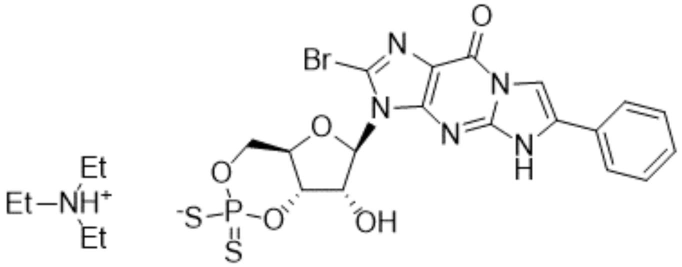


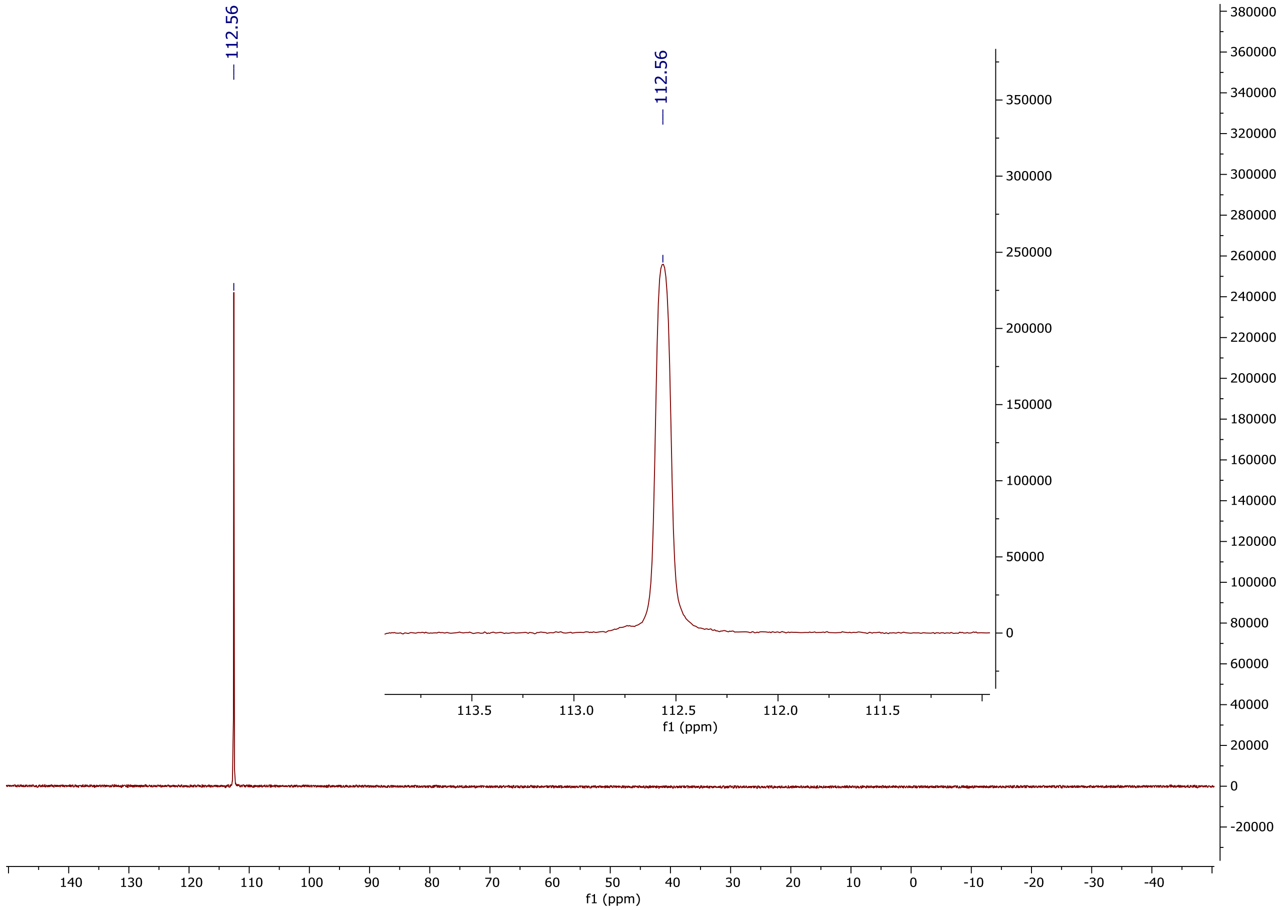

## Triethylammonium *S*P-8-Bromo-β-phenyl-1,*N*[2]-ethenoguanosine-3′,5′-cyclicmonophosphorothioate (*S*P-CN03)

**Figure S28: HPLC-UV**

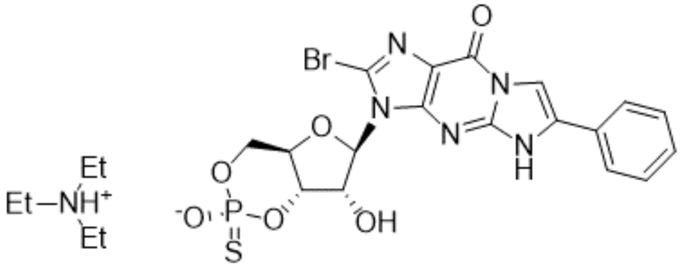

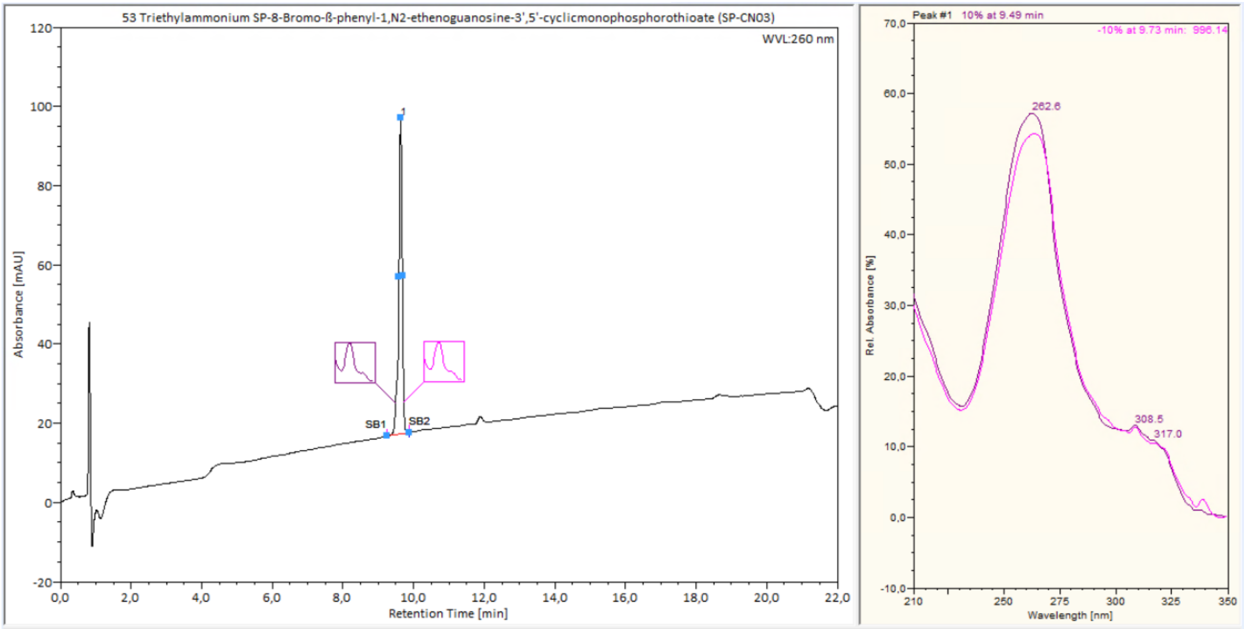


| A | B | C | D | E | F | G | H | I | J | K | L | M | N | O | P |
|---|---|---|---|---|---|---|---|---|---|---|---|---|---|---|---|
| No. | Peakname | Ret.Time min | RRT % | Area mAU*min | Rel.Area(ISTD) % | Type | Height mAU | Rel.Area % | Resolution | Calc Rel Amount unit | | | | | |
| 1 | n.a. | 9,633 | n.a. | 9,1898 | n.a. | BMB | 79,786 | 100,00 | n.a. | n.a. | | | | | |
| Total: | | | | 9,1898 | 0,0000 | | 79,786 | 100,00 | | | | | | | |

**Figure S29: $^{1}$H-NMR ($(CD_3)_2SO$, 500 MHz)**

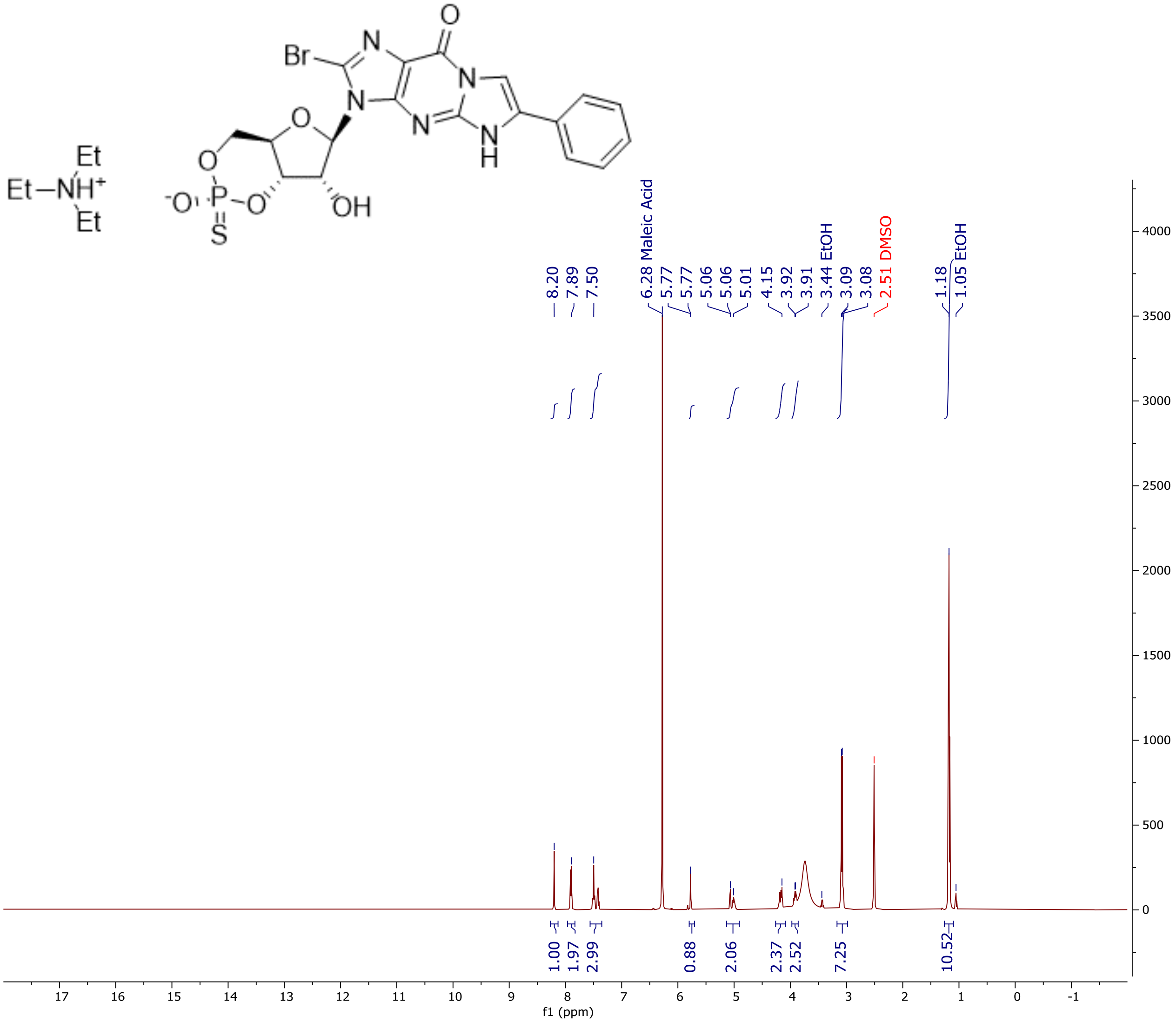

**Figure S30: $^{13}$C-NMR ($(CD_3)_2SO$, 126 MHz)**

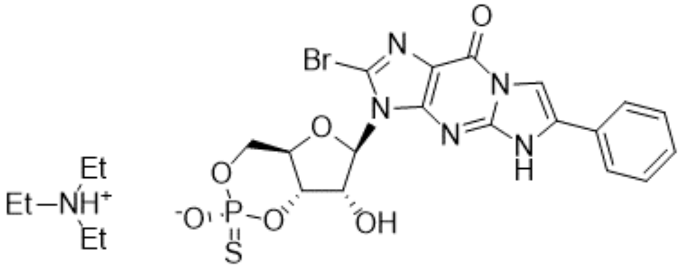

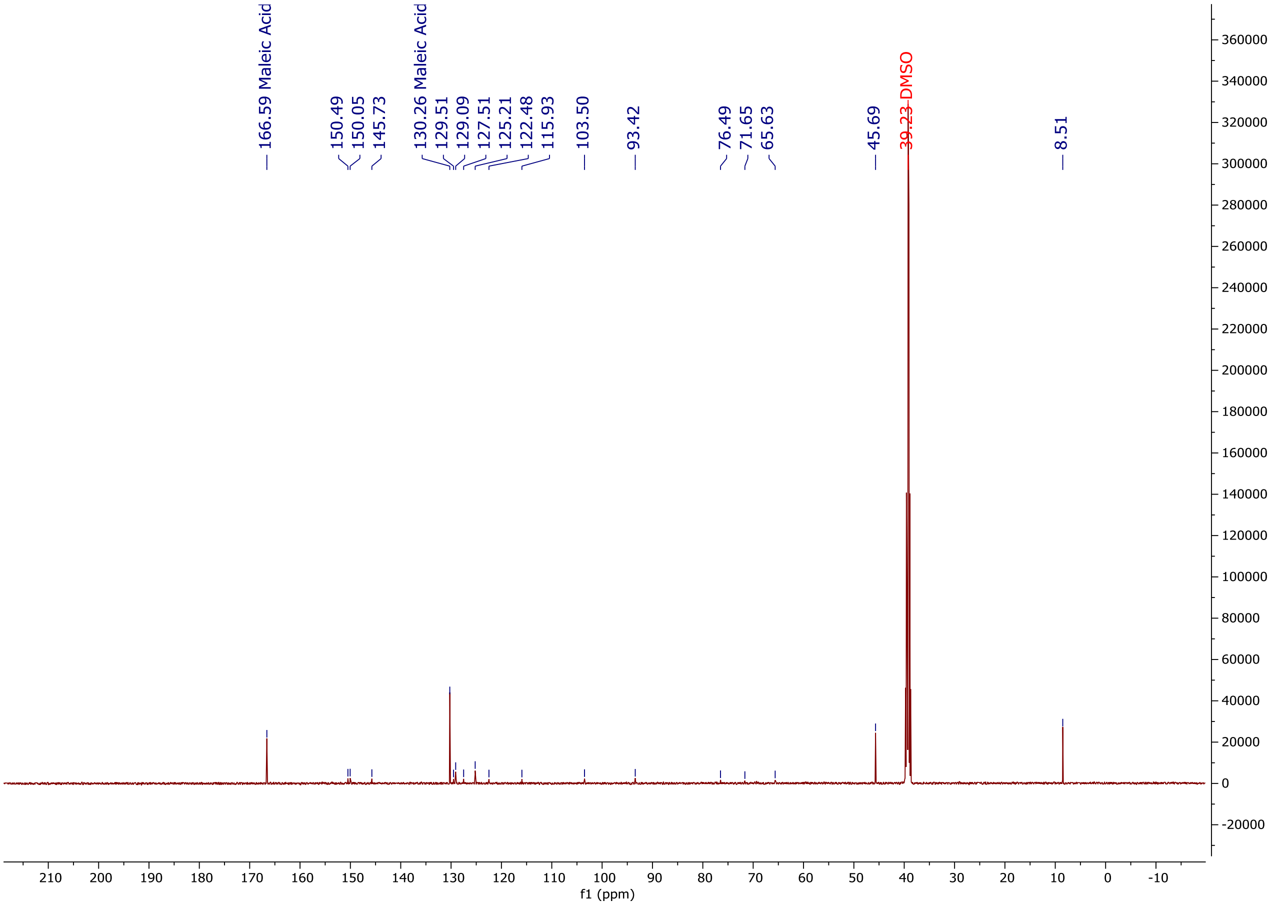

**Figure S31: $^{31}$P-NMR ($(CD_3)_2SO$, 203 MHz)**

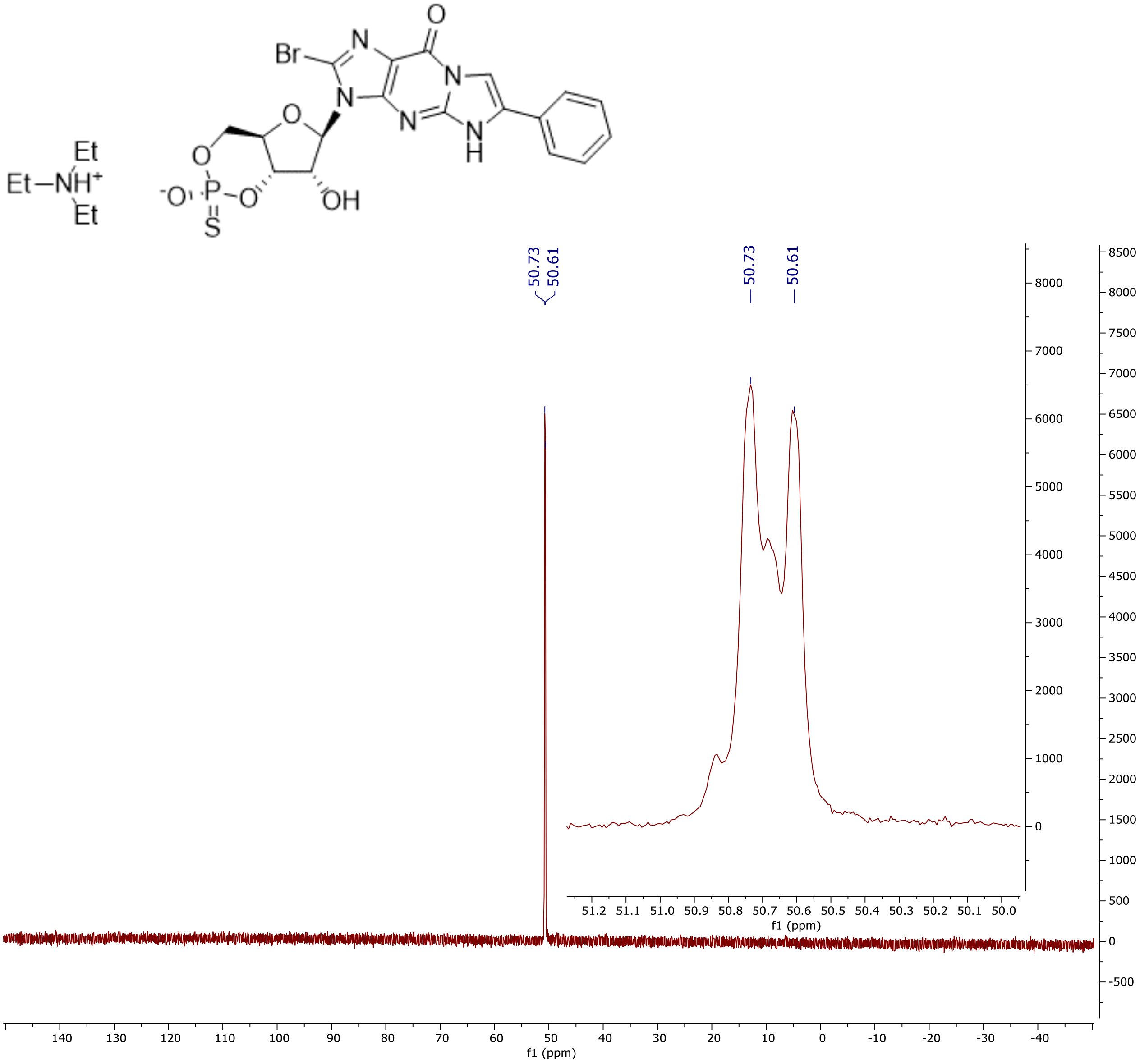

**Figure S32: $^{31}P\{^{1}H\}$-NMR ($(CD_3)_2SO$, 203 MHz)**

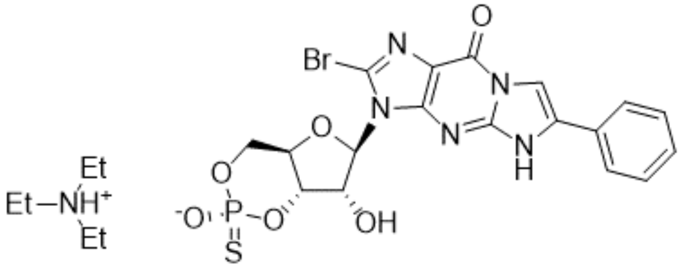


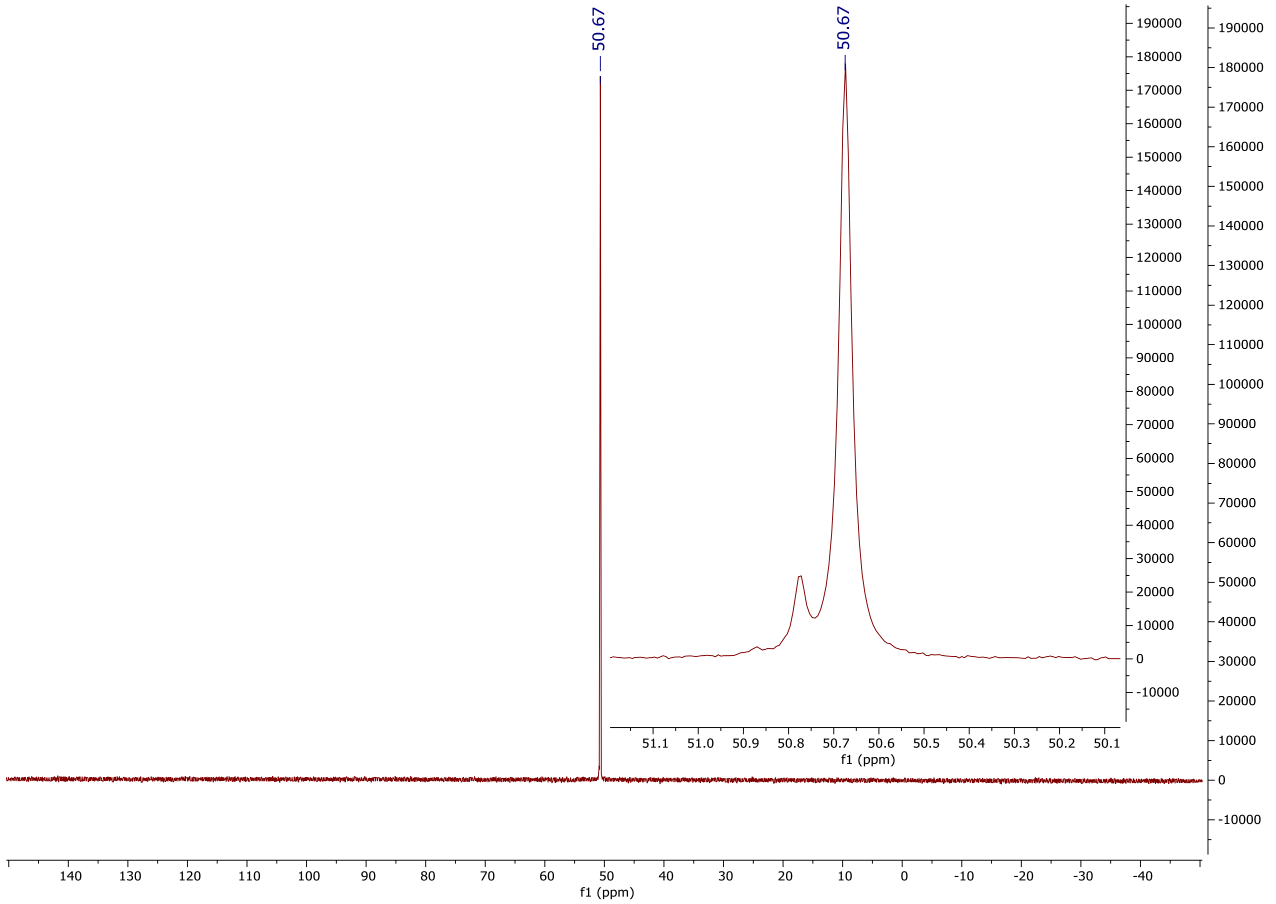

## Triethylammonium 8-Bromo-β-phenyl-1,$N^2$-ethenoguanosine-3′,5′-cyclicmonophosphate (Oxo-CN03)

**Figure S33: HPLC-UV**

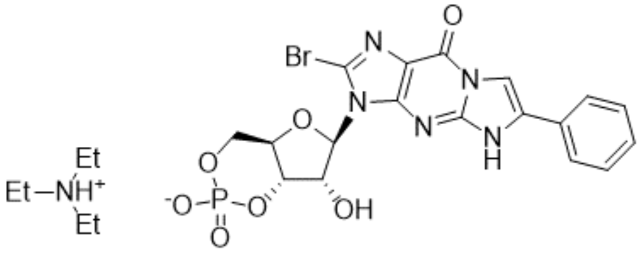

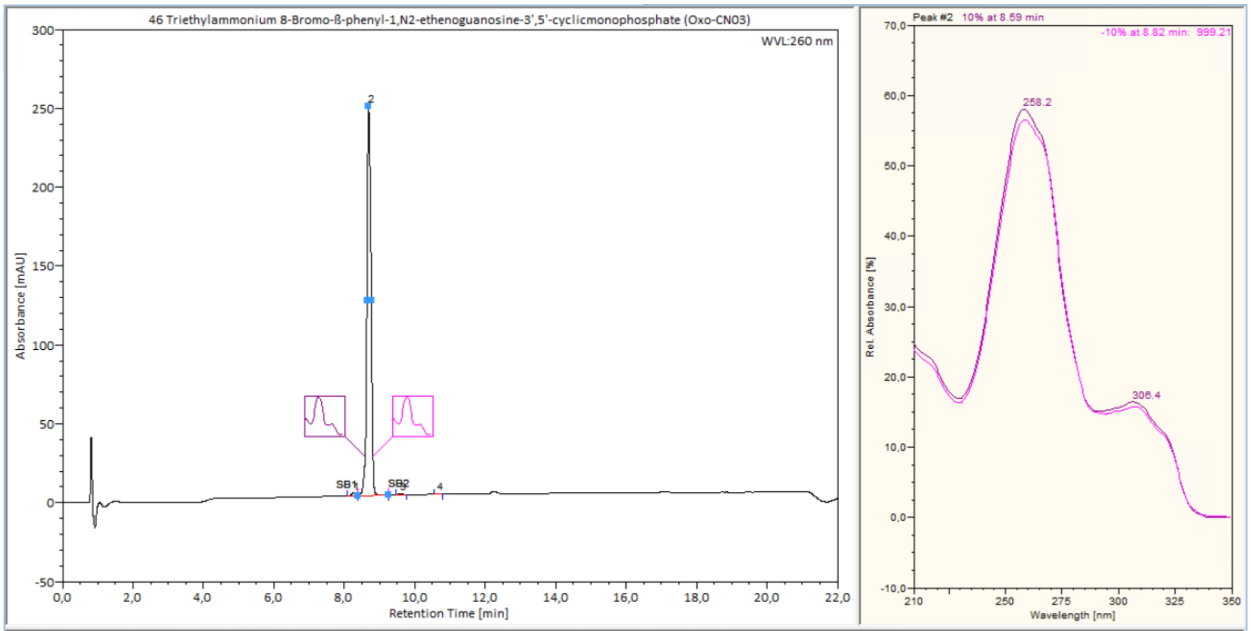


| A | B | C | D | E | F | G | H | I | J | K |
|---|---|---|---|---|---|---|---|---|---|---|
| No. | Peakname | Ret.Time min | RRT % | Area mAU*min | Rel.Area(ISTD) % | Type | Height mAU | Rel.Area % | Resolution | Calc Rel Amount unit |
| 1 | n.a. | 8,256 | n.a. | 0,2116 | n.a. | BMB* | 1,806 | 0,63 | 2,09 | n.a. |
| 2 | n.a. | 8,689 | n.a. | 33,4773 | n.a. | BMB | 246,749 | 99,02 | 4,21 | n.a. |
| 3 | n.a. | 9,578 | n.a. | 0,0877 | n.a. | BMB* | 0,721 | 0,26 | 4,77 | n.a. |
| 4 | n.a. | 10,641 | n.a. | 0,0318 | n.a. | BMB* | 0,234 | 0,09 | n.a. | n.a. |
| Total: | | | | 33,8084 | 0,0000 | | 249,510 | 100,00 | | |

**Figure S34: $^1$H-NMR ($(CD_3)_2SO$, 500 MHz)**

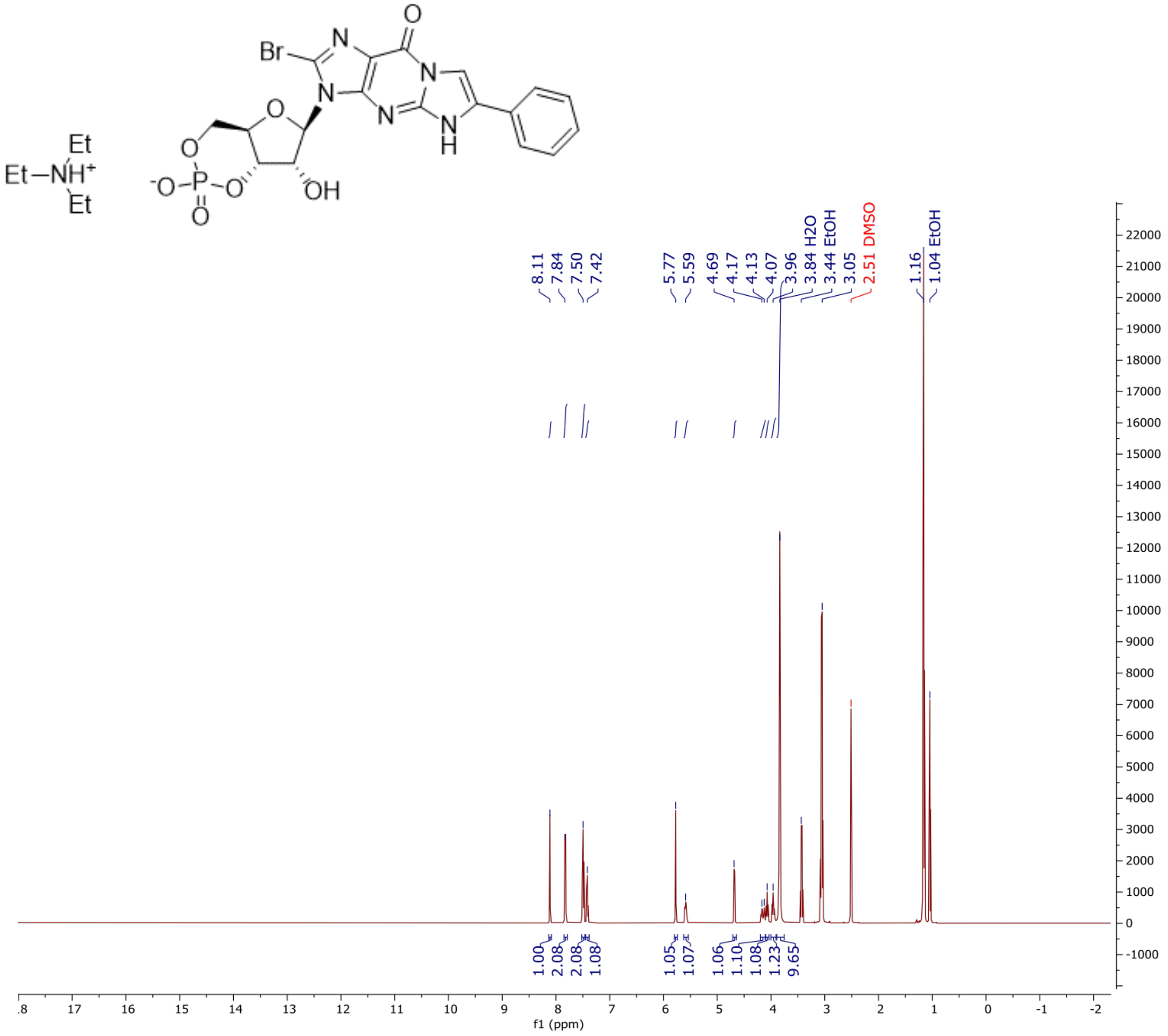

**Figure S35: $^{13}$C-NMR ($(CD_3)_2SO$, 126 MHz)**

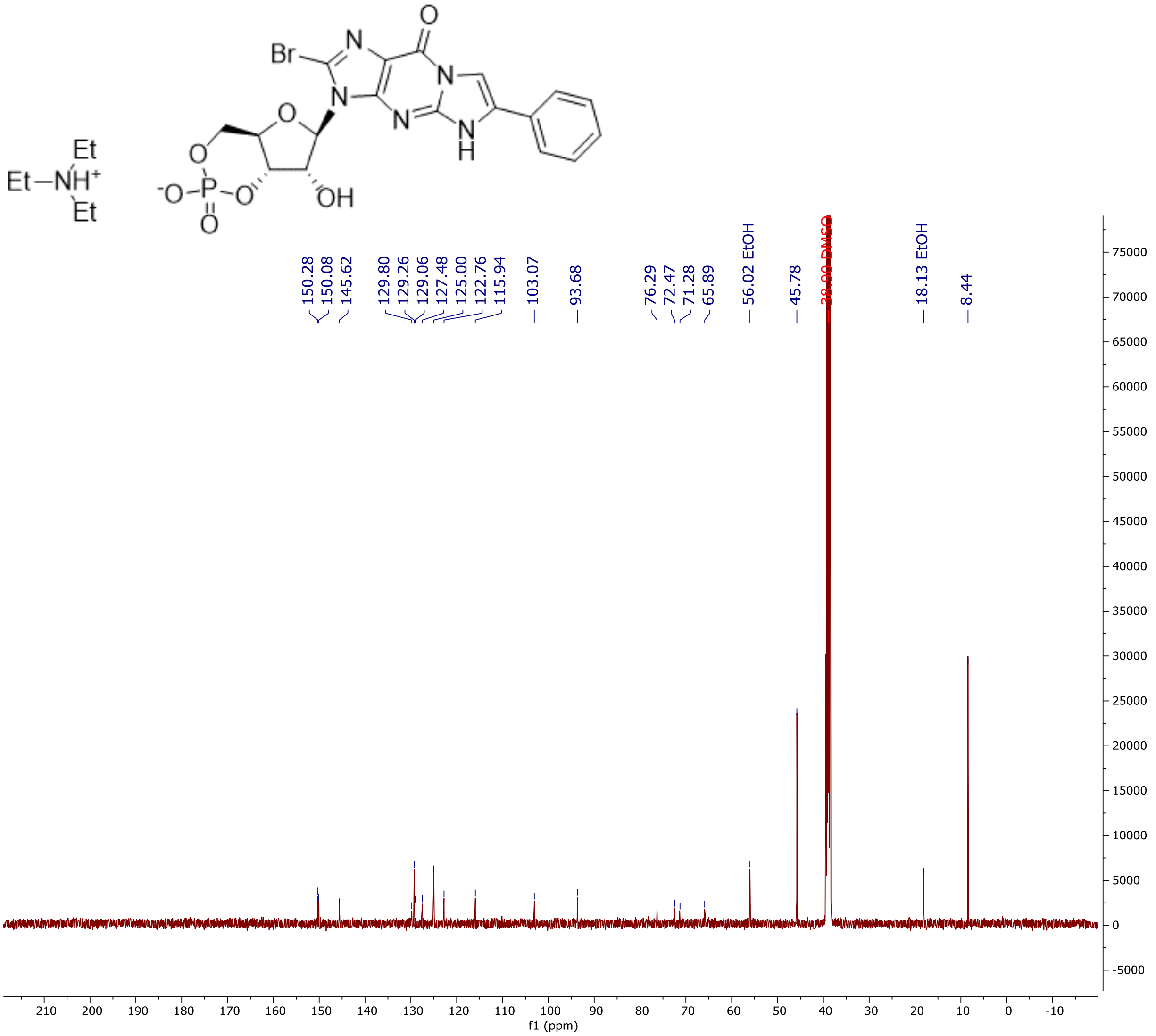

**Figure S36: $^{31}$P-NMR ($(CD_3)_2SO$, 203 MHz)**

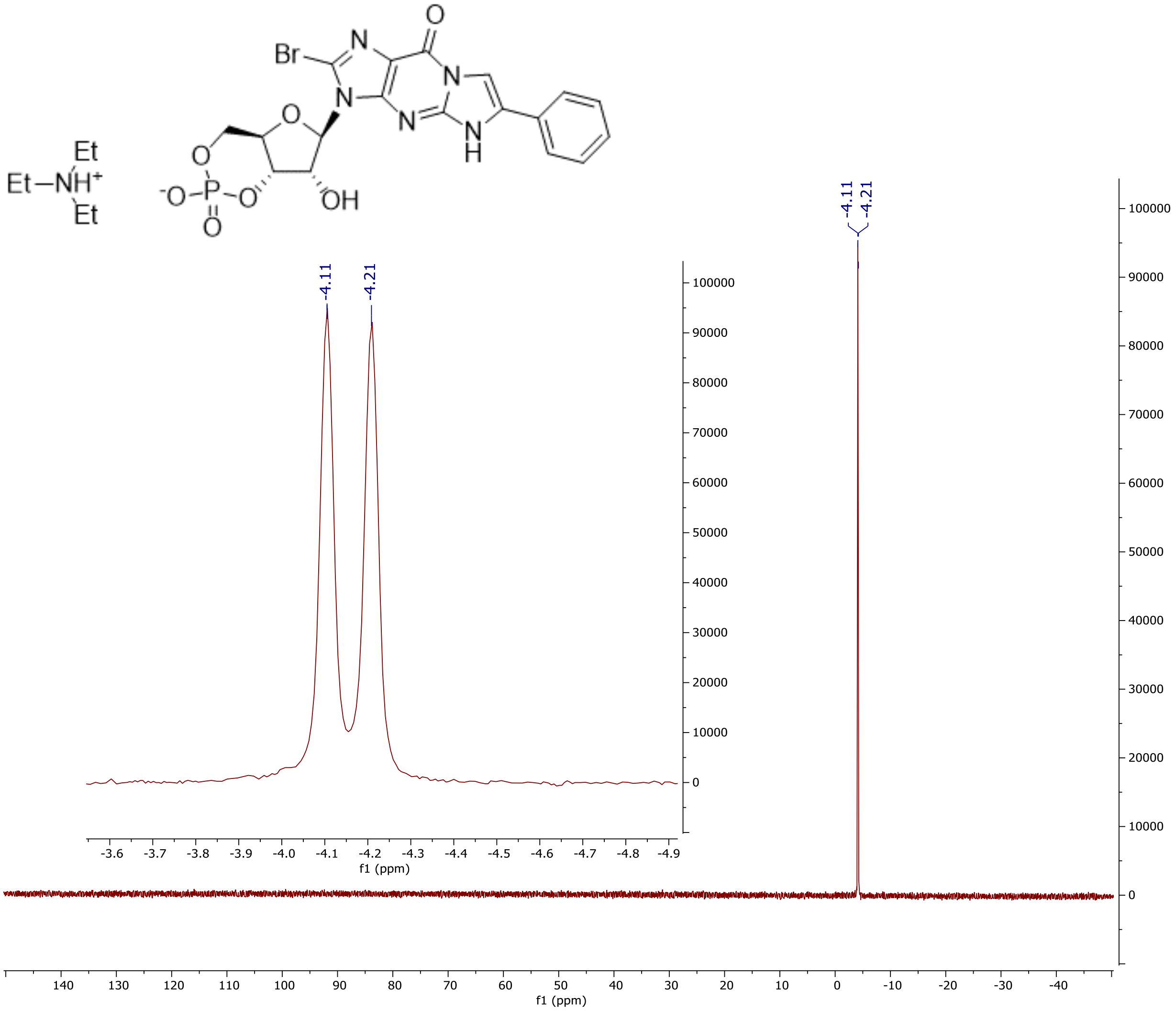

**Figure S37: $^{31}P\{^{1}H\}$-NMR ($(CD_3)_2SO$, 203 MHz)**

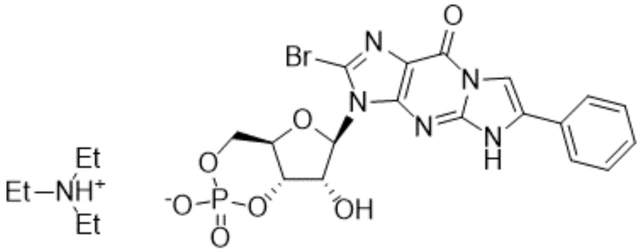

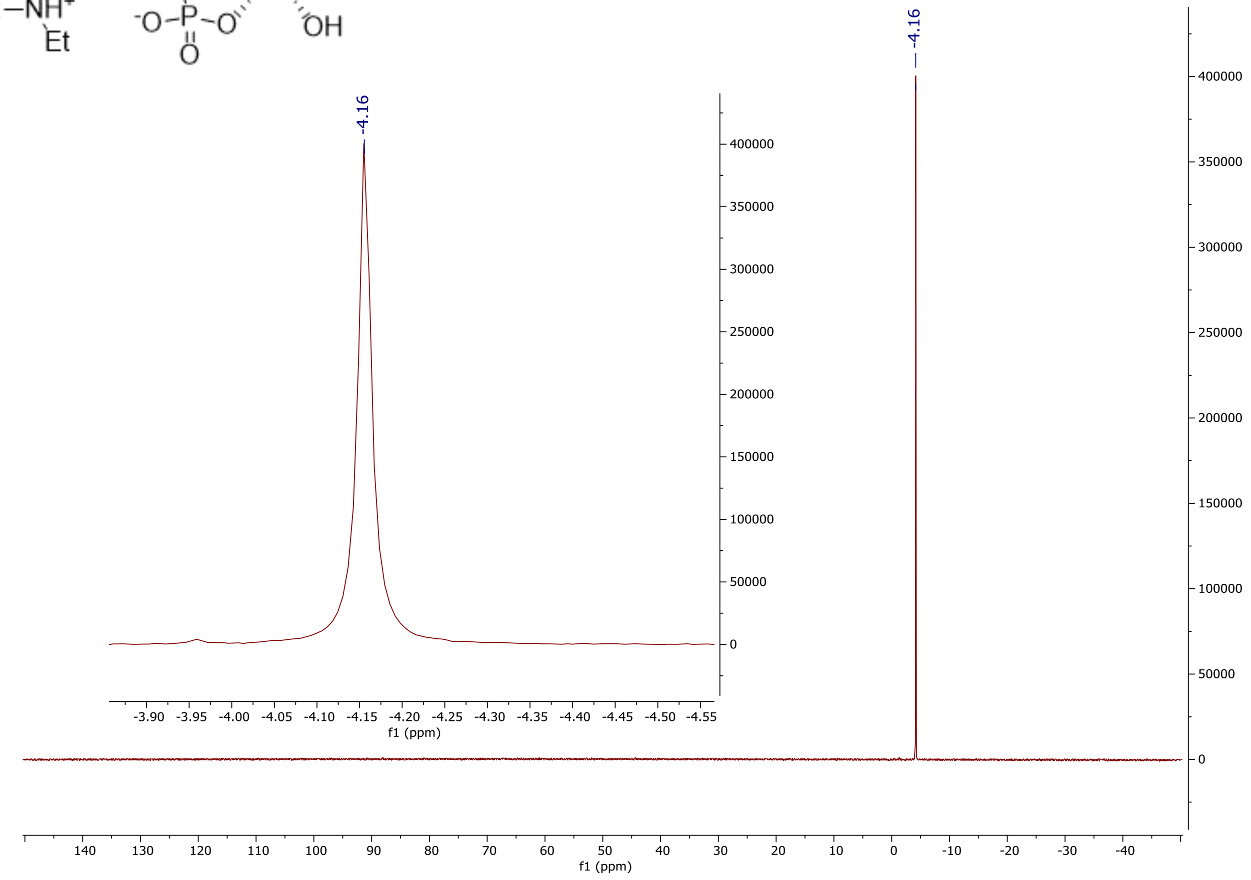

## Solubility data for dithio-CN03

**Table S1: Aqueous solubility at room temperature**

| Sample | mg / mL | pH of mixture |
|---|---|---|
| 1 | 1.160 | 4.7 |
| 2 | 1.198 | 5.6 |
| 3 | 1.160 | 5.9 |
| 4 | 1.169 | 6.3 |
| **Average** | **1.170** | **5.6** |
| SD | ±0.02 | ±0.7 |

**Figure S38: Temperature vs solubility curve.**

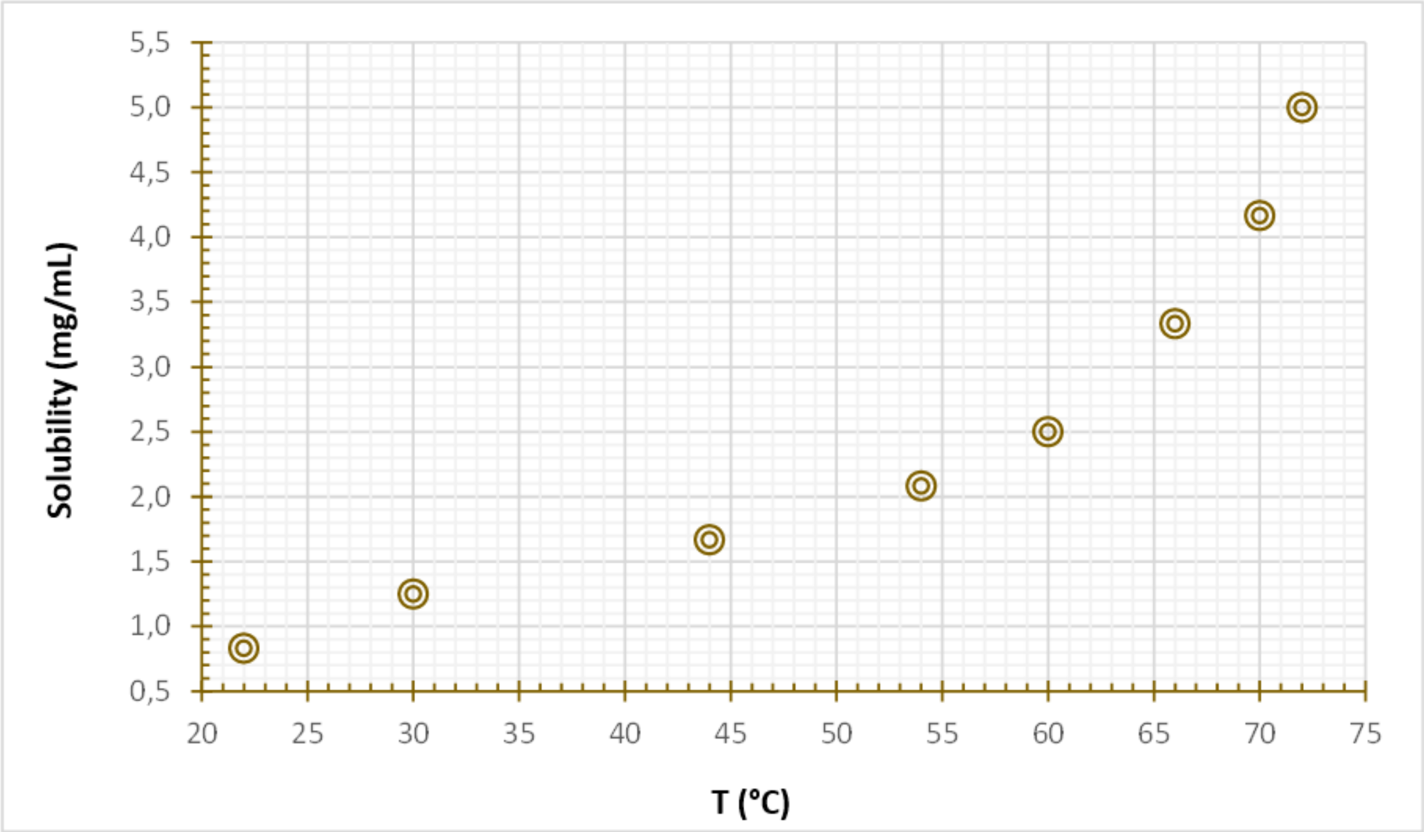

## Effects of vehicles on cell viability

**Figure S39: Dose response on 661W-A11 of $H_2O$ and DMSO.**

Cell viability assays of vehicles ($H_2O$ or DMSO) at different concentrations, expressed as percentage v/v in the culture medium. A red dashed line indicates the percentage of the vehicle in the experiments in which CN03 compounds were used at 50 µM to assess protective effects. Both $H_2O$ and DMSO had a minor influence on cell viability at the chosen concentration of vehicle.

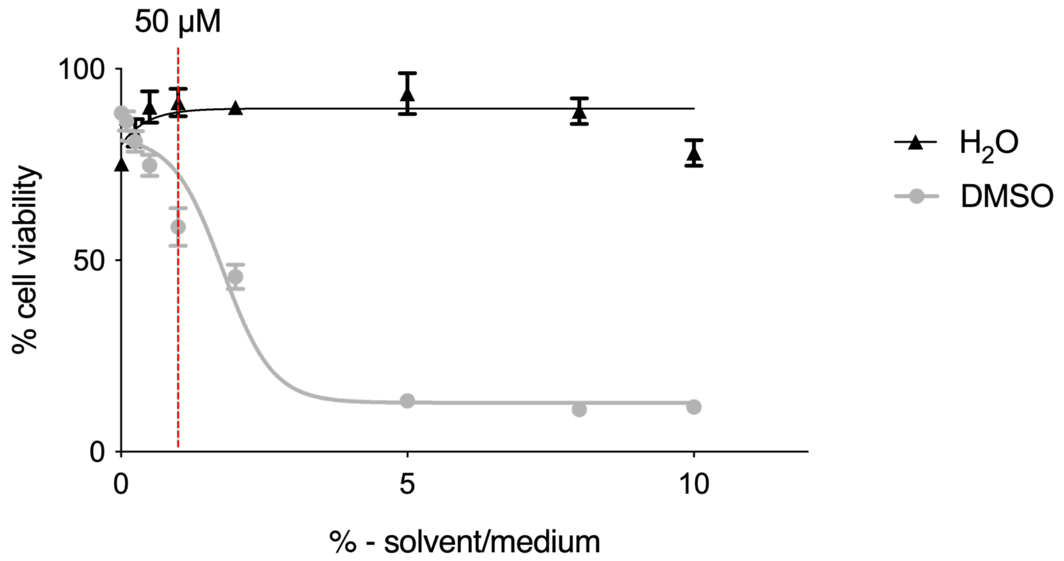

## References


(1) Pérez, O.; Schipper, N.; Bollmark, M. Preparative Synthesis of an R P -Guanosine-3′,5′-Cyclic Phosphorothioate Analogue, a Drug Candidate for the Treatment of Retinal Degenerations. *Org Process Res Dev* **2021**, *25* (11), 2453–2460. https://doi.org/10.1021/acs.oprd.1c00230.

(2) Pérez, O.; Schipper, N.; Leandri, V.; Svensson, P.; Bohlin, M.; Loftsson, T.; Bollmark, M. Crystal Modifications of a Cyclic Guanosine Phosphorothioate Analogue, a Drug Candidate for Retinal Neurodegenerations. *ChemistryOpen* **2023**, e202300141. https://doi.org/10.1002/open.202300141.
